# Development of a Contextualized, Research-Based Flemish Assessment Framework for Digital Care, Assistance, and Support: Delphi Study

**DOI:** 10.2196/88512

**Published:** 2026-04-15

**Authors:** Fien Buelens, Tom Seymoens, Jana Verplancke, Tom Van Daele

**Affiliations:** 1 Psychology and Technology Centre of Expertise Care and Well-Being Thomas More University of Applied Sciences Antwerp, Flanders Belgium; 2 Bachelor Social Work Artevelde University of Applied Sciences Ghent, Flanders Belgium; 3 Centre for Technological Innovation Mental Health and Education Queen's University Belfast Belfast, Northern Ireland United Kingdom

**Keywords:** quality assessment framework, digital, mental health, health, social work, care, professionals, organizations, Delphi study, technology, artificial intelligence, AI

## Abstract

**Background:**

The rapid evolution of digital technologies has transformed health, mental health, and social care, offering new modalities of digital care, assistance, and support through web-based platforms, mobile apps, extended reality, wearables, and artificial intelligence systems. Despite this proliferation, there is little consensus on what constitutes “high-quality” digital care. Challenges persist regarding data security, interoperability, accessibility, sustainability, and professional competence, whereas existing standards and regulations provide fragmented guidance.

**Objective:**

This study aimed to develop a contextualized, consensus-based quality assessment framework for digital care, assistance, and support in Flanders, Belgium. For this purpose, perspectives across technology, organizational processes, and professional competencies were integrated.

**Methods:**

The study used a multiphase design comprising (1) 10 expert interviews with Flemish government officials; (2) a narrative literature review of 303 peer-reviewed and gray literature sources; (3) a 3-round Delphi study with 50 experts across 5 domains (end users, facilitators, technology developers, deontology and ethics experts, and digital inclusion and media literacy experts); and (4) 4 complementary focus groups and 3 interviews with specialists in artificial intelligence, regulation, social work, mental health, and IT. The Delphi rounds gathered iterative feedback through open-ended elicitation, structured rating, and classification of quality criteria. Quantitative data were analyzed using descriptive statistics, whereas qualitative feedback was subjected to thematic analysis.

**Results:**

A total of 50 experts participated in round 1, a total of 40 (80%) participated in round 2, and 27 (54%) participated in round 3. Round 1 generated 577 unique quality criteria, consolidated into 26 clusters organized under 3 pillars: technology, organization, and professional competencies. The relative importance across pillars was balanced (mean score 37.29, SD 12.38 for technology; 33.33, SD 10.39 for professional competencies; and 29.80, SD 10.45 for organizations). Accessibility, reliability, and safety ranked highest for the technology; vision, quality monitoring, and infrastructure ranked highest for organization; and support, digital competencies, and ethics ranked highest for professional competencies. The finalized framework included 112 criteria, of which 35 (31.3%) were designated as optional and 77 (68.8%) were designated as minimum requirements. Focus groups and interviews validated the framework’s comprehensiveness and usability, emphasizing proportional implementation, user centrality, and alignment with European Union regulations. Stakeholders highlighted the need for tools, training, and governance mechanisms to ensure adoption and sustainability.

**Conclusions:**

This study produced a codeveloped, context-sensitive quality assessment framework that balances technological robustness, organizational readiness, and professional competence in digital care, assistance, and support. The framework can serve both as a quality safeguard and a developmental road map. Accompanying self-assessment and governance tools enhance practical applicability. Implementation success will depend on governmental support, resource allocation, and structured feedback loops. Future research should pilot the framework in real-world settings, assess its impact, and establish mechanisms for continuous updates to maintain relevance in a rapidly evolving digital landscape.

## Introduction

### Background

The proliferation of digital technologies has had a profound impact on the health, mental health, and social care sectors in recent years [1-3]. Digital technologies are used in services ranging from stand-alone online interventions to blended models that combine digital and face-to-face encounters, and they leverage a diverse toolset (eg, web-based platforms, mobile apps, extended reality, wearables, and artificial intelligence [AI]–enabled systems) for individuals; families; and specific groups such as children, youth, older adults, and persons with disabilities [4,5]. Therefore, the scope of their application is very broad, encompassing different topics, diseases, populations, and technologies. We use the umbrella term “digital care, assistance, and support” to refer to this integration of digital technologies into the provision, facilitation, or enhancement of health, mental health, social, and family-related services.

Because of their omnipresence in our current society, digital technologies are often also promoted as possible solutions to systemic issues in the health, mental health, and social care sectors, such as unmet needs, resource shortages, and personalized care [6,7]. However, their adoption does not come without barriers and challenges [8]. A major concern is safeguarding the quality and reliability of these technologies, which is often complicated by the rapid pace of technological innovation [9]. Key considerations include data security, system interoperability, accessibility for diverse user groups, and the sustainability of digital platforms [10]. Moreover, professionals must be specialized to effectively use these technologies as digital modalities often require additional competencies compared to in-person care [11]. For example, delivering online mental health therapy demands skills in digital communication, privacy management, and remote engagement. Furthermore, organizations and their managers also experience challenges implementing digital care, assistance, and support [12], for instance, organizing technical support for their professionals and end users.

Although European countries can fall back on a plethora of guidelines, regulations, certifications, and assessments, such as the International Organization for Standardization (ISO) guidelines, European Medical Device Regulation (MDR), European Union (EU) AI Act, and General Data Protection Regulation (GDPR) [13-15], there is no consensus on what really constitutes high-quality digital care, assistance, and support.

In addition, it often remains unclear how such quality requirements can be translated into everyday practice by the different actors involved in digital care, assistance, and support. Therefore, the framework developed in this study was intended not only as an assessment instrument but also as a practical guide for multiple user groups within the field. It can support professionals in reflecting on and improving digitally mediated practices; developers in designing and refining technologies in line with sector-specific quality expectations; and policymakers and organizations in shaping implementation, decision-making, and quality governance. In this way, the framework was conceived as a shared point of reference for the development, implementation, and evaluation of digital care, assistance, and support.

The need for such contextualization is particularly relevant in Belgium, where the organization of care, welfare, and support spans multiple policy and practice levels. In this study, we focused on Flanders because the framework was commissioned by the Flemish government and intended for use within the Flemish policy and practice context. This choice reflected the governance, organizational, and implementation setting in which the framework was meant to operate. The framework should also be distinguished from the Belgian validation pyramid for mobile health, which primarily supports the validation of and reimbursement for mHealth apps [16]. In contrast, this study aimed to develop a broader quality assessment framework for digital care, assistance, and support across a wider range of technologies, settings, and target groups. At this stage, the framework is intended primarily to support self-assessment, quality improvement, implementation, and governance in practice; any future reimbursement or formal policy applications fall outside the scope of this study.

### Objectives

Therefore, the primary objective of this study was to codevelop a consensus-based quality assessment framework for digital care, assistance, and support contextualized to practice in Flanders, Belgium. This focus reflected both the Flemish policy context in which the study was commissioned and the Flemish practical setting in which the framework was intended to be implemented. We pursued this objective through a literature review, a 3-round Delphi process, and stakeholder engagement via interviews and focus groups.

## Methods

### Study Design

**Figure 1:**
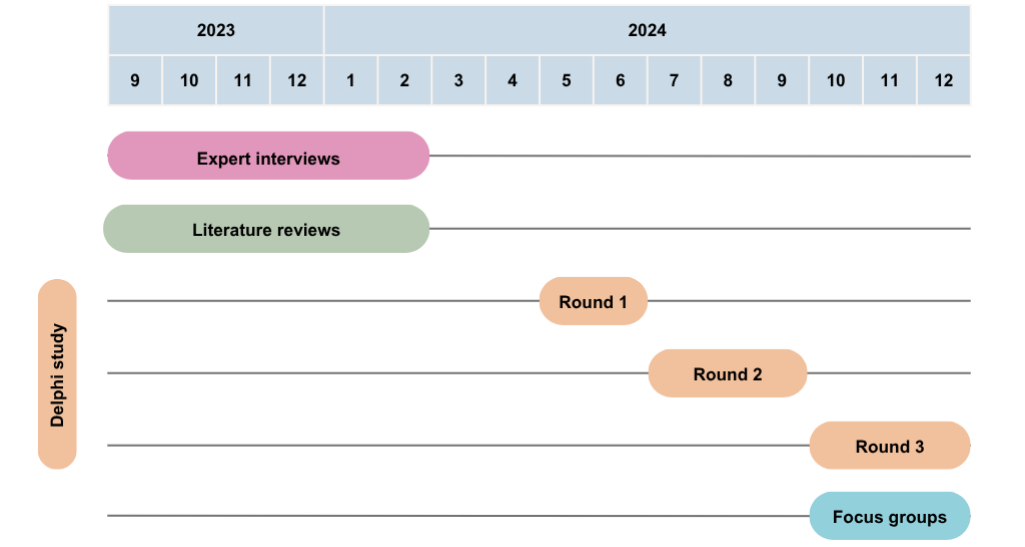
Timeline of the framework development phases.

The study consisted of 4 phases (Figure 1). First, 10 expert interviews were conducted with officials from the Flemish government, complemented by a narrative review of peer-reviewed and gray literature sources at the regional, national, and international level. Using this as a starting point, we subsequently conducted a 3-round online Delphi study that aimed to develop a consensus across a diverse panel of experts via an iterative, anonymous, and structured process with controlled feedback [17]. A full draft of the quality assessment framework was also presented and critically appraised in in-depth focus groups and follow-up interviews with specialists in AI, regulation, social work, mental health care, and IT.

### Expert Interviews

The preparatory phase commenced with semistructured online interviews with 10 experts from the Flemish government’s policy domain welfare, public health, and family. Participants were recruited within the steering committee of the project. The interviews focused on ongoing initiatives and anticipated needs within digital care, assistance, and support.

### Literature Review

In parallel with the interviews, we conducted an exploratory literature search between September 2024 and October 2025 to identify relevant peer-reviewed studies, regulatory documents, and other contextual sources. We searched bibliographic databases, including PubMed, Web of Science, and Scopus, using broad keyword combinations related to digital health, digital mental health, implementation evaluation, and digital competencies. The search strings included the following: (“digital health” OR eHealth OR mHealth OR “health app*” OR “mental health app*”) AND (evaluation OR assessment OR quality OR framework OR usability); (“digital mental health” OR “e-mental health” OR telepsychotherapy OR “online psychotherapy” OR “video consulting”) AND (implementation OR ethics OR accessibility OR regulation OR review OR sustainability OR adoption); and (“digital competenc*” OR “digital skill*” OR “digital literacy”) AND (“social worker*” OR “health care professional*” OR clinician* OR therapist*).

This search was complemented with gray literature, including institutional websites, policy documents, and selected practice-oriented materials, to help contextualize the academic literature and address differences in the availability of quality assessment publications across subfields of digital care, assistance, and support. The aim was two-fold: (1) to determine a limited number of frameworks that could offer a starting point for the all-encompassing quality assessment framework and (2) to find additional relevant sources that have the potential to contextualize and tailor these frameworks to end up with an all-encompassing quality assessment framework.

### Delphi Study

#### Participant Recruitment

A 3-round online Delphi study was set up to which 87 Flemish experts were personally invited. These experts were selected following an exploratory search and included to ensure stakeholder diversity across the following domains: (1) end users, (2) facilitators, (3) digital technology developers, (4) deontology and ethics experts, and (5) digital inclusion and media literacy experts. One reminder followed the initial invitation. Of the 87 invitees, 54 (62.1%) accepted, with each domain represented. They were subsequently asked to partake in each of the 3 rounds scheduled between May 2024 and January 2025. Each participant who completed round 1 was invited to every subsequent round.

#### Round 1

Participants were asked to respond to an open-ended prompt on what constitutes high-quality digital care, assistance, and support, deliberately without predefined criteria to capture the widest possible range of perspectives. Participants then organized their own proposed criteria by mapping them onto the study’s 3 conceptual pillars: technology, organization, and professional competencies. Subsequently, they provided meta-feedback on the 3-pillar structure (including suggestions for alternative subdivisions) and completed a 100-point allocation exercise across the pillars to indicate their perceived relative importance.

Additionally, various possible typologies of digital care, assistance, and support were discussed to structure the different quality criteria. As an example, participants were presented with the risk assessment of the EU AI Act and with the extent of professional human of involvement as defined in the ethical guidelines for technology-based suicide prevention programs to structure the quality criteria [14,18]. This led to a broader question: “Are there other typologies that can help differentiate between varying levels of quality criteria?”

#### Round 2

Round 2 invited participants to provide feedback on the introductory vision statement, which articulates the 3 pillars and foregrounds the user’s role in the quality assessment framework for digital care, assistance, and support. Participants identified strengths, weaknesses, and missing elements to refine the statement. Next, we presented the full set of criteria with definitions. Experts assigned each criterion to 1 of the 3 pillars: technology, organization, or professional competencies. Moreover, they were asked to allocate points to indicate each criterion’s perceived importance.

Finally, we examined a proposed typology for the criteria structured along 2 axes: degree of interactivity and level of risk assessment as this resulted from the previous round (see the Results section). The resulting matrix comprised 9 categories, each representing a distinct combination of these dimensions. Experts evaluated the usefulness and validity of this categorization for differentiating between levels of quality criteria.

#### Round 3

In round 3, the experts received the complete draft of the quality assessment framework, comprising (1) an introductory text articulating the vision (rationale, context, and explanatory notes), (2) an itemized list of 119 criteria mapped to the 3 pillars, (3) a section on cross-pillar interdependencies, and (4) user guidance on applying the framework.

Participants engaged with the draft in 2 ways. First, they provided annotated textual feedback on content, structure, and wording. Second, they reflected on the relevance of each criterion. For this reflection, they categorized each criterion as either minimum, meaning essential to ensure quality; optional, meaning desirable but not strictly necessary; or unnecessary, meaning that it could be omitted without compromising quality. In addition, open-ended questions probed the framework’s purpose and structure, clarity and completeness, implementation feasibility, and contextualization.

Finally, we circulated the finalized framework (Multimedia Appendix 1) to all experts. Via an online form, participants were asked to indicate whether they agreed with the current version of the framework.

### In-Depth Focus Groups and Interviews

In parallel with round 3 of the Delphi process, online focus groups and individual interviews were conducted to test topic-specific coverage and implementation issues alongside the Delphi study. Participants were selected for their relevant expertise and involvement in digital care, assistance, and support across policy, practice, technology, and ethics or legal domains.

Before their session, participants received the draft framework and provided annotated comments; completed the “minimum,” “optional,” or “unnecessary” classification used in the Delphi study; and answered brief open-ended questions. Sessions opened with informed consent and a short overview of the framework, followed by a structured discussion on (1) comprehensibility (goals, structure, and wording), (2) completeness and consistency of the criteria (including ethical and legal aspects), and (3) implementation feasibility (roles, support needs, and tools). Recordings, annotations, and researcher notes were collated into session summaries; where changes to the framework were warranted, they were implemented and explicitly flagged when diverging from the original source frameworks.

### Analyses

For the expert interviews, a thematic analysis was conducted. Additionally, the results of the literature review were derived using an exploratory thematic analysis in Citavi (Swiss Academic Software GmbH) [19], complemented by a categorization based on source type, thematic focus, geographical scope, and data characteristics. The various rounds of the Delphi study were assessed through a combination of thematic analysis, clustering techniques, and descriptive statistical methods.

### Ethical Considerations

Ethics approval from a medical ethics committee was not required under Belgian national law as operationalized by the Ethics Committee Research University Hospitals Leuven/KU Leuven [20]. All participants provided informed consent for each data collection wave of the Delphi study and before each focus group. All participants who wished to receive a voucher of EUR €30 (US $34.76) for Bol, a Dutch-Belgian online retail platform, were provided with one after completing their participation. Data were processed in accordance with the GDPR [15] and in line with the privacy policy of Thomas More University of Applied Sciences [21].

## Results

### Expert Interviews

The semistructured online interviews with 10 government experts found respondents calling for clearer regulation, guidance, and a coherent framework. They also stressed the need to preserve room for locally tailored digital services. Experts widely judged the current approach to be insufficient, citing gaps in knowledge and expertise, limited support, and the absence of an overarching framework. Key concerns concentrated on legal compliance (eg, GDPR) and secure communication. They furthermore stressed accessibility and inclusiveness as nonnegotiable quality criteria, noting barriers related to language, culture, age, disability, and digital literacy. Collectively, the findings pointed to the need for a practical, inclusive, and codeveloped framework with service providers that accounts for sector-specific contexts and diverse target groups.

### Literature Search

In the subsequent literature search, 303 sources were identified and coded by source type, thematic focus, geographic scope (regional, national, and international), and data characteristics (Multimedia Appendix 2). From this, 3 overarching pillars emerged to structure the draft framework: technology, organization, and professional competencies. As became clear from the literature and the interviews, the quality of digital care, assistance, and support depends not only on the quality of the technology but also on its contextualization and on the training of professionals. To substantiate each pillar, we anchored the draft in three established source frameworks: (1) ISO/TS 82304-2:2021, “Health software—Part 2: Health and wellness apps,” an internationally recognized ISO framework for assessing the quality and reliability of health apps; (2) the organization domain of the Health Technology Assessment Core Model, a European framework developed within the European Network for Health Technology Assessment to address organizational conditions, workflows, and implementation factors relevant to health technologies; and (3) the framework for digital competences of professional social workers, a practice-oriented framework developed in the Flemish and Belgian social work context that specifies the digital competencies required for blended and digitally supported professional practice [12,22,23].

These 3 source frameworks were selected because they are established and authoritative within their respective domains; are recognized at the international, European, or local professional field level; and provide a sufficiently strong conceptual and empirical basis for developing a framework tailored to the broader Flemish context of care, assistance, and support.

### Delphi Study

#### Participant Characteristics

The first Delphi round resulted in 57.5% (50/87) of responses. Table 1 provides an overview of the distribution of expertise among the respondents. As previously mentioned, all experts who participated in the first round were invited to subsequent rounds. The response rate in the second round was 80% (40/50), which declined to 54% (27/50) in the third round.

**Table 1:** Number of participants taking part in each round and the responses per round, with an additional breakdown within each round of the different areas of expertise and their percentages.

Expertise	Invited experts (n=87), n (%)	Delphi round 1 (n=50), n (%)	Delphi round 2 (n=40), n (%)	Delphi round 3 (n=27), n (%)
End users	22 (25.3)	10 (20)	5 (12.5)	4 (14.8)
Facilitators	20 (23)	10 (20)	11 (27.5)	7 (25.9)
Technology developers	19 (21.8)	13 (26)	10 (25)	8 (29.6)
Deontology experts	18 (20.7)	12 (24)	9 (22.5)	7 (25.9)
Digital inclusion and media literacy experts	8 (9.2)	5 (10)	5 (12.5)	1 (3.7)

In addition to the Delphi study, 4 focus groups and 3 individual interviews were conducted. The focus group with members of the steering committee included 11 participants, the ethics and legal focus group had 5 participants, the information and communications technology developer focus group consisted of 4 participants, and the well-being professional focus group included 5 participants.

#### Round 1

The open-ended elicitation yielded 577 distinct criteria from 50 participants. After thematic analysis, these were consolidated into 26 clusters and cross-checked against insights from the literature study.

In the 100-point score allocation exercise to indicate the relative importance of the 3 pillars, the distribution was broadly balanced: the technology pillar was considered the most important (mean 37.29, SD 12.38), followed by professional competencies (mean 33.33, SD 10.39) and organizations (mean 29.80, SD 10.45). Participants also proposed additional pillars, most frequently user, which was mentioned by 18 participants and governance, which was mentioned by 10 participants; other suggestions appeared only once. In response, the importance of the user and the need for attention to governance of a quality assessment framework were explicitly addressed in the framework’s introduction to clarify their overarching relevance.

Finally, respondents brainstormed typologies to differentiate criteria. The most frequently suggested was interactivity (n=20), followed by risk taxonomy (n=6). Nonobligation (voluntariness of use) and anonymity were each mentioned 3 times, with all other typologies mentioned once. As no single typology received a clear majority, this question was carried forward to the next round for further exploration.

#### Round 2

Experts first reviewed the introductory section. Of the 40 respondents, 37 (92.5%) expressed full agreement, whereas 3 (7.5%) disagreed. Highlighted strengths included user centrality, autonomy, flexibility to accommodate evolving insights, interdependencies among pillars, and a graduated (proportional) approach. Proposed areas for improvement included ambiguity regarding end user responsibility, sharper wording for the 3 pillars, absence of broader societal context, and overall clarity and structure. Where feasible, these suggestions were incorporated into a revised introduction.

Participants then categorized all criteria under the 3 pillars and allocated importance points at the criterion level. Table 2 reports, for each pillar, the top 3 criteria, including the number of respondents endorsing each and the mean points assigned. When comparing these results with the source frameworks from the preparatory research, a considerable overlap was observed between the criteria grouped under the 3 pillars.

**Table 2:** Top 3 criteria for each pillar, including the number of participants who mentioned each criterion and the average points allocated (n=40).

Pillar and criterion		Participants, n (%)	Score (0-100), mean (SD)
**Technology**			
	Accessibility	29 (72.5)	20.48 (8.79)
	Reliability	26 (65)	23.23 (8.04)
	Safety	24 (60)	20.38 (6.28)
**Organizations**			
	Vision	32 (80)	26.19 (8.07)
	Quality monitoring	24 (60)	19.92 (6.31)
	Infrastructure	18 (45)	19.28 (3.54)
**Professional competencies**			
	Support	27 (67.5)	21.74 (8.19)
	Digital competencies	26 (65)	24.00 (8.26)
	Ethics	21 (52.5)	22.57 (9.02)

The final aspect of this round focused on the typology. A total of 85.3% (29/34) of the participants agreed with the proposed typology, which was structured around interactivity and risk taxonomy. Despite this strong support and positive feedback, several concerns were raised. These included the abstract nature of the terminology; the difficulty of making objective classifications; the complexity of having 9 distinct categories; and the challenge of applying this typology across the entire field of digital care, assistance, and support. As a result, the typology was incorporated into the quality assessment framework as a suggested vision for evaluating specific interventions within specific contexts. It was not implemented as a universal classification system for all criteria across the framework or the broader field.

#### Round 3

Experts reviewed the full draft and provided annotated comments on content, structure, and wording. The feedback clustered primarily around organizational implementation and professional roles and competencies, with recurrent requests to simplify language and strengthen user participation. We implemented changes where feasible to improve clarity, remove minor redundancies, and standardize phrasing across the 3 pillars and cross-pillar sections.

Participants then classified each of the 119 criteria as minimum, optional, or unnecessary; applying the prespecified rule (flagging as optional if more than two-thirds of the participants selected “optional,” removing if more than two-thirds selected “unnecessary,” and otherwise retaining as a minimum criterion) yielded 46 (38.7%) optional items and no deletions (Multimedia Appendix 3).

Finally, responses to brief open-ended questions on purpose and structure, clarity and completeness, implementation feasibility, and contextualization led to 2 targeted additions: a self-assessment instrument that supports a growth pathway and selective completion (pillars need not be completed at once) and the explicit embedding of a proportionality typology in the introduction using risk stratification and degree of interactivity as practical lenses to tailor criterion selection to organizational context.

After the Delphi round 3, the framework contained 119 criteria across 3 pillars (n=53, 44.5% in the technology pillar; n=28, 23.5% in the organizations pillar; and n=38, 31.9% in the professional competencies pillar), of which 73 (61.3%) were classified as minimum and 46 (38.7%) were classified as optional criteria.

### In-Depth Focus Groups and Interviews

Concurrent with round 3, we engaged experts across policy, well-being practice, technology, and ethics and legal domains in 4 online focus groups and interviews to assess topic coverage and implementation, focusing on comprehensibility, completeness, consistency, and implementation feasibility.

The focus groups largely confirmed the structure and content of the framework while highlighting clarifications needed for usability and proportional adoption. Participants called for a sharper articulation of target users and use cases, a strong separation between binding minimum requirements and guiding optional criteria, and practical guidance tailored to organizational and developer contexts. They also emphasized that the framework should not be understood solely as a formal assessment tool but also as a practical guide to support reflection, implementation, and quality improvement in practice. Input from experts with backgrounds in law and ethics emphasized the need for (1) direct legal signposting rather than paraphrasing; (2) technology-neutral and use case–specific wording to avoid rapid obsolescence; and (3) a proportional, comply-or-explain orientation that permits reasoned trade-offs across principles while preserving legal certainty. Input from technological experts underscored the need for (1) clear accountability and maintenance expectations across the life cycle, (2) feasible baselines for small or medium-sized enterprise and start-ups, and (3) signposting to recognized norms (eg, interoperability and accessibility) alongside lighter risk management baselines as stepping stones. Finally, well-being professionals stressed (1) the translation gap between technical language and day-to-day practice, (2) the central role of service users, and (3) the value of supporting roles to embed the framework. Common implementation needs converged across groups: training professionals, governmental support and resources, simple implementation tools (eg, checklists, templates, and audits), and a feedback loop after launch.

The interviews brought forth similar themes and added emphasis on alignment with EU-level initiatives (eg, the MDR, EU AI Act, GDPR, and European Health Data Space regulation [13-15,24]) and the expectation that technology providers and developers justify state-of-the-art technical and organizational measures without hard-coding specific technologies.

Building on the combined input from Delphi round 3 and these focus groups and interviews, the framework was adapted. This adaptation reflected a qualitative streamlining: 9 new criteria were added (eg, on avoiding dark patterns, affordability for clients, and explicit alignment with EU legislation such as the European Health Data Space), whereas conceptually overlapping criteria were merged (several clusters of 2 to 3 similar items combined into 1 broader criterion) and 9 redundant items were removed because they were fully covered elsewhere. As a result, the final framework is slightly shorter but more precise and up-to-date in legal terms.

### Resulting Quality Assessment Framework

All consolidated efforts resulted in a contextualized quality assessment framework for digital care, assistance, and support in Flanders, Belgium (Multimedia Appendix 1) with a final set of 112 criteria (n=53, 47.3% in the technology pillar; n=23, 20.5% in the organizations pillar; and n=36, 32.1% in the professional competencies pillar), with 77 (68.8%) minimum and 35 (31.3%) optional items. Three established source frameworks were taken as a starting point and modified using input from key stakeholders. All these modifications were made to be traceable: each departure from or addition to a source framework is explicitly referenced (either to existing literature or to insights, requests, or recommendations from stakeholders during the course of this study) to ensure provenance, auditability, and transparency regarding the rationale for adaptation. Beyond its assessment function, the resulting framework is intended to support reflection, quality improvement, implementation, and decision-making across different contexts of digital care, assistance, and support.

## Discussion

### Principal Findings

This study aimed to develop a contextualized quality assessment framework for digital care, assistance, and support in Flanders, Belgium. Drawing on initial interviews with policymakers and a literature review, a 3-round Delphi study with key experts was conducted and complemented by focus groups and interviews. Together, these methods yielded a framework that synthesizes perspectives across 3 pillars: technology, organizations, and professional competencies.

The results of the Delphi study demonstrated a relatively balanced distribution of importance across the pillars, with technology receiving slightly higher priority. This balance underscores the interdependence of technological robustness, organizational aspects, and professional competencies in ensuring the quality of digital care, assistance, and support. Furthermore, the classification of criteria into minimum and optional highlights a dual function of the framework: on the one hand, it provides essential requirements to safeguard quality, and on the other hand, it creates a growth path for continuous improvement. This graded approach enhances the usability of the framework for diverse stakeholders, ranging from small organizations to larger institutions.

Throughout the focus groups and interviews, participants emphasized the importance of the introductory section of the framework, particularly the central role assigned to the user. This focus on user needs, autonomy, and accessibility aligns with broader European and international discourses on digital inclusion and human-centered design [25]. Moreover, the typology based on risk and interactivity offered a pragmatic lens for contextualizing the application of criteria even though participants expressed concerns about its complexity and operationalization for the entire framework. Experts also highlighted the necessity of clarifying terminology, ensuring a clear distinction between binding and guiding criteria, and aligning the framework with existing regulations (eg, GDPR, EU AI Act, and MDR). Importantly, the inclusion of frontline practitioners stressed the need for translation into practice-oriented tools and language. This reinforces that the framework not only provides conceptual clarity but also that derivative instruments were developed.

When compared with existing European assessment and reimbursement frameworks for digital health, this framework shows both substantial overlap and a distinct contribution. At the level of technology assessment, the core domains identified in this study are largely consistent with those found in other frameworks already in use. For example, the German digital health application fast track assesses product qualities such as data protection, information security, interoperability, quality, and user-friendliness in addition to evidence of a positive health care effect [26]. The Belgian validation pyramid similarly focuses on CE marking, GDPR compliance, interoperability, connectivity, usability, and reimbursement requirements for mobile health apps [27]. In Finland, Digi-HTA combines traditional health technology assessment domains such as safety, effectiveness, and cost with digital-specific domains, including usability and accessibility, data security and protection, interoperability, and technical stability [28]. Similarly, the French Prise en Charge Anticipée Numérique (PECAN) pathway is primarily oriented toward reimbursement of sufficiently mature digital medical devices, with emphasis on clinical and/or organizational benefit, as well as compliance with data protection, interoperability, and security requirements [29].

The added value of this framework lies not only in broadening the scope of assessment but also in confirming the relevance of these established technology-related domains within the wider field of digital care, assistance, and support. The expert input gathered in this study indicates that criteria such as usability, interoperability, data protection, and security remain highly relevant in this broader context. At the same time, the framework extends beyond a narrow product assessment or reimbursement logic by integrating 2 domains that are much less explicitly elaborated on in most existing frameworks, namely, organizational conditions and professional competencies. In that sense, it conceptualizes quality as a contextual, practice-based, and sociotechnical issue. This is particularly relevant for digital care, assistance, and support, where quality depends not only on the technical properties of a tool but also on how it is embedded in organizational workflows, governance arrangements, and the capabilities of the professionals who use it. A further contribution of the framework is its contextualization to the Flemish care, welfare, and support landscape while remaining aligned with broader European legal and regulatory requirements. Within the scope of this study, the framework was developed primarily to support quality assessment, self-reflection, implementation, and quality improvement in practice rather than as a reimbursement model. At the same time, its possible future policy applications fall beyond the scope of this study.

### Strengths, Limitations, and Considerations

A key strength of this study and the resulting quality assessment framework is its interdisciplinary and participatory approach. The diversity of expertise in the Delphi panel, focus groups, and interviews facilitated the development of a shared language and common understanding across different domains of digital care, assistance, and support, whereas the iterative, participatory feedback fostered high engagement and ownership among participants.

Nevertheless, limitations must be acknowledged as well. First, the starting point of the quality assessment framework was based on an exploratory literature review rather than a formal systematic review. Although this approach allowed us to include a broad range of peer-reviewed, regulatory, and gray literature sources, the search and, more specifically, the selection of the source frameworks could have been more structured and standardized. A second limitation is that the time-intensive nature of the process may have deterred broader involvement of stakeholders. This was also reflected in the Delphi method, which does ensure consensus but saw participation rates decline across rounds, potentially limiting representativeness. A third limitation is that the framework is contextually grounded in Flanders. Although it remains sensitive to international developments, this may limit its applicability to other regions as adaptation to local context remains key for technologies and frameworks alike [30]. Nonetheless, for the technology pillar, another recent adaptation of the European Committee for Standardization ISO/TS 82304-2 standard in Catalonia has demonstrated that this can be done in a relatively straightforward process [31].

Furthermore, it remains to be seen how the quality assessment framework will be implemented in practice. Real and lasting impact will only be achieved through proper governance strategies. Finally, digital care, assistance, and support is a rapidly evolving field that necessitates periodic updates to maintain the relevance and validity of the framework. This underscores the need for a structured governance approach that provides clear roles, oversight, and periodic review cycles to keep the framework current and trustworthy.

### Conclusions

The framework provides a valuable foundation for assessing and guiding the quality of digital care, assistance, and support in Flanders. To support its implementation, accompanying tools have already been developed, including a practical self-assessment instrument and governance guidelines. These resources aim to facilitate translation into daily practice by offering organizations concrete means to apply the framework in a structured and proportional way. Nevertheless, successful adoption will also depend on adequate governmental support, allocation of resources, and the establishment of sustainable feedback mechanisms. Future research should focus on piloting these tools and the framework in diverse real-world contexts, evaluating their feasibility and impact, and identifying strategies for keeping them up-to-date in light of rapid technological and societal developments. In addition, future studies could explore how the framework may be updated or adapted to the specific contexts and needs of other regions.

## Data Availability

The qualitative data generated and analyzed during this study are not publicly available due to confidentiality and anonymization considerations. The quantitative datasets are available from the corresponding author on reasonable request.

## Appendix

Multimedia Appendix 1 Quality assessment framework.

Multimedia Appendix 2 Overview of the literature.

Multimedia Appendix 3 Classification criteria.

## Abbreviations

AI: artificial intelligence
EU: European Union
GDPR: General Data Protection Regulation
ISO: International Organization for Standardization
MDR: Medical Device Regulation
PECAN: Prise en Charge Anticipée Numérique
