# Supplementary material for: Development of a Contextualized, Research-Based Flemish Assessment Framework for Digital Care, Assistance, and Support: Delphi Study
Source: JMIR Form Res. 2026 Apr 15;10:e88512. doi: 10.2196/88512 (PMC13129510; doi:10.2196/88512)
Supplement: Multimedia Appendix 1 [file formative_v10i1e88512_app1.pdf]

QUALITY ASSESSMENT FRAMEWORK FOR DIGITAL CARE, ASSISTANCE AND SUPPORT IN THE DOMAIN OF WELFARE, HEALTH AND FAMILY

Fien Buelens, Tom Seymoens, Jana Verplancke and Tom Van Daele

Table of contents

Table of contents 2
1 Beforehand 4
2 How is this document structured? 4
3 Introduction 5
3.1 Who is the quality framework intended for? 5
3.2 Users as a starting point 5
3.3 The role of other parties in the quality framework 6
3.4 Three pillars 7
3.5 How do you use this quality framework? 7
3.5.1 Minimum quality criteria and optional criteria 7
3.5.2 Context 7
4 Pillar technology 9
4.1 Provider technology 9
4.2 Purpose of the technology 9
4.2.1 Requirements 9
4.2.2 Added value 10
4.2.3 Support and contact 10
4.3 Convenience 10
4.3.1 Accessibility 10
4.3.2 Usability 11
4.4 Deontology 11
4.4.1 Risks 11
4.4.2 Ethics 12
4.4.3 Safety 12
4.5 Robust construction of the technology 13
4.5.1 Technical robustness 13
4.5.2 Interoperability 15
5 Pillar organizations 16
5.1 Providing digital care, assistance and support 16
5.1.1 Technology affects the operation of the organization 16
5.1.2 User Path 17
5.1.3 Engage users, professionals, and key others 17
5.1.4 Training and supporting professionals 17
5.1.5 Guaranteeing and controlling quality with a system 18
5.2 Cost of the process 18
5.2.1 Cost of purchasing, installing, and maintaining the technology 18

5.2.2 Impact of technology on needs 18
5.2.3 Impact of the technology on the budget 18
5.3 Management 19
5.4 Adoption of technology 19
6 Pillar of professionals 20
6.1 Promoting digital inclusion and competences 20
6.1.1 Promoting digital inclusion 20
6.1.2 Strengthening their own digital competencies 21
6.2 Providing digital care, assistance and support 21
6.2.1 Consciously selecting technology 21
6.2.2 Providing digital care, assitance and support to the users and their network 21
6.2.3 Securely manage digital client data 22
6.3 Helping to shape digital care, assistance and support in the organization 22
6.3.1 A vision on digital care, assistance and support 22
6.3.2 Constructively evaluating critical digital care, assistance and support 22
7 Concepts 24
8 Sources **Error! Bookmark not defined.**
9 Recognition **Error! Bookmark not defined.**
10 Citation suggestion 29
11 Contact & info 29

Beforehand

As part of the Centre for Welfare, Public Health and Family, Thomas More University of Applied Sciences and Artevelde University of Applied Sciences developed a quality framework for digital care, assistance and support on behalf of the Department of Care. This assignment stems from the need for clarity about the (minimum) quality criteria for governments, organizations, providers, developers, professionals and users.

The demand for unambiguous and transparent criteria has increased in recent years, given an increase in (European) legislation, directives, frameworks, standards, technological developments, user expectations and signals from the field.

This quality framework was scientifically substantiated by a literature study, interviews, and experiences of experts, professionals and users. More information about our methodology can be found in the final report (in Flemish). You can download this via www.steunpuntwvg.be/publicaties.

How is this document structured?

- The introduction provides the context of the quality framework. What was the reason? Who is it for? You will also receive information on how to use the quality framework.
- The next three chapters describe these three pillars and their criteria in detail.
- In the boxes you get more background, clarification and examples.
- You can click on the words that are underlined. At the end of the glossary you will find a definition.
- Finally, you will find a list of sources with the various sources on which the quality framework is based.

Introduction

Who is the quality framework intended for?

This framework must guarantee the quality of digital care, assistance and support in their development, implementation and use. It focuses on

- IT developers of digital care, assistance and support
- professionals, volunteers, staff members, IT managers, etc.
- Policy officers within organizations and governments

And all this in the various sectors within welfare, public health and family. Think of elderly care, youth care, health care, family support, mental health care, general welfare work and support for people with disabilities.

But the criteria can be applied more broadly. The framework provides common guidelines for better quality, but also common and clear concepts across the different domains. It can thus contribute to a better understanding and clear communication between, for example, government and professionals, organizations themselves or with providers of IT applications.

What do we mean by digital care, assistance and support?

- all technology such as apps, websites, chatbots, virtual reality, wearables, and AI
- for citizens, clients, patients and their network
- to maintain or improve their well-being and health

Users as a starting point

In this context, users are clients, patients and their network, family members and informal caregivers. Their perspective is central to assessing the quality of a digital offering.

These are the general principles:

- The expectations of the users. What do they need? What are their abilities?
- Users have the right to express their opinion on the design and use of the digital offer.
- They have control over their own trajectory and decide what information to share with the other parties.
- Users can choose between a digital and non-digital offering, depending on what they expect or need and their capabilities. The digital offer is an option.
- Information about and from the users is always confidential and secure, and according to the relevant legislation, such as the GDPR. This data may only be shared or processed with the explicit consent of the user. Users also know that they have the right to view, modify or delete the data.
- Users have the right to technology that is understandable and accessible. And that takes into account their skills and limitations, for example language skills and literacy. (9)

The framework pays special attention to the most vulnerable users such as children, young people, people with disabilities, people with low digital literacy and people who are incapacitated. For them, extra protective measures are needed when using a digital offer. Think, for example, of permission from legal representatives, or extra guidance on the use of the technology.

Users also have some duties:

- They must use the digital offer correctly and in accordance with the law. They comply with the terms of use and they do not engage in any unlawful activities. For example, they do not share confidential information without explicit permission from a professional.
- Are they using the digital offer incorrectly or not according to the law? For example, do they spread malicious content? Or do they violate the rights of others? Then users can be held liable.

Do certain groups or users lack certain skills or knowledge? Or do they not have access to the digital offer? Then professionals and government can help with that. For example:

- by strengthening those skills, such as digital literacy and health literacy
- by offering a support network
- by continuing to provide an additional non-digital offer

The role of other parties in the quality framework

Various parties influence the quality of digital care, assistance and support: the professionals, the organisations, the developers, the government and the society.

- Professionals and volunteers act ethically and correctly, according to their competences and according to the rules of their profession. The general rules for professional secrecy continue to apply. But there are also specific rules, for example for storing data.
- Organizations ensure that the quality framework is in line with their general quality policy.
- Developers involve users, professionals and other parties in the development of the digital offering. The technology must grow with the changing needs and feedback from users and professionals.
- The government provides the preconditions for high-quality digital care, assistance and support. For example, organizations or professionals are recognized or subsidized for:
  - skills of the users.
  - infrastructure.
  - make the digital offer available to everyone, regardless of socio-economic background or skills.

  Rules and policy choices have a strong influence on quality. The quality framework can therefore not be seen separately from the overarching European, Belgian, Flemish and local policy.
- The needs of people and society are changing. The quality framework provides direction, but it must be flexible enough to respond to technological and digital progress. General principles apply, but the implementation can change over time and due to new digital possibilities.

Three pillars

High-quality digital care, assistance and support is based on three central pillars: technology, organisations and professionals. This framework links criteria to each of these pillars. These criteria describe how technology, organisations and professionals can guarantee the quality of the digital offer.

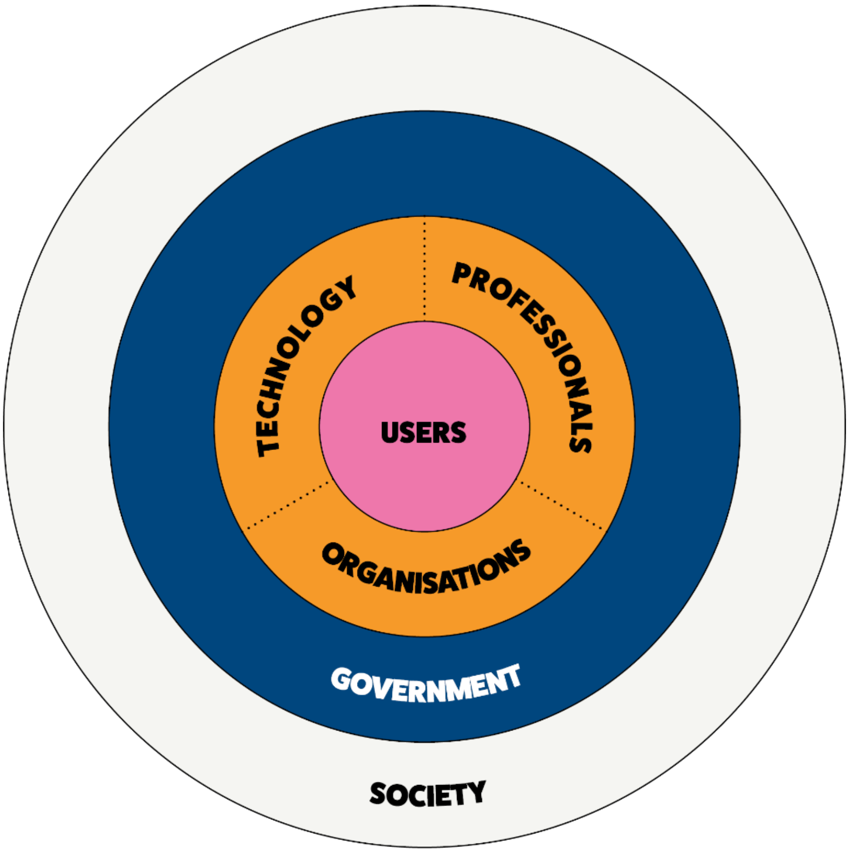

How do you use this quality framework?

The framework with the three pillars of technology, organisations and professionals is your starting point for looking at the quality of your digital care, assistance and support. It is an overview of the minimum and optional criteria. To get started with the framework in concrete terms, you can use the self-assessment tool (9). It would also be possible to create derivatives applied to a specific context, such as the sector, the type of organization, the organizational culture, the professional or the user.

Minimum quality criteria and optional criteria

Minimum quality criteria within each pillar are essential to take into account. Within the various pillars, the criteria are listed with bullet points and an * is placed after optional criteria.

Context

How do you decide which optional criteria to apply?

These vary depending on the technology, the professional and the user and what they use the technology for. To do this, you can look at the interactivity and risks of your digital offer, for example:

Example: how does interactivity play a role?

- Do users only get information through the technology? Then they do not share any personal information. They are therefore less likely to violate privacy. But it is precisely here that it can be important to offer extra help in case of shortcomings of the technology. For example, supporting elements so that people who are less proficient in language also understand the content of the technology.
- Are users actively using the technology, and sharing more personal information? Then additional criteria are needed to guarantee privacy.
- Is there interaction between the users and professionals? Or between users? Then the criteria for the competencies of the professionals and safety are more important.

Example: how do possible risks play a role?

- Are there few risks when using the digital offer? For example, because no data is shared and there is no vulnerable target group, fewer criteria will have to be used.
- Does use still pose some risks, for example in terms of interpretation or privacy? Then specific criteria around these risks will play an important role.
- Is there a high-risk digital offering? This applies, for example, to vulnerable target groups or digital care, assistance and support with a major impact. In this case, many criteria will be seen as important.

Other factors to consider are vulnerability and autonomy. Are users unable to access their network? Are they less independent? Then certain criteria are of greater importance. Think of support from the organization, competencies of a professional or data security.

Pillar technology

The following criteria mainly relate to the technology used in digital care, assistance and support. These criteria build on the ISO standard ISO/TS 82304-2 (15) with adjustments from the research (see final report; 9). The pillar contains the following themes:

- Technology provider: where criteria relating to the accessibility and reliability of the technology provider are discussed in more detail.
- The aim of the technology: where criteria around the general regulations, benefits and support and contact options are discussed in more detail.
- Ease of use: where criteria around accessibility and user-friendliness are discussed in more detail.
- Deontology: where criteria relating to risks, ethical issues and safety are discussed in more detail.
- Robust construction of the technology: where criteria relating to the (safe) construction of the technology and interoperability are discussed in more detail, so that technologies can be safely linked to each other.

Provider technology

- The provider of the technology is easy to find.
- There is a helpdesk; This is easily approachable and easily accessible, regardless of whether the provider itself or another partner offers the helpdesk (2; 9; 33).

Purpose of the technology

Requirements

- It is clear
  - which users or professionals the technology is intended for
  - for or up to what age the technology is intended
  - what the other limitations are for users or professionals
  - what the purpose of the technology is
  - how to use it
- The technology is in accordance with the medical device regulation, where applicable (Box 3.2.1.1).
- If artificial intelligence is used in the technology, the technology is in accordance with the applicable regulations (including European AI legislation (Box 3.2.1.2)).
- Appropriate scientific substantiation has been used in the development of the technology.
- Professionals participated in the development of the technology.*

Box 3.2.1.1: What does the law say about medical devices?

Europe

The Medical Device Act sets requirements for quality and safety to protect users. The manufacturer must verify that the use and purpose of the technology meet the definition of a medical device. This must be done in each country according to the laws of the country where the technology becomes available. (31)

Belgium

In Belgium, the Federal Agency for Medicines and Health Products (FAGG) is responsible for the laws and supervision of medical devices. (10)

<u>Box 3.2.1.2: European AI legislation</u>

The European law on AI sets requirements for the development and use of artificial intelligence within the European Union. The goal is to make the AI systems safe, ethical and transparent by aligning the rules with the risks. This means that systems with unacceptable risks are banned, and that other systems must meet strict requirements. (28)

Added value

- Users receive tailor-made information about the interventions and techniques used.
- Users are made aware of all efforts (including financial costs and time investments) they need to make to obtain the added value.
- The information in the technology (e.g. recommendations for the user) is systematically updated. The sources of this information are disclosed to the users.
- A description is available that explains the added value of using the technology, both for users and for professionals*
- There is evidence that shows that there is a benefit to using the technology. (Box 3.2.2.1).*
- Users and professionals are provided with information about who is contributing financially to the development and maintenance of the technology.*

<u>Box 3.2.2.1: Evidence-Based Practice and Practice-Based Evidence</u>

Evidence-Based Practice (EBP) and Practice-Based Evidence (PBE) complement each other.

EBP starts from scientific evidence. Professionals make decisions based on that evidence, supplemented by their own expertise and the user's preferences. EBP provides a good basis for testing and assessing technologies in controlled research.

PBE starts from what works in practice, by collecting data from daily practice. In that context, there is often more variation and complexity than in controlled studies. PBE shows how technologies are used in practice, including interaction with users and professionals.

Support and contact

- Users get <u>support</u> from a professional when necessary. (9)
- It is clear who, when, and how users can take contact. For example, whether they get an automatic answer, have contact with a professional or with peers. (3)
- It is clear who the users can contact in case of a crisis or if they need urgent help. (5)

Convenience

Accessibility

- The technology complies with the accessibility principles of the European Accessibility Act (EAA) (Box 3.3.1.1).

<u>Box 3.3.1.1: EAA</u>

The aim of these laws is to make products and services within the EU more accessible to everyone, with a special focus on people with disabilities. The law imposes specific requirements on digital devices so that they are more accessible and so that more people can use them independently. (6)

Usability

- The design of the technology is based on understanding of users and professionals, their tasks and their environment.
  - It is clear who the users and professionals are.
  - To improve ease of use, how and where someone uses the product or service is taken into account. For example:
    - whether the app or the device is easy to operate
    - whether it helps to support desired behaviour
    - whether it works on the available devices
    - whether there is access to wifi and electricity.
  - The users and professionals are involved in the entire design and development, with specific attention to the most vulnerable.
  - The technology is constantly being improved by the evaluation of and by the users and professionals.
- There are measures in place to prevent errors and misuse of the technology.
- Users and professionals receive sufficient information about the technology before purchasing or installing it. For example
  - The most important features
  - How their personal data is used
  - how much it costs and how they have to pay
  - in which languages they can use the technology
  - Manufacturer Information
  - the date of the last update
- The technology is intuitive to use. If not, a user manual is available. Suitable additional resources are available to help users and professionals who experience problems with the technology. (9)
- The technology is regularly improved and updated based on user experience and usability.
- The technology does not use 'dark patterns' to mislead or pressure users and professionals. These are techniques that ensure, for example, that users click on things that are not in their favor and of which they are not aware. For example, sharing data without being asked or taking out a subscription. Or if a technology sets adverse options as the default ('opt-out' instead of 'opt-in'), hides important information or makes it difficult to opt out.

Deontology

Risks

- The risks of the technology have been analysed and measures have been taken to manage these risks.
- If necessary, it is clear that the approval of a professional is required to use the technology.
- Users and professionals are provided with information about the risks, reasons for not using the technology and the limitations of using the technology.

- Safety incidents when using the technology are collected and assessed (see Box 3.4.1.1).

Box 3.4.1.1: Security incidents

The law on medical devices, which also includes health apps, refers to 'adverse event detection'. Possible incidents include incorrect data transfer, incorrect display of health information, technical failures during online consultations, or bugs in an app that lead to erroneous self-reporting or interpretation. Registration can include recording the nature of the incident, the impact on the professionals and user, the frequency, the time and the technology used. This data forms the basis for assessing the problem and, if necessary, adjusting it or reporting it to the competent authorities.

Ethics

- Ethical questions are mapped out and assessed with the users and professionals.
- Advertisements are discouraged in technology. If this is part of the technology, users and professionals will receive information about it. It is clear what advertisements are and what belongs to the digital offer.
- It is recommended to have the technology approved by an ethics committee or advisor (Box 3.4.2.1).*

Box 3.4.2.1: Ethics Committees

You can make ethical choices yourself. But it is recommended to call on an ethical advisor, even if it is not required by law. Several university and general hospitals therefore have their own ethics committee.
Sometimes advice from an ethics committee is mandatory. Does your target audience consist of patients? In that case, the Law on experiments applies to human beings in Belgium (39), even if it is not an experiment in the narrow sense of the word. For example, do you want to survey patients? Then approval by a recognized ethics committee is mandatory

Safety

In terms of personally identifiable information (PII):

- If the technology collects personal data, it must comply with the General Data Protection Regulation (30). This means that the privacy of the users is protected. Organizations must be open about how they use data, and users have rights over this data.
- The technology transmits and stores PII in a secure manner, with strong encryption.

In terms of development:

- The security of information has been included in the technology from the start of development (secure by design). (15)
- A secure encryption standard is followed.
    - Use only safe features.
    - Use the right version of programs to convert codes and secure tools to build a program.
    - Handle input and other data securely and with restrictions.
    - Use tools to identify security issues early on.
    - Create a plan for dealing with errors in technology. (15)

- The technology is in accordance with the European Health Data Space. This is legislation to be able to share information about health between patients, healthcare providers and researchers, with respect for privacy. (8)
- The technology complies with the European Cyber Resilience Regulation. This sets requirements for the security of all physical digital products and software connected to the internet or other networks sold in the EU. (29)
- The manufacturer follows the laws that protect the sensitive information against security breaches: it is in accordance with the NIS2 legislation (Box 3.4.3.4).
- The information security policy is easily accessible to users and professionals.

In terms of access:

- There is a process in place to prevent unauthorized access to and modifications to the source code of the technology e.g. Multi-Factor Authentication (25)
- Only the right people are allowed access. Access to the technology is secured with
  - User authentication, which checks that someone is really who they say they are.
  - Authorization, which determines what a person is allowed to do. If users or professionals log in, they only have access to the functions they need. The external users cannot modify the application.

In terms of maintenance:

- External information, programs, or components created by others for the technology are monitored, maintained, and secure.
- Security is tested regularly, at least in the event of major changes.
- If there are security issues, they are reported, assessed, recorded, and resolved quickly.

Box 3.4.3.4: NIS2 legislation (cybersecurity of network and information systems of general interest for public safety)
This is Belgian legislation (continued from a European directive) that strengthens cybersecurity in the world. It is intended for healthcare, financial services and digital infrastructure, among other things. They must take measures for their specific risks. In addition, there are rules for reporting cyber incidents, so that they can be resolved quickly. (1)

Robust construction of the technology

Technical robustness

- The technology works as intended. (33)
- There is information on all the technical requirements for the use of the technology.
- The principles of open-source software are recommended. Access to information, collaboration and the adoption of common goals ensure faster and better solutions (9).
- A configuration management plan has been drawn up for the technology. This plan ensures that the technology continues to work. And that problems during use are solved as quickly as possible. (15)

- There is a validation and verification plan for the technology. These are about the tests that are needed if something changes in the technology or in the system. The validation shows that the technology does what it is for. The verification shows that it is working as planned. (15)
- A release and deployment process has been established. This ensures that updates are carried out without any problems.
- Technology must continue to function reliably and adapt as needs change. This requires a good maintenance process (Box 3.5.1.1.).
- The software system has been tested (Box 3.5.1.2.)
    - The navigation to all parts of the technology have been tested.
    - The technology has been tested on all software platforms on which it can be used.
    - The technology has been tested on the most common devices of users and professionals.
    - The cooperation with other software has been tested.
    - The technology is tested again for each new version.
    - If there are any problems, the previous version can be used again.
    - Users and professionals can report technical errors. (35)
- The technology can handle a significant increase or spike in demand.*

<u>Box 3.5.1.1: Maintenance process technology</u>

Technology must continue to function reliably and adapt as needs change. This requires a good maintenance process. There are four types, each with a specific focus:

- *Corrective maintenance fixes errors or bugs experienced by users or professionals, and keeps the technology working properly.*
- *Preventive maintenance should prevent future problems. It keeps the systems up-to-date and detects errors before they occur.*
- *Adaptive maintenance ensures that software continues to work with new standards, technologies or laws and regulations.*
- *Perfective maintenance improves the software, so that it is easier to use or maintain, or works better, for example.*

(9)

<u>Box 3.5.1.2: Test-driven development</u>

In test-driven development, tests are written before developing software. This ensures fewer errors, and a code that is reliable and easier to maintain. Manual and automatic tests are required:

- *Manual testing looks at how the software performs in different scenarios. This is important to evaluate the experience of users and professionals, visual aspects and complex interactions.*
- *Automatic testing is done by scripts or tools that are pre-set to check specific features. They are indispensable for repeated and large-scale checks.*

(9)

Interoperability

- The technology must be able to connect to other (existing) services and to other technologies. (9) (Box 3.5.2.1).*

<u>Box 3.5.2.1: Extending interoperability</u>

- *There is understandable and detailed information about how the technology works. With instructions on how to work with other software e.g. APIs.*
- *There are documents with explanations and instructions about the terms used.*
- *The technology checks whether the transfer of data is done correctly.*
- *Users can get their personal information if they want to transfer it to another platform.*
- *Every technology should follow the FAIR principles. In that case, data is*
    - *Findable*
    - *Accessible*
    - *Interoperable*
    - *Reusable*

  Data is structured in such a way that it can later be integrated into broader systems, such as the Belgian Integrated Health Record.

- *If a technology links to health safes, it must comply with Fast Healthcare Interoperability Resources (FHIR) standards: These make it possible to exchange information about health securely, for all those involved in healthcare, such as healthcare providers, patients, payers, researchers.*

(23 &7)

Pillar organizations

The following criteria relate to the organisations that offer digital care, assistance and support. These criteria are based on the 'Organisational Aspects' from the Health Technology Assessment Core model (18) and adapted from the research.

The pillar focuses on the different levels of the Welfare, Public Health and Family policy area (micro, meso and macro). Do you look at the criteria from a specific perspective? Then that gives different answers. Therefore, keep one level in mind, e.g. your organization, a partnership or umbrella organization or an agency. For a certain application of technology, you can then go over these criteria several times.

The Organisations pillar includes criteria in the field of the following themes:

- Providing digital care, assistance and support: where we go deeper into how technology affects operations, what path users take, who is involved, how professionals can be supported and how quality can be guaranteed.
- Cost of the process: this takes into account the costs of hardware and software, changing needs and impact of the technology.
- Management: where the necessary vision is further framed.
- Culture: here you can find criteria for acceptance of technology.

Providing digital care, assistance and support

- The users and their needs are always at the heart of your digital care, help and support.
    - This also applies to people who cannot or do not want to be helped digitally.
    - That is why the organization must be accessible through multiple channels (click-call-connect principle): digitally, by phone, face to face and so on. (20)
- The use of the technology is in line with the mission and vision of the organization.

Technology affects the operation of the organization

- The organisation describes the influence of technology on the operation and guarantees the continuity of the assistance and services, inside and outside the organisation. There is an overview of the current tasks, functions and work processes, with the technology that is used.
- Attention is paid to the changes that a technology entails. For example, no or fewer face-to-face activities or more support in the home environment.
- The organization makes an overview of all parties involved in the digital work processes, both inside and outside the organization. A good example is the care pathway for patients with chronic heart failure (Box 4.1.1.1).*
- The different steps of the process are well aligned so that continuity can be ensured. *
- The organization ensures that the digital offer complements the online and offline offer already used, and does not replace it.*

<u>Box 4.1.1.1: Apps in care pathways</u>

The care pathway for patients with chronic heart failure is monitored via an app. This allows care providers to analyze patient data, intervene quickly and communicate with each other.

- *Cardiologists and specialist nurses within hospitals form 'telemonitoring teams' within an organisation (at the intra-organisational level).*
- *These teams work closely with general practitioners, who manage the patient's global medical file. In this way, they guarantee care (at an inter-organisational level).*
- *The National Institute for Health and Disability Insurance (NIHDI) has approved an agreement that will reimburse hospitals from January 2025 for the remote monitoring of patients with chronic heart failure (at the policy level). (32)*

User Path

The organization has insight into how the users can use the technology:

- The organization supports and monitors the users so that they can use the technology and achieve their predetermined goal. (13)
- The organization makes an overview of the steps that the users take to achieve their goal. Think of waiting times, the duration of an online session, the preparations that the users have to make. This can be done, for example, with client journeys (Box 4.1.2.1) or live testing. The organization pays extra attention to vulnerable users, children and young people.*

.

<u>Box 4.1.2.1: Client journeys</u>

Client journeys examine the needs of the users and make clear the steps that clients take in the digital offer. Professionals and users look at the desirability, added value, urgency and feasibility of digital care, assistance and support from the perspective of the users. (26)

Engage users, professionals, and key others

- Technology has an impact on everyone involved in care, assistance and support. Therefore, from the outset, the organization supports and involves users, professionals and key others in the adoption of the technology, for example through a <u>Change Management</u> route.

Training and supporting professionals

- Professionals receive training and support in adopting the technology. As a result, professionals are given new tasks or new positions are attracted, such as a digicoach or digital navigator. The organization has a budget and time for tailor-made support.
- The organization supports professionals as their work changes due to technology. She also pays attention to satisfaction when professionals use the technology.*

Guaranteeing and controlling quality with a system

- There is a system that monitors quality at various levels. There are standards and indicators that indicate when there is a deviation from that standard.*
- How that system is introduced can vary, but there is at least one coordinator and follow-up.*
- The organisation also follows up on the other (inter)national, regional or (inter)organisational quality standards, monitoring and registration, for example ISO 9001 and NEN-7510 (Box 4.1.6.1).*

Box 4.1.6.1: ISO 9001 and NEN-7510

To monitor quality in the organization, you can use different quality systems. For example, the ISO 9001 (14) for quality management systems and NEN-7510 series for information security in healthcare (24). This is mandatory in the Netherlands, and is based, among other things, on ISO/IEC 27001 and ISO/IEC 27002.

But beware. Quality systems are a tool, not a guarantee that the risk has actually been reduced to a reasonable level.

Cost of the process

Cost of purchasing, installing, and maintaining the technology

The organization prepares for all investments at the different stages of the process:

- the costs and profits of long-term maintenance and support. (19) More specifically:
    - cost of the hardware and software of the technology itself
    - costs of technical staff who provide analysis, development, maintenance and support
    - cost of training and the time it takes
    - costs to keep the system running such as server costs, upgrade costs
    - gains from, for example, more efficient operation
- the cost of hardware for professionals and users, such as headphones, smartphones
- the costs for the client: the offer must be affordable and does not form a barrier for the client. (13)

Impact of technology on needs

- The organization checks whether other needs arise from the user or professional as a result of the technology. For example, is less intensive supply needed? Does the technology impact the workload? The organization supports the professionals, users and relatives, if necessary.

Impact of the technology on the budget

- The organization evaluates the impact of the technology on the available finances and budgets of government, organizations and users.*
- The organization evaluates these financial consequences at different times.*

Management

The management of the organization has a vision on digital care, assistance and support (9):

- the management of resources, such as investments
- the coordination of the support, the different levels, the steps and other parties in the process
- The objectives
- check
- safety
- Evaluation and sanctions

Adoption of technology

- The organization examines the acceptance of the organization, professionals and users of the technology. If necessary, the organization ensures greater support.

Pillar of professionals

The following criteria relate to the specific competencies that professionals, workforce and volunteers need for digital care, assistance and support. These criteria are based on the digital competencies of the social-agogic professional (4) and adapted from the research (see final report; 9).

The competencies are a shared responsibility of professionals, organizations and governments. Organisations and governments contribute by providing the necessary support.

This pillar comprises three clusters

- Promoting digital inclusion and digital competences: in which attention is paid to the inclusion and competences of both the user and those of the professional.
- Digital care, assistance and support to users and their network: this goes into more detail about consciously selecting, offering properly and managing safely.
- Helping to shape digital care, assistance and support in the organization: where criteria are given to drawing up a vision and thinking constructively in the organization.

In addition, professionals have the general competencies necessary to exercise their profession. (9) These are described in the competency profiles and professional qualifications (38,40). Think of:

- adhere to codes of ethics
- acting ethically and safely
- professional communication
- building relationships with clients
- paying attention to cultural diversity
- apply reliable methods

Promoting digital inclusion and competences

Promoting digital inclusion

The professionals:

- recognise that users and their network may need support in digitisation.
- identify and discuss digital exclusion. They can assess whether users can safely participate digitally and whether they need support in doing so. (11)
- help to strengthen the digital competencies of the users and their network or refer them to their network or other organizations for this purpose.
- make an overview of the digital possibilities and the Media literacy of users. They adjust their trajectory accordingly (Box 5.1.1.1).
- are aware of the importance of digital inclusion (Box 5.1.1.2).*
- can assess the level of digital inclusion among users.*
- contribute to the digital inclusion of the users themselves or refer them to their network or other organisations.*

Box 5.1.1.1: Checklists Ready to blend and Fit for blended care

These are checklists for social workers; Checklist Ready to Blend (36) and mental health care workers; fit for blended care (17). They help assess whether a client has the necessary tools and competencies to receive digital guidance and what support the client needs to be eligible for this.

Box 5.1.1.2: Digital inclusion

Digital inclusion is ensuring that all users can participate in the digital society. It is a prerequisite for working digitally.
Digital inclusion is about:

- *Do the users have access to the technology?*
- *Is there practical support and information for digital questions and problems?*
- *Do users have enough digital skills to use the technology?*
- *Doesn't the technology have extra barriers?*

(21)

Strengthening their own digital competencies

The professionals:

- pay attention to their own media literacy and digital competences.
- are aware of the possible consequences of digital communication with the user, such as via social media.
- have the digital and media literacy competencies that are relevant to their work.
- make informed choices about privacy, legislative and ethical issues.
- have the knowledge and skills to apply their deontology in digital care, assistance and support
- find, assess and process digital information smoothly.*
- continue to learn and improve their own digital competences.*

Providing digital care, assistance and support

Consciously selecting technology

The professionals:

- Consider the purpose of the technology, the network, and the capabilities of the users when choosing a technology.
- involve users in choices about technology.
- pay attention to situations in which the digital offer is no longer sufficient or no longer works properly. They provide alternatives.
- are open to the technology that users are already using.*

Providing digital care, assitance and support to the users and their network

The professionals:

- can use technology in a targeted and systematic way.
- can build a professional relationship digitally. (27)

- can communicate digitally clearly and inclusively. They can find, select, reformulate or create general and personal digital information about and for the users.*
- use technology that supports the content of the offer, from intake or diagnosis to evaluation and aftercare (27, 40).*
- find relevant and good technological applications and can use them in the offer.*
- have a positive critical ambassador attitude about the use of technology and its limits. They focus on the added value of technology for the user.*

Securely manage digital client data

The professionals:

- understand the importance of securely sharing digital data about and with users.
- can consult, analyse, interpret and enter user data in a digital file.
- Teach the users how to work with their file, if that is part of the job.
- can handle the sharing of data from the user's file in a deontologically sound way.
- reflect about the purpose and use of sharing digital data through technology.*

Helping to shape digital care, assistance and support in the organization

A vision on digital care, assistance and support

The professionals:

- help shape the vision on digital care, assistance and support in the organization.*
- help strengthen the confidence of other professionals and users in the digital offer (Box 5.3.1.1).*
- help find solutions to problems with the digital offer.*

Box 5.3.1.1: survey of employee confidence in online help

This questionnaire gives you an overview of employees' confidence in digital working.
Think of:

- *the confidence that the digital offer is an added value*
- *confidence that the effort to learn how to use the digital offer is worthwhile*
- *the feeling about the digital offer at work*
- *the trust of colleagues*
- *confidence in the technical and organisational conditions of the digital offer*
- *trust in the internet*
- *the intention to use the digital offer*
- *the confidence with which the digital offer is used*
- *the confidence that gives the organization a safe and stimulating context*

(2)

Constructively evaluating critical digital care, assistance and support

The professionals

- can constructively and critically evaluate digital care, assistance and support with sources and measuring instruments.*
- make it clear what support is needed to provide better digital care, assistance and support.*

- are interested in evolutions in digital care, assistance and support in the broad professional sector.*
- are willing and able to participate in projects that explore and develop new perspectives on digital action.*

Concepts

Understanding	Definition	First mention
Policy area Welfare, Public Health and Family	The policy area of Welfare, Public Health and Family includes the Flemish Ministry of Welfare, Public Health and Family, which includes the Department of Care and Growing Up, the Flemish Agency for Persons with Disabilities, the Flemish Agency for the Cooperation on Data Sharing between Actors in Care, the Public Psychiatric Care Centre Geel, the Public Psychiatric Care Centre Rekem and the Agency for Payment of the Growth Package. (37)	Back to first entry
Change management	Change management refers to the actions a company takes to change or adapt an important part of the organization. This can include company culture, internal processes, underlying technology or infrastructure, company hierarchy, or any other critical aspect. (12)	Back to first entry
Digital skills	Digital skills and media literacy mean that the user has sufficient competencies to use or deploy online help. (9)	Back to first entry
Ethics	Ethics is thinking about how one can act morally correctly in a specific situation, with attention to values and relationships. As a result, ethics is not a set of fixed obligations, but a process of consciously dealing with what is good in a specific context. In digital care, assistance and support, ethics therefore means the conscious use of technology in a way that respects the values of the profession. (34)	Back to first entry
Society	Society encompasses the broader changing needs, technological progress, new digital possibilities and evolutions in society both within and outside the policy area of Welfare, Public Health and Family.	Back to first entry
Media literacy	"If you are media literate, you can use and understand media. You need certain knowledge (knowing), skills (ability) and attitudes (wanting, daring) for that. So it's not just about technical button knowledge: looking at digital problems in a solution-oriented way, creating media content yourself and dealing critically and consciously with media, for example, are also important." (22)	Back to first entry
Support	Support means that when using online help, it is easy to call on staff or your own support network. On the one hand, the accessibility of content staff can offer added value for confidence in the tool, such as starting procedures in crisis situations. For the professionals, inter- or supervision can also offer substantive enrichment. On the other hand, a helpdesk can serve as technical support, both for professionals and for those who are the recipients of online help. (9)	Back to first entry
Organization	This refers to the organisations in the policy area of Welfare, Public Health and Family in which digital care, assistance and support will be deployed, possibly under the guidance of professionals (professionals and volunteers) active within this organisation.	Back to first entry
Government	Government stands for regulations and policy choices at different levels, European, Belgian, Flemish and local.	Back to first entry

Pillar	A pillar in this quality framework indicates one of the 3 cores of the framework, namely technology, professional or organisation. It was decided to take this as the core because these are the three aspects that a government has a grip on and can therefore impose quality criteria.	Back to first entry
Professional	Professionals are professionals or volunteers who are active within the domain. The competencies of professionals treated in this context have been specifically applied to the use of digital care, assistance and support.	Back to first entry
Technology	Technology because all online care, assistance and support tools, whether or not under the guidance of a professional or in an organization. It does not refer to any particular type or category of technology, i.e. apps, websites, chat, email, video calling, AI, Virtual Reality, Augmented Reality, wearables, etc. included.	Back to first entry
Vision	Vision means that the choice for online help must be made from a clear idea of why it is appropriate to opt for online help and that this is clear to and supported by all actors involved. That vision then takes shape in an implementation plan. This takes into account all essential requirements and framework conditions - for all actors involved - for successful online help. The plan also includes an estimate of the investment required (time, infrastructure, budgets, business plan and cost-effectiveness) for a successful implementation to take place. (9)	Back to first entry

References

1. België. Wet van 26 april 2024 houdende vaststelling van een kader voor de cyberbeveiliging van netwerk- en informatiesystemen van algemeen belang voor de openbare veiligheid (NIS2-wet). Belgisch Staatsblad. 2024. http://www.ejustice.just.fgov.be/eli/wet/2024/04/26/2024202344/justel
2. Bocklandt P. Medewerkersvertrouwen in onlinehulp. 2020. https://cdn.nimbu.io/s/0hkvgjb/channelentries/ywob7v1/files/medewerkersvertrouwen%20in%20onlinehulp%20-%208%20januari%202020.pdf?moerema=&dl=1
3. Bocklandt P, Beelen S, Claeys H, Custers S, Mindermann O, Daele T. Onlinehulp-Vlaanderen. Screeningscriteria voor apps en websites in onlinehulp-apps.be. 2022. https://www.onlinehulp-apps.be/screeningskader
4. Bocklandt P, Verplancke J, Faelens L, et al. Een kader voor digitale competenties van sociaal-agogische professionals. 2025. https://cdn.nimbu.io/s/0hkvgjb/assets/1737548451710/20250122_p_een-kader-voor-digitale-competenties-van-sociale-professionals.pdf
5. De Jaegere E. Ethische richtlijnen voor 'technology-based' suïcidepreventie programma's. https://www.flanderscare.be/sites/default/files/media/ethiek_FCCOH.pdf
6. European Union. Directive (EU) 2019/882 of the European Parliament and of the Council of 17 April 2019 on the accessibility requirements for products and services (European Accessibility Act). Official Journal of the European Union. 2019;L151:70–115. Available at: http://data.europa.eu/eli/dir/2019/882/oj. Accessed November 25, 2025.
7. Federale Overheidsdienst Volksgezondheid. Actieplan e-Gezondheid 2022-2024. 2022. https://www.health.belgium.be/sites/default/files/uploads/fields/fpshealth_theme_file/actieplan_egezondheid_2022-2024_protocolakkoord.pdf
8. European Union. Regulation (EU) 2025/327 of the European Parliament and of the Council of 11 February 2025 on the European Health Data Space and amending Directive 2011/24/EU and Regulation (EU) 2024/2847. Off J Eur Union. 2025;L5:1-96. https://eur-lex.europa.eu/eli/reg/2025/327/oj
9. Delphi expert survey, focus groups and interviews. (2024) *Final Report*. www.steunpuntwvg.be/publicaties
10. Federaal Agentschap voor Geneesmiddelen en Gezondheidsproducten (FAGG). Medische hulpmiddelen en hun hulpstukken. FAGG. 2025. https://www.fagg.be/nl/MENSELIJK_gebruik/gezondheidsproducten/medische_hulpmiddelen_hulpstukken
11. Hartman-van der Laan, M. Sociaal werk in de digitale samenleving. Coutinho. 2019. https://www.coutinho.nl/nl/sociaal-werk-in-de-digitale-samenleving-9789046906590
12. Harvard Business School Online. 5 steps in the change management process. HBS Online Business Insights Blog. 2020. https://online.hbs.edu/blog/post/change-management-process
13. Inspectie Gezondheidszorg en Jeugd. Toetsingskader Digitale Zorg – uitgebreide versie. Ministerie van Volksgezondheid, Welzijn en Sport. 2024. https://www.igj.nl/publicaties/toetsingskaders/2024/05/06/toetsingskader-digitale-zorg-uitgebreide-versie
14. International Organization for Standardization. ISO 9001:2015 – Quality management systems – Requirements (5th ed.). ISO. 2015. https://www.iso.org/standard/62085.html

15. International Organization for Standardization. ISO/TS 82304-2:2021 – Health software – Part 2: Health and wellness apps – Quality and reliability (1st ed.). ISO. 2021. https://www.iso.org/standard/78182.html
16. International Organization for Standardization & International Electrotechnical Commission. ISO/IEC 27001:2022 – Information security, cybersecurity and privacy protection – Information security management systems – Requirements. ISO/IEC. 2022. https://www.iso.org/standard/27001.html
17. Kip H, Wentzel J, Kelders SM. Shaping Blended Care: Adapting an Instrument to Support Therapists in Using eMental Health. JMIR Mental Health. 2020;7(11). doi:10.2196/24245
18. Kristensen FB, Lampe K, Wild C, Cerbo M, Goettsch W, Becla L. The HTA Core Model ® —10 Years of Developing an International Framework to Share Multidimensional Value Assessment. Value in Health. 2017;20(2):244-250. doi:10.1016/j.jval.2016.12.010
19. McGrath P, Wozney L, Rathore SS, Notarianni M, Schellenberg M. Toolkit for E-Mental Health Implementation. Mental Health Commission of Canada; 2018. https://www.mentalhealthcommission.ca/professional-resources/ementalhealth/toolkit-for-e-mental-health-implementation/
20. Mediawijs. Aan de slag als digihelper. 2022. https://assets.mediawijs.be/2022-06/mediawijs_digitaleinclusie_nl.pdf.
21. Mediawijs. Digitale inclusie. 2023. https://www.mediawijs.be/nl/artikels/wat-digitale-inclusie
22. Mediawijs. Wanneer ben je mediawijs? 2024. https://www.mediawijs.be/nl/mediawijsheid
23. MedTech Europe & COCIR. Interoperability standards in digital health: A white paper from the medical technology industry. 2021. https://www.medtecheurope.org/wp-content/uploads/2021/10/mte_interoperability_digital_health_white-paper_06oct21.pdf
24. Stichting Koninklijk Nederlands Normalisatie-instituut (NEN). NEN 7510:2017 – Informatiebeveiliging in de zorg – Eisen voor een managementsysteem (3e ed.). NEN. 2017. https://www.nen.nl/nen-7510-1-2017-nl-232492
25. N.H.S. Digital Technology Assessment Criteria (DTAC). UK. 2021. https://transform.england.nhs.uk/key-tools-and-info/digital-technology-assessment-criteria-dtac/
26. Pattyn E, Bocklandt P. Cliëntreizen rond onlinehulp: achtergrond en aanpak. 2020. https://cdn.nimbu.io/s/0hkvgjb/channelentries/un0fsc3/files/Cli_ntreizen%20-%20achtergrond%20en%20aanpak%20-%20versie%2010%20februari%202020.pdf.
27. Pote H, Moulton-Perkins A, Holloway-Biddle C. Competence framework for digital clinical practice: Psychological practitioners. British Psychological Society, Division of Clinical Psychology, Digital Healthcare Committee. Published online 2020. https://digitalhealthskills.com/digitalcompetencies
28. European Union. Regulation (EU) 2024/1689 of the European Parliament and of the Council of 13 June 2024 laying down harmonised rules on artificial intelligence. Official Journal of the European Union.2024; 1689, 1–144. http://data.europa.eu/eli/reg/2024/1689/oj
29. European Union. Regulation (EU) 2024/2847 of the European Parliament and of the Council of 23 October 2024 on horizontal cybersecurity requirements for products with digital elements and amending Regulations (EU) No 168/2013 and (EU) No 2019/1020 and Directive (EU) 2020/1828 (Cyber Resilience Act) [Regulation]. Official Journal of the European Union. 2024; L 2847, 1–66. http://data.europa.eu/eli/reg/2024/2847/oj

30. European Union. Regulation (EU) 2016/679 of the European Parliament and of the Council of 27 April 2016 on the protection of natural persons with regard to the processing of personal data and on the free movement of such data, and repealing Directive 95/46/EC (General Data Protection Regulation) [Regulation]. Official Journal of the European Union. 2016; L 119, 1–88. https://eur-lex.europa.eu/eli/reg/2016/679/oj
31. European Union. Regulation (EU) 2017/745 of the European Parliament and of the Council of 5 April 2017 on medical devices, amending Directive 2001/83/EC, Regulation (EC) No 178/2002 and Regulation (EC) No 1223/2009, and repealing Council Directives 90/385/EEC and 93/42/EEC [Regulation]. Official Journal of the European Union. 2017; L 117, 1–175. https://eur-lex.europa.eu/legal-content/EN/TXT/?uri=CELEX%3A32017R0745
32. Rijksinstituut voor ziekte- en invaliditeitsverzekering (RIZIV). Zorg op afstand: telemonitoring en therapiebegeleiding bij chronisch hartfalen. RIZIV; 2024. https://www.riziv.fgov.be/nl/professionals/verzorgingsinstellingen-en-diensten/ziekenhuizen/verzorging-in-ziekenhuizen/zorg-op-afstand-telemonitoring-en-therapiebegeleiding-bij-chronisch-hartfalen
33. Schalken F, Obyn C, Vinck I, Meester C, Jespers V, Pouppe C. Handboek online hulpverlening. Bohn Stafleu van Loghum. 2013. https://www.bohnstafleuvanloghum.nl/product/handboek-online-hulpverlening
34. Tirions M, Raeymaeckers P, Boxstaens J, Cornille A, Gibens S, Postma Y. #sociaalwerk. Leuven: Acco; 2019.
35. Van Gucht K. Guidelines for ehealth applications. imec; 2021.
36. Verplancke J, Bocklandt P. Ready to blend?! Is jouw cliënt klaar voor een blended traject? Checklist voor sociale professionals en cliënten om samen blended te werken. Arteveldehogeschool – Expertiselijn Mens, Samenleving & Digitalisering. 2023. https://b9e8513d6a.clvaw-cdnwnd.com/22701d8eac43dd786f280edfdcf61659/200001061-c6040c6042/Ready%20to%20blend%20-%2018%20oktober%202023.pdf
37. Vlaamse overheid. Beleidsdomein Welzijn, Volksgezondheid en Gezin. Vlaamse overheid. https://www.vlaanderen.be/organisaties/administratieve-diensten-van-de-vlaamse-overheid/beleidsdomein-welzijn-volksgezondheid-en-gezin
38. Schotte C, Broeck N. De competenties van de Belgische klinisch psycholoog: integratie van het profiel in Advies. van de Hoge Gezondheidsraad en het CanMEDS-model VVKP. 2018;(9194). https://vvkp.be/sites/default/files/TKP%202018-01_04_Competenties.pdf
39. België. Wet van 7 mei 2004 inzake experimenten op de menselijke persoon. Belgisch Staatsblad; 2004. https://etaamb.openjustice.be/nl/wet-van-07-mei-2004_n2004022376.html
40. Zhu H, Andersen ST. Digital competence in social work practice and education: experiences from Norway. Nordic Social Work Research. 2021;12(5):823-838. doi:10.1080/2156857x.2021.1899967

Appreciatin

This framework was created with the support and input of the Department of Care, Beelen Sandra (SAM, Steunpunt Mens en Samenleving), Behets Johan (Agentschap Opgroeien), Beirens Linda (Vlaams Welzijnsverbond), Beke Eric (MPC Terbank), Bens Emmelien (De Schommel VZW), Carremans Freddy (VIVEL), Claesen Astrid (Kom op tegen Kanker), Cornelis Ester (Stichting Tegen Kanker), De Boeck Minne (Universitair Forensisch Centrum), De Jaegere Eva (Vlaams Expertisecentrum Suïcidepreventie, VLESP; UGent), De Wilde Joke (HOGENT 360° Zorg en Welzijn), De Wolf Ellen (CGG Andante vzw), Degryse Bart (Wit-Gele Kruis West Vlaanderen), Desmet Marieke (Digiraf), Elen Kirsten (Fara vzw), Evenepoel Tom (VAD vzw, De Druglijn), Faelens Lien (CGG Schelde Dender Waas), Goffin Tom (Metamedica, Universiteit Gent), Libin Vincent (LINC vzw), Nijs Davy, Raymaekers Anna (LUCAS KU Leuven), Roggeman Saskia (ScienceForCare, PC Sint-Jan Baptist, Zelzate), Clovis Six (BloomUp), Van den Heuvel Bavo (CRANIUM), van Leeuwen Cora (imec-SMIT, VUB), Vande Gaer Eva (Centrum ter Preventie van Zelfdoding), Vandenberghe Annelies (Ligo, Centrum voor Basiseducatie Brugge – Oostende – Westhoek), Vandenhoudt Hilde (Thomas More University of Applied Sciences), Vandeput Steven (beMedTech), Verbeke Kamiel (KU Leuven), Verspreet Sofie (OnlinePsyHulp), en de deelnemers aan de focusgroepen en interviews.

A special word of thanks also to Philippe Bocklandt (Artevelde University of Applied Sciences) who helped start the project and continued to support it later.

Citation suggestion

Buelens, F., Seymoens, T., Verplancke, J., & Van Daele, T. (2025). Quality framework for digital care, assistance and support. Centre for Welfare, Public Health and Family, Department of Care.

Contact & info

Welfare, Public Health and Family Support Centre

www.steunpuntwvg.be

swvg@kuleuven.be
