# Supplementary material for: Development of a Contextualized, Research-Based Flemish Assessment Framework for Digital Care, Assistance, and Support: Delphi Study
Source: JMIR Form Res. 2026 Apr 15;10:e88512. doi: 10.2196/88512 (PMC13129510; doi:10.2196/88512)
Supplement: Multimedia Appendix 2 [file formative_v10i1e88512_app2.pdf]

Multimedia Appendix 2: Overview of the literature

Reference		Quality criteria of technology	Regularisaton	Quality criteria of professionals	Quality criteria of users	Quality criteria of organisations	Examples of governance	Examples of reports	Social work	Mental health care	Strict criteria	Growth path to quality	Meta thinking on quality	Flanders	Belgium	Netherlands	Europe	World, applicable	World, none applicable	Interpretation/ summary
8 Caring Technology Principles. Eight guiding principles for caring technology. King Baudouin Foundation; 2021. https://www.fondsdanieldeconinck.be/en/initiative/caring-technology/	(1)	1			1	1	1			1					1					
Aaltonen ES. Client-oriented quality assessment within municipal social services. Int J Soc Welf. 1999;8(2):131-142. doi:10.1111/1468-2397.00074	(2)			1	1		1		1		1		1				1			
Abrishami P, Boer A, Horstman K. How can we assess the value of complex medical innovations in practice? Expert Review of Pharmacoeconomics & Outcomes Research. 2015;15(3):369-371. doi:10.1586/14737167.2015.1037834	(3)	1								1			1					1		
Agarwal S, LeFevre AE, Lee J, et al. Guidelines for reporting of health interventions using mobile phones: mobile health (mHealth) evidence reporting and assessment (mERA) checklist. BMJ. 2016. doi:10.1136/bmj.i1174	(4)	1								1								1		
Agentschap Digitaal Vlaanderen. Digitale dienst zelfevaluatiedocument. 2022. https://www.vlaanderen.be/digitaal-vlaanderen/vlaamse-digitale-strategie/digitale-dienstverleningsstrategie	(5)	1				1	1				1			1						
Agentschap Digitaal Vlaanderen. Scores rekenblad digitale dienst. 2022. https://www.vlaanderen.be/digitaal-vlaanderen/vlaamse-digitale-strategie/digitale-dienstverleningsstrategie	(6)	1				1	1				1			1						
Agoria en beMedTech. Validatiepiramide m-health Belgium. https://mhealthbelgium.be/nl/validatiepiramide	(7)	1	1							1					1					

Reference		Quality criteria of technology	Regularisaton	Quality criteria of professionals	Quality criteria of users	Quality criteria of organisations	Examples of governance	Examples of reports	Social work	Mental health care	Strict criteria	Growth path to quality	Meta thinking on quality	Flanders	Belgium	Netherlands	Europe	World, applicable	World, none applicable	Interpretation/ summary
Agoria en cyberstart. Cybersecurity woordenboek. https://www.agoria.be/cyberstart/cybersecurity-woordenboek?utm_campaign=%5BPCMN%5D%20CyberStart-NL&utm_medium=email&_hsmi=80113415&_hsenc=p2ANqtz-8UoQ0HzPMVlKRdo0VEaNvAJPi_89orDK9P8XuznGSkKtyn9dhKQbibRCwCOuw0VVmIr-69uPTfw1i2759GLjEkBRHG2TtcJLbiSG7OZLnrPw70yzY&utm_content=80113415&utm_source=hs_email	(8)	1				1								1						1
Agoria. Software supply chain risicobeheersing. 2023. https://www.agoria.be/cyberstart/buyers-guide-software-supply-chain-risicobeheersing?utm_campaign=%5BPCMN%5D%20CyberStart-NL&utm_medium=email&_hsmi=80113415&_hsenc=p2ANqtz-9PbjmiY7Z279uSc5HdVt9d7nFTEHp6q7hLGlosNLArTVICi6EvWTlLuZX6XqP3w4UB_nYm0Jz8C38R_ginnjvviHPYpI4XfeB6PyylVuCheRDD6a4&utm_content=80113415&utm_source=hs_email	(9)	1				1								1						1
Ahmed S, Trimmer C, Khan W, et al. A mixed methods analysis of existing assessment and evaluation tools (AETs) for mental health applications. Frontiers in Public Health. 2024;12. doi:10.3389/fpubh.2024.1196491	(10)	1								1								1		
AI4Europe. Ethic Assessment Tools. 2019. https://www.ai4europe.eu/Ethics-Assessment-Tools-ALTAI	(11)	1	1								1						1			
Almathami HKY, Win KT, Vlahu-Gjorgievska E. Barriers and facilitators of online consultations at patients' homes. JMIR Publications. 2020;22(2). doi:10.2196/16407	(12)	1	1	1	1	1		1										1		1

Reference		Quality criteria of technology	Regularisaton	Quality criteria of professionals	Quality criteria of users	Quality criteria of organisations	Examples of governance	Examples of reports	Social work	Mental health care	Strict criteria	Growth path to quality	Meta thinking on quality	Flanders	Belgium	Netherlands	Europe	World, applicable	World, none applicable	Interpretation/ summary
AMA. Validating digital health innovations. 2023. https://www.ama-assn.org/practice-management/digital/validating-digital-health-innovations	(13)	1								1									1	
APA. Health Information Technology and Telepsychology. 2016. https://www.apaservices.org/practice/legal/technology	(14)	1	1							1								1		
APA. The app evaluation model. https://www.psychiatry.org/psychiatrists/practice/mental-health-apps/the-app-evaluation-model	(15)	1								1								1		
Arteveldehogeschool. Swipe: Sterk Sociaal Werk Inspirerende Praktijken Digitalisering. 2022. https://www.arteveldehogeschool.be/nl/onderzoek/projecten/swipe-sterk-sociaal-werk-inspirerende-praktijken-digitalisering	(16)								1					1						
Artsenfederatie KNMG. Alles wat u moet weten over videoconsulten met patiënten. 2021. https://www.knmg.nl/actualiteit-opinie/nieuws/nieuwsbericht/alles-wat-u-moet-weten-over-videoconsulten-met-patienten	(17)			1												1				
ASQ. ISO 9001. 2024. https://asq.org/quality-resources/iso-9001	(18)	1																1		1
Australian Commission on Safety and Quality in Health Care. National Safety and Quality Digital Mental Health Standards. 2020. https://www.safetyandquality.gov.au/standards/national-safety-and-quality-digital-mental-health-standards	(19)	1	1																1	
Australian Government Department of Health and Aged Care. Australia: Therapeutic Goods Administration (TGA) . https://www.tga.gov.au/	(20)	1	1																1	

Reference		Quality criteria of technology	Regularisaton	Quality criteria of professionals	Quality criteria of users	Quality criteria of organisations	Examples of governance	Examples of reports	Social work	Mental health care	Strict criteria	Growth path to quality	Meta thinking on quality	Flanders	Belgium	Netherlands	Europe	World, applicable	World, none applicable	Interpretation/ summary
Australian Medical Association. National mHealth applications assessment framework. 2021. https://www.ama.com.au/sites/default/files/2021-11/AMA%20Submission%20to%20mHealth%20applications%20assessment%20framework_Final.pdf	(21)	1								1									1	
Balcombe L, Leo D. Human-Computer Interaction in Digital Mental Health. Informatics. 2022;9(1). doi:10.3390/informatics9010014	(22)	1								1								1		1
België. Wet van 26 april 2024 houdende vaststelling van een kader voor de cyberbeveiliging van netwerk- en informatiesystemen van algemeen belang voor de openbare veiligheid (NIS2-wet). Belgisch Staatsblad. 2024. http://www.ejustice.just.fgov.be/eli/wet/2024/04/26/2024202344/justel	(23)	1	1				1				1				1					
BelRAI. Wat is BelRAI - MyBelRAI? BelRAI. https://www.belrai.org/nl/wat-belrai-mybelrai-0	(24)	1		1		1	1			1					1					
Blindenzorg Licht en Liefde vzw. Toegankelijkheidsmonitor 2020. 2020. https://toegankelijkheidsmonitor.be/2020.html	(25)	1													1					
Blumenthal D. Part 1: Quality of care–what is it? The New England journal of medicine. 1996;335(12):891-894. doi:10.1056/NEJM199609193351213	(26)			1							1		1					1		
Bocklandt P, Adriaensens E, Zitter M, Sep H, Vandecasteele A. Bouwstenen Vlaams actieplan onlinehulp. Arteveldehogeschool. 2014. https://www.onlinehulp-arteveldehogeschool.be/onlinehulponderzoek/vlaams-actieplan-onlinehulp/	(27)	1	1											1						
Bocklandt P, Beelen S, Claeys H, Custers S, Mindermann O, Daele T. Onlinehulp-Vlaanderen. Screeningscriteria voor apps en websites in onlinehulp-apps.be. 2022. https://www.onlinehulp-apps.be/screeningskader	(28)	1									1			1						

Reference		Quality criteria of technology	Regularisaton	Quality criteria of professionals	Quality criteria of users	Quality criteria of organisations	Examples of governance	Examples of reports	Social work	Mental health care	Strict criteria	Growth path to quality	Meta thinking on quality	Flanders	Belgium	Netherlands	Europe	World, applicable	World, none applicable	Interpretation/ summary
Bocklandt P, Verplancke J, Faelens L, et al. Een kader voor digitale competenties van sociaal-agogische professionals. 2025. https://cdn.nimbu.io/s/0hkvgjb/assets/1737548451710/20250122_p_een-kader-voor-digitale-competenties-van-sociale-professionals.pdf	(29)			1										1						
Bocklandt P. Medewerkersvertrouwen in onlinehulp. 2020. https://cdn.nimbu.io/s/0hkvgjb/channelentries/ywob7v1/files/medewerkersvertrouwen%20in%20onlinehulp%20-%208%20januari%202020.pdf?moerema=&dl=1	(30)			1										1						
Bocklandt P. Professioneel beeldbellen in welzijnswerk, sociaal werk & geestelijke gezondheidszorg: Handvatten voor systematisch en doelgericht videochatten. Arteveldehogeschool. 2021. https://cdn.nimbu.io/s/0hkvgjb/channelentries/ulaif2b/files/methodiek%20beeldbellen%20in%20welzijnswerk%20sociaal%20werk%20en%20geestelijke%20gezondheidszorg%20-%20versie%204%20januari%202021.pdf	(31)			1		1								1						
Boutens L. Online toepassingen rond de alcohol-, drug- en gokproblematiek - inspiratie voor intermediairs. Vlaams Expertisecentrum Alcohol en Andere Drugs. 2016. https://vad.be/catalogus/online-toepassingen-rond-de-alcohol-drug-en-gokproblematiek/	(32)	1		1	1															
Boxstaens J, Reynders B. We creëren een kwaliteitskader waarin iedereen naar waarde wordt geschat, op elk niveau". 2024. https://www.zorgneticuro.be/artikel/we-creeren-een-kwaliteitskader-waarin-iedereen-naar-waarde-wordt-geschat-op-elk-niveau	(33)					1	1							1						

Reference		Quality criteria of technology	Regularisaton	Quality criteria of professionals	Quality criteria of users	Quality criteria of organisations	Examples of governance	Examples of reports	Social work	Mental health care	Strict criteria	Growth path to quality	Meta thinking on quality	Flanders	Belgium	Netherlands	Europe	World, applicable	World, none applicable	Interpretation/ summary
Braeckel L. Blended hulp reikt online en offline de hand. Welzijns- en Gezondheidsmagazine. 2019;144. doi:https://publicaties.vlaanderen.be/view-file/1472	(34)			1	1	1	1							1						
Broekhuis M, Velsen L, Hermens H. Assessing usability of eHealth technology. International Journal of Medical Informatics. 2019;128:24-31. doi:10.1016/j.ijmedinf.2019.05.001	(35)	1								1								1		
Brown P, Prest B, Miles P, Rossi V. The development of National Safety and Quality Digital Mental Health Standards. Australasian Psychiatry. 2021;30(2):154-157. doi:10.1177/10398562211042361	(36)	1																	1	
Business, Economy, Euro. CE markering. European Union. https://europa.eu/youreurope/business/product-requirements/labels-markings/ce-marking/index_nl.htm#shortcut-0	(37)	1	1							1							1			1
Byrne-Haber S. Giving a Damn about Accessibility: A Candid and Practical Handbook for Designers. UX Collective; 2021. https://www.accessibility.uxdesign.cc/	(38)	1										1						1		
Cambell A, Adams C, Montgomery RB, Cooper M, Kirkpatrick A. Web Content Accessibility Guidelines (WCAG) 2.2. W3C Recommendation. 2024. https://www.w3.org/TR/WCAG22/	(39)	1									1							1		1
CCLAB. ETSI EN 303 645 Infographics for Consumer IoT devices. 2021. https://www.cclab.com/downloads/etsi-infographics	(40)	1	1															1		
Center for Internet Security. Creating Confidence in the Connected World. 2023. https://www.cisecurity.org/	(41)																	1		
Centre for Cybersecurity Belgium. Centre for Cybersecurity Belgium. https://ccb.belgium.be/	(42)					1					1				1					

Reference		Quality criteria of technology	Regularisaton	Quality criteria of professionals	Quality criteria of users	Quality criteria of organisations	Examples of governance	Examples of reports	Social work	Mental health care	Strict criteria	Growth path to quality	Meta thinking on quality	Flanders	Belgium	Netherlands	Europe	World, applicable	World, none applicable	Interpretation/ summary
Centre for Cybersecurity Belgium. Cyber Emergency Response Team. 2023. https://ccb.belgium.be/nl/cert	(43)	1				1									1					
Centre for Cybersecurity Belgium. De NIS2-richtlijn : wat betekent dit voor mijn organisatie? 2023. https://ccb.belgium.be/nl/de-nis2-richtlijn-wat-betekent-dit-voor-mijn-organisatie#_Toc127800317	(44)	1	1												1					
Centre for Cybersecurity Belgium. Service Definition Document—Federal Cyber Emergency Response Team CERT.be. Version. 2024;4(00). https://ccb.belgium.be/cert/service-definition-document	(45)	1	1			1	1				1				1					
Connolly SL, Kuhn E, Possemato K, Torous J. Digital Clinics and Mobile Technology Implementation for Mental Health Care. Current Psychiatry Reports. 2021;23(7). doi:10.1007/s11920-021-01254-8	(46)	1		1	1	1				1								1		
Consortium WWW. Web Content Accessibility Guidelines (WCAG) 2.1. 2018. https://www.w3.org/TR/WCAG21/	(47)	1																1		
Cook L L ,Zschomler D. Virtual home visits during the COVID-19 pandemic: Social workers' perspectives. Perspectives. 2020;32(5). doi:10.1080/09503153.2020.1836142	(48)	1		1	1				1									1		
Corrêa NK, Galvão C, Santos JW, et al. Worldwide AI ethics: A review of 200 guidelines and recommendations for AI governance. Patterns. 2023;4(10). doi:10.1016/j.patter.2023.100857	(49)	1					1											1		1
Cox D. They thought they were doing good but it made people worse': why mental health apps are under scrutiny. The Guardian. 2024. doi:https://www.theguardian.com/society/2024/feb/04/they-thought-they-were-doing-good-but-it-made-people-worse-why-mental-health-apps-are-under-scrutiny	(50)	1																1		

Reference		Quality criteria of technology	Regularisaton	Quality criteria of professionals	Quality criteria of users	Quality criteria of organisations	Examples of governance	Examples of reports	Social work	Mental health care	Strict criteria	Growth path to quality	Meta thinking on quality	Flanders	Belgium	Netherlands	Europe	World, applicable	World, none applicable	Interpretation/ summary
Custers S, Drooghmans N, Nijs D. Digitale inclusie in tijden van corona. Noden uit het welzijnswerk. UCLL Research & Expertise. 2020. https://www.ucll.be/sites/default/files/documents/ucll_-_onderzoeksrapport_nodenbevraging_digitale_inclusie_in_tijden_van_.pdf	(51)	1		1	1	1			1					1						
Cwikel J, Friedmann E. E-therapy and social work practice: Benefits, barriers, and training. International Social Work. 2019;63(6):730-745. doi:10.1177/0020872819847747	(52)	1		1	1	1			1									1		
Cybersecurity Bites. Cybersecurity Bites. 2023. https://cybersecurity-bites.be/	(53)	1				1								1						1
D'Alfonso S. AI in mental health. Current Opinion in Psychology. 2020;36:112-117. doi:10.1016/j.copsyc.2020.04.005	(54)	1	1							1								1		
Daele T, Karekla M, Kassianos AP, et al. Recommendations for policy and practice of telepsychotherapy and e-mental health in Europe and beyond. Journal of Psychotherapy Integration. 2020;30(2):160-173. doi:10.1037/int0000218	(55)	1		1		1	1			1								1		
Damschroder LJ, Aron DC, Keith RE, Kirsh SR, Alexander JA, Lowery JC. Fostering implementation of health services research findings into practice: A consolidated framework for advancing implementation science. Implementation Science. 2009;4. doi:10.1186/1748-5908-4-50	(56)	1		1	1	1						1						1		
Damschroder LJ, Reardon CM, Widerquist MAO, Lowery J. The updated Consolidated Framework for Implementation Research based on user feedback. Implementation Science. 2022;17(1). doi:10.1186/s13012-022-01245-0	(57)	1		1	1	1						1						1		

Reference		Quality criteria of technology	Regularisaton	Quality criteria of professionals	Quality criteria of users	Quality criteria of organisations	Examples of governance	Examples of reports	Social work	Mental health care	Strict criteria	Growth path to quality	Meta thinking on quality	Flanders	Belgium	Netherlands	Europe	World, applicable	World, none applicable	Interpretation/ summary
de Hond AAH, Leeuwenberg AM, Hooft L, et al. Guidelines and quality criteria for artificial intelligence-based prediction models in healthcare: a scoping review. Npj Digital Medicine. 2022;5(1). doi:10.1038/s41746-021-00549-7	(58)	1		1	1	1				1								1		1
De Jaegere E. Ethische richtlijnen voor 'technology-based' suïcidepreventie programma's. https://www.flanderscare.be/sites/default/files/media/ethiek_FCCOH.pdf	(59)	1								1				1						
De Marez L, Georges A, Sevenhant R, Devos E. Imec.digimeter.2024. In: Digitale trends in Vlaanderen. Imec. 2025. doi:https://www.imec.be/nl/kennisuitwisseling/techmeters/digimeter/imecdigimeter-2024	(60)				1									1						
Departement Werk, Economie, Wetenschap, Innovatie en Sociale Economie. Bletchley-verklaring: eerste internationale verklaring over veilige ontwikkeling van artificiële intelligentie (AI). Vlaanderen. 2023. https://www.ewi-vlaanderen.be/nieuws/bletchley-verklaring-eerste-internationale-verklaring-over-veilige-ontwikkeling-van?utm_medium=email&utm_campaign=Nieuwsbrief%20EWI%20%2014%20november%202023&utm_content=Nieuwsbrief%20EWI%20%2014%20november%202023+CID_95d794573cb316353d3f5852e1df6451&utm_source=Email%20marketing%20software&utm_term=Bletchley-verklaring%20eerste%20internationale%20verklaring%20over%20veilige%20ontwikkeling%20van%20artificile%20intelligentie%20AI	(61)	1					1					1						1		1
Departement Werk, Economie, Wetenschap, Innovatie en Sociale Economie. The Flemish AI Plan. https://www.ewi-vlaanderen.be/en/flemish-ai-plan	(62)						1							1						

Reference		Quality criteria of technology	Regularisaton	Quality criteria of professionals	Quality criteria of users	Quality criteria of organisations	Examples of governance	Examples of reports	Social work	Mental health care	Strict criteria	Growth path to quality	Meta thinking on quality	Flanders	Belgium	Netherlands	Europe	World, applicable	World, none applicable	Interpretation/ summary
Departement Werk, Economie, Wetenschap, Innovatie en Sociale Economie. Transities mogelijk maken in tijden van disruptie. Vlaanderen. 2023. https://www.ewi-vlaanderen.be/nieuws/transities-mogelijk-maken-tijden-van-disruptie	(63)	1	1				1					1					1	1	1	1
Departement Zorg. Alivia: uw digitaal zorg- en ondersteuningsplan. Vlaanderen. https://www.zorg-en-gezondheid.be/alivia-uw-digitaal-zorg-en-ondersteuningsplan	(64)	1		1	1	1								1						
Departement Zorg. BelRAI in Vlaanderen. Vlaanderen. https://www.departementzorg.be/nl/belrai-vlaanderen	(65)					1								1						
Departement Zorg. Vlaams Agentschap voor de Samenwerking rond Gegevensdeling tussen de Actoren in de Zorg (VASGAZ): wie of wat is het agentschap VASGAZ? 2017. https://www.departementwvg.be/wie-wat-het-agentschap	(66)						1			1				1						
Department of Health, Disability and Ageing. Understanding regulation of software-based medical devices. Australian Government. 2022. https://www.tga.gov.au/how-we-regulate/manufacturing/medical-devices/manufacturer-guidance-specific-types-medical-devices/regulation-software-based-medical-devices	(67)	1	1							1									1	
Developers Android. Core app quality. Android. https://developer.android.com/docs/quality-guidelines/core-app-quality#listing	(68)	1										1						1		
Devlieghere J, Roose R, Evans T. Managing the electronic turn. European Journal of Social Work. 2019;23(5):767-778. doi:10.1080/13691457.2019.1582009	(69)	1		1	1	1			1					1						

Reference		Quality criteria of technology	Regularisaton	Quality criteria of professionals	Quality criteria of users	Quality criteria of organisations	Examples of governance	Examples of reports	Social work	Mental health care	Strict criteria	Growth path to quality	Meta thinking on quality	Flanders	Belgium	Netherlands	Europe	World, applicable	World, none applicable	Interpretation/ summary
Digitaal Vlaanderen. Digitale dienstverleningsstrategie voor Vlaamse overheden. Vlaanderen. 2022. https://www.vlaanderen.be/digitaal-vlaanderen/vlaamse-digitale-strategie/digitale-dienstverleningsstrategie	(70)	1				1	1							1						
Digitaal Vlaanderen. Meetinstrument digitale vaardigheden. Vlaanderen https://www.vlaanderen.be/samenleven/toegankelijkheid-e-inclusie/e-inclusie/meetinstrument-digitale-vaardigheden	(71)				1									1						
Digitaal Vlaanderen. Vademecum bij gebruik aftoetsingsinstrument: Handleiding voor het aftoetsingsinstrument voor digitale dienstverlening. Vlaamse Overheid. 2023. https://www.vlaanderen.be/digitaal-vlaanderen/vlaamse-digitale-strategie/digitale-dienstverleningsstrategie	(72)	1												1						
Digitaal Vlaanderen. Vlaamse strategie informatieveiligheid. https://www.vlaanderen.be/digitaal-vlaanderen/vlaamse-digitale-strategie/vlaamse-strategie-informatieveiligheid	(73)	1				1	1							1						
Digitale Zorg Gids. Hulp bij digitale zorg. https://www.digitalezorggids.nl/hulp-bij-digitale-zorg/	(74)				1											1				
Digitall. Wat is de Digital Inclusion bij Design Index? https://digitalinclusionindex.digitall.be/nl#:~:text=De%20Digital%20Inclusion%20by%20Design%20Index%20toont%20u%20hoe%20toegankelijk,zoals%20marketeers%2C%20productontwerpers%20en%20webdesigners	(75)	1									1				1					

Reference		Quality criteria of technology	Regularisaton	Quality criteria of professionals	Quality criteria of users	Quality criteria of organisations	Examples of governance	Examples of reports	Social work	Mental health care	Strict criteria	Growth path to quality	Meta thinking on quality	Flanders	Belgium	Netherlands	Europe	World, applicable	World, none applicable	Interpretation/ summary
Digivaardig in de zorg. Zelfscan digitale vaardigheden welzijn. https://www.digivaardigindezorg.nl/welzijn-sociaal-werk/home/zelftest/#:~:text=Met%20de%20zelfscan%20digitale%20vaardigheden,geef%20zo%20zorgvuldig%20mogelijk%20antwoord.	(76)			1		1		1	1		1					1				
Digivaardig in de zorg. Zo meet je de stand van digitale vaardigheden in jouw organisatie. 2019. https://www.digivaardigindezorg.nl/zo-meet-je-de-stand-van-de-digitale-vaardigheden-in-jouw-organisatie/	(77)			1		1										1				
Drude KP, Maheu MM. The Need for Interprofessional Consensus about Telebehavioral Health Competencies and Education. J Technol Behav Sci. 2024;9(1):7-13. doi:10.1007/s41347-023-00369-4	(78)			1			1											1		
EFQM. Model EFQM. 2019. https://efqm.org/the-efqm-model/	(79)					1	1											1		
EHealth Tools Assessment. Assessment Instrument. https://ehealth-criteria-toolbox.net/assessment-instrument/	(80)	1								1	1							1		
eHealth. eHealth Welcome Pack. Belgium. 2023. https://www.ehealth.fgov.be/ehealthplatform/nl	(81)	1	1			1				1					1					
eHealth-platform. CoBRHA – Common Base Registry for HealthCare Actor. 2022. https://www.ehealth.fgov.be/ehealthplatform/nl/service-cobrha-common-base-registry-for-healthcare-actor	(82)	1				1	1			1					1					
EIT Health. European Taskforce for Harmonised Evaluations of Digital Medical Devices (DMDs). https://eithealth.eu/external-collaborations/european-taskforce-for-harmonised-evaluations-of-digital-medical-devices-dmds/	(83)						1			1							1			

Reference		Quality criteria of technology	Regularisaton	Quality criteria of professionals	Quality criteria of users	Quality criteria of organisations	Examples of governance	Examples of reports	Social work	Mental health care	Strict criteria	Growth path to quality	Meta thinking on quality	Flanders	Belgium	Netherlands	Europe	World, applicable	World, none applicable	Interpretation/ summary
EIT Health. Towards a European evaluation framework for digital medical devices (DMDs) in the European Union – Launch of a European taskforce. 2022. https://eithealth.eu/news-article/press-release-digital-medical-devices-launch-of-a-european-taskforce/	(84)	1					1					1	1				1			
ETSI. Cyber security for consumer internet of things (ETSI EN 303 645 V2.1.1). 2020. https://www.etsi.org/deliver/etsi_en/303600_303699/303645/02.01.01_60/en_303645v020101p.pdf	(85)	1	1															1		
EU Cloud COC. https://eucoc.cloud/en/home	(86)	1	1														1			
European Commission, Directorate-General for Internal Market, Industry, Entrepreneurship and SMEs. Manufacturers – CE marking for goods in the EU internal market. https://single-market-economy.ec.europa.eu/single-market/goods/ce-marking/manufacturers_en	(87)	1	1							1							1			
European Commission. Commission Welcomes Political Agreement on Cyber Resilience Act. European Union; 2023. https://ec.europa.eu/commission/presscorner/detail/en/ip_23_6168	(88)	1	1			1											1			
European Commission. EU4Health programme 2021-2027 – a vision for a healthier European Union. 2021. https://health.ec.europa.eu/funding/eu4health-programme-2021-2027-vision-healthier-european-union_en#work-programmes	(89)	1	1	1			1			1							1			
European Commission. The EU Cybersecurity Act. European Union. 2025. https://digital-strategy.ec.europa.eu/en/policies/cybersecurity-act	(90)	1	1								1						1			
European Commission. The EU cybersecurity certification framework. European Union; 2025. https://digital-strategy.ec.europa.eu/en/policies/cybersecurity-certification-framework	(91)	1				1											1			

Reference		Quality criteria of technology	Regularisaton	Quality criteria of professionals	Quality criteria of users	Quality criteria of organisations	Examples of governance	Examples of reports	Social work	Mental health care	Strict criteria	Growth path to quality	Meta thinking on quality	Flanders	Belgium	Netherlands	Europe	World, applicable	World, none applicable	Interpretation/ summary
European Data Protection Board. Guidelines on deceptive design patterns in social media platform interfaces. European Union. 2022. https://www.edpb.europa.eu/our-work-tools/our-documents/guidelines/guidelines-032022-deceptive-design-patterns-social-media_en	(92)	1			1							1					1			
European Data Protection Board. Richtsnoeren inzake toestemming overeenkomstig. European Union. 2022. https://edpb.europa.eu/our-work-tools/our-documents/guidelines/guidelines-052020-consent-under-regulation-2016679_nl	(93)	1		1		1					1						1			
European Parliament. Shaping the digital transformation: EU strategy explained. 2021. https://www.europarl.europa.eu/topics/en/article/20210414STO02010/shaping-the-digital-transformation-eu-strategy-explained	(94)						1										1			
European Union. Directive (EU) 2016/1148 of the European Parliament and of the Council of 6 July 2016 concerning measures for a high common level of security of network and information systems across the Union. Off J Eur Union. 2016;L194:1-30. http://data.europa.eu/eli/dir/2016/1148/oj	(95)	1	1			1	1				1						1			
European Union. Directive (EU) 2019/882 of the European Parliament and of the Council of 17 April 2019 on the accessibility requirements for products and services (European Accessibility Act). Official Journal of the European Union. 2019;L151:70–115. Available at: http://data.europa.eu/eli/dir/2019/882/oj. Accessed November 25, 2025.	(96)	1	1		1		1				1						1			

Reference		Quality criteria of technology	Regularisaton	Quality criteria of professionals	Quality criteria of users	Quality criteria of organisations	Examples of governance	Examples of reports	Social work	Mental health care	Strict criteria	Growth path to quality	Meta thinking on quality	Flanders	Belgium	Netherlands	Europe	World, applicable	World, none applicable	Interpretation/ summary
European Union. Directive (EU) 2022/2555 of the European Parliament and of the Council of 14 December 2022 on measures for a high common level of cybersecurity across the Union, amending Regulation (EU) No 910/2014 and Directive (EU) 2018/1972, and repealing Directive (EU) 2016/1148 (NIS 2 Directive) [Directive]. Official Journal of the European Union. 2022; L 333, 80–152. http://data.europa.eu/eli/dir/2022/2555/oj	(97)	1	1			1											1			
European Union. Regulation (EU) 2016/679 of the European Parliament and of the Council of 27 April 2016 on the protection of natural persons with regard to the processing of personal data and on the free movement of such data, and repealing Directive 95/46/EC (General Data Protection Regulation) [Regulation]. Official Journal of the European Union. 2016; L 119, 1–88. https://eur-lex.europa.eu/eli/reg/2016/679/oj	(98)	1	1	1													1			
European Union. Regulation (EU) 2017/745 of the European Parliament and of the Council of 5 April 2017 on medical devices, amending Directive 2001/83/EC, Regulation (EC) No 178/2002 and Regulation (EC) No 1223/2009, and repealing Council Directives 90/385/EEC and 93/42/EEC [Regulation]. Official Journal of the European Union. 2017; L 117, 1–175. https://eur-lex.europa.eu/legal-content/EN/TXT/?uri=CELEX%3A32017R0745	(99)	1	1							1							1			
European Union. Regulation (EU) 2024/1689 of the European Parliament and of the Council of 13 June 2024 laying down harmonised rules on artificial intelligence. Official Journal of the European Union.2024; 1689, 1–144. http://data.europa.eu/eli/reg/2024/1689/oj	(100)	1	1														1			1

Reference		Quality criteria of technology	Regularisaton	Quality criteria of professionals	Quality criteria of users	Quality criteria of organisations	Examples of governance	Examples of reports	Social work	Mental health care	Strict criteria	Growth path to quality	Meta thinking on quality	Flanders	Belgium	Netherlands	Europe	World, applicable	World, none applicable	Interpretation/ summary
European Union. Regulation (EU) 2024/2847 of the European Parliament and of the Council of 23 October 2024 on horizontal cybersecurity requirements for products with digital elements and amending Regulations (EU) No 168/2013 and (EU) No 2019/1020 and Directive (EU) 2020/1828 (Cyber Resilience Act) [Regulation]. Official Journal of the European Union. 2024; L 2847, 1–66. http://data.europa.eu/eli/reg/2024/2847/oj	(101)	1	1			1									1					
European Union. Regulation (EU) 2025/327 of the European Parliament and of the Council of 11 February 2025 on the European Health Data Space and amending Directive 2011/24/EU and Regulation (EU) 2024/2847. Off J Eur Union. 2025;L5:1-96. https://eur-lex.europa.eu/eli/reg/2025/327/oj	(102)		1		1	1	1			1							1			
Extra Horizon. All you need to know about IEC 62304 compliant software development. 2023. https://www.extrahorizon.com/iec-62304-software-development-medical-devices	(103)	1	1							1								1		1
Eysenbach G, CONSORT-EHEALTH Group. Improving and Standardizing Evaluation Reports of Web-based and Mobile Health Interventions. Journal of Medical Internet Research. 2011;13(4). doi:10.2196/jmir.1923	(104)	1								1			1					1		
Faure L, Brotcorne P, Vendramin P, Mariën I. Barometer Digitale Inclusie 2022. Koning Boudewijnstichting. 2022. doi:https://kbs-frb.be/nl/barometer-digitale-inclusie-2022	(105)				1										1					
Federaal Agentschap voor Geneesmiddelen en Gezondheidsproducten (FAGG). Medische hulpmiddelen en hun hulpstukken. FAGG. 2025. https://www.fagg.be/nl/MENSELIJK_gebruik/gezondheidsproducten/medische_hulpmiddelen_hulpstukken	(106)	1	1				1			1	1				1					

Reference		Quality criteria of technology	Regularisaton	Quality criteria of professionals	Quality criteria of users	Quality criteria of organisations	Examples of governance	Examples of reports	Social work	Mental health care	Strict criteria	Growth path to quality	Meta thinking on quality	Flanders	Belgium	Netherlands	Europe	World, applicable	World, none applicable	Interpretation/ summary
Federaal Kenniscentrum voor de Gezondheidszorg KCE. Moeten medische apps worden vergoed? En zo ja, welke? 2023. https://kce.fgov.be/en/kce-press-release-kce-reports-362-evaluation-of-digital-medical-technologies	(107)	1								1					1					
Federal Institute for Drugs and Medical Devices. Digital Health Applications (DiGA). 2020. https://www.bfarm.de/EN/Medical-devices/Tasks/DiGA-and-DiPA/Digital-Health-Applications/_node.html	(108)	1			1		1			1							1			
Federale Overheid. Wet van 7 april 2019 tot vaststelling van een kader voor de beveiliging van netwerk- en informatiesystemen van algemeen belang voor de openbare veiligheid. Belgisch Staatsblad. Published online 2019. https://economie.fgov.be/nl/legislation/wet-van-7-april-2019-tot	(109)	1	1				1				1				1					
Federale Overheidsdienst Volksgezondheid. Actieplan e-Gezondheid 2022-2024. 2022. https://www.health.belgium.be/sites/default/files/uploads/fields/fpshealth_theme_file/actieplan_egezondheid_2022-2024_protocolakkoord.pdf	(110)	1		1	1	1	1			1					1					
Fransen J. Methodiek online hulpverlening voor schoolmaatschappelijk werk. Hogeschool Inholland; 2011.	(111)	1	1							1								1		
Frederix P, Drooghmans N, Vandries T. Handelingskader "Omgaan met online risico's vanuit het denkkader emotionele ontwikkeling.". https://research-expertise.ucll.be/sites/default/files/2021-12/Handelingskader%20Final%20Layout%20V3%281%29.pdf	(112)	1		1	1	1			1					1						
Friedman V. How to make a strong case for accessibility. Smart Interface Design Patterns. 2023. https://smart-interface-design-patterns.com/articles/accessibility-strong-case/	(113)	1										1						1		1

Reference		Quality criteria of technology	Regularisaton	Quality criteria of professionals	Quality criteria of users	Quality criteria of organisations	Examples of governance	Examples of reports	Social work	Mental health care	Strict criteria	Growth path to quality	Meta thinking on quality	Flanders	Belgium	Netherlands	Europe	World, applicable	World, none applicable	Interpretation/ summary
Friedman V. Mobile accessibility target sizes. Smart Interface Design Patterns. 2023. https://smart-interface-design-patterns.com/articles/accessible-tap-target-sizes/	(114)	1									1							1		1
Gaglio B, Shoup JA, Glasgow RE. The RE-AIM framework: a systematic review of use over time. Am J Public Health. 2013;103(6):e38–e46. PMID:23597377 https://doi.org/10.2105/ajph.2013.301299	(115)					1	1											1		1
Gegevensbeschermingsautoriteit. De GBA publiceert een checklist voor het correcte gebruik van cookies. 2023. https://www.gegevensbeschermingsautoriteit.be/burger/nieuws/2023/10/20/de-gba-publiceert-een-checklist-voor-het-correcte-gebruik-van-cookies	(116)	1					1				1				1					1
GGD AppStore. De testmethode: GGD AppStore methodiek. 2016. https://www.ggdappstore.nl/Appstore/Testmethode	(117)	1					1	1		1		1				1				
GGZ Standaarden. Digitale zorg. 2021. https://www.ggzstandaarden.nl/zorgstandaarden/ehealth/introductie	(118)	1		1	1	1	1			1						1				
Goubin E. Communicatie als bindkracht: Tien communicatie-uitdagingen voor een sterke democratie en een warme samenleving. Brussel: Politeia; 2023. https://www.politeia.be/nl/publicaties/357512-communicatie+als+bindkracht	(119)	1		1	1	1	1		1			1		1						
Government UK. The Bletchley Declaration by countries attending the AI Safety Summit. 2023. https://www.gov.uk/government/publications/ai-safety-summit-2023-the-bletchley-declaration/the-bletchley-declaration-by-countries-attending-the-ai-safety-summit-1-2-november-2023	(120)	1					1					1						1		
Groves K. CAPTCHA-less security. 2012. https://karlgroves.com/captcha-less-security/	(121)	1										1	1				1			

Reference		Quality criteria of technology	Regularisaton	Quality criteria of professionals	Quality criteria of users	Quality criteria of organisations	Examples of governance	Examples of reports	Social work	Mental health care	Strict criteria	Growth path to quality	Meta thinking on quality	Flanders	Belgium	Netherlands	Europe	World, applicable	World, none applicable	Interpretation/ summary
Guyatt GH, Oxman AD, Kunz R, et al. Going from evidence to recommendations. BMJ. 2008;336(7652):1049-1051. doi:10.1136/bmj.39493.646875.ae	(122)	1											1					1		
Guyatt GH, Oxman AD, Vist GE, et al. GRADE: an emerging consensus on rating quality of evidence and strength of recommendations. BMJ. 2008;336(7650):924-926. doi:10.1136/bmj.39489.470347.ad	(123)	1											1					1		
Han Y, Itälä T, Hämäläinen M. Citizen Centric Architecture approach - taking e-health forward by integrating citizens and service providers. Studies in health technology and informatics. 2010;160(2):907-911. doi:10.3233/978-1-60750-588-4-907	(124)	1								1			1					1		
Harris S. Australian social workers' understandings of technology in practice. Australian Social Work. 2022;75(4):420-432. doi:10.1080/0312407X.2021.1949025	(125)	1		1	1	1			1										1	
Hartman-van der Laan, M. Sociaal werk in de digitale samenleving. Coutinho. 2019. https://www.coutinho.nl/nl/sociaal-werk-in-de-digitale-samenleving-9789046906590	(126)			1		1			1							1				
Harvard Business School Online. 5 steps in the change management process. HBS Online Business Insights Blog. 2020. https://online.hbs.edu/blog/post/change-management-process	(127)			1		1	1					1	1					1		1

Reference		Quality criteria of technology	Regularisaton	Quality criteria of professionals	Quality criteria of users	Quality criteria of organisations	Examples of governance	Examples of reports	Social work	Mental health care	Strict criteria	Growth path to quality	Meta thinking on quality	Flanders	Belgium	Netherlands	Europe	World, applicable	World, none applicable	Interpretation/ summary
Health Canada. Guidance Document: Software as a Medical Device (SaMD) – Definition and Classification (Catalogue No. H164-291/2-2019E-PDF). Ottawa, ON: Health Canada. 2019. https://www.canada.ca/en/health-canada/services/drugs-health-products/medical-devices/application-information/guidance-documents/software-medical-device-guidance.html	(128)	1	1							1									1	
Helfrich CD, Damschroder LJ, Hagedorn HJ, et al. A critical synthesis of literature on the promoting action on research implementation in health services (PARIHS) framework. Implementation Science. 2010;5(1). doi:10.1186/1748-5908-5-82	(129)	1				1												1		1
Henson P, David G, Albright K, Torous J. Deriving a practical framework for the evaluation of health apps. The Lancet Digital Health. 2019;1(2):52-54. doi:10.1016/s2589-7500(19)30013-5	(130)	1								1								1		
HL7 FHIR Foundation. Welcome to the HL7 FHIR Foundation. 2024. https://www.fhir.org/	(131)	1		1		1	1			1	1		1				1			
HL7 International. FHIR Release 5 (version 5.0.0). 2024. https://hl7.org/fhir/	(132)	1		1		1	1											1		
Hoogendoorn P, Versluis A, Kampen S, et al. What Makes a Quality Health App—Developing a Global Research-Based Health App Quality Assessment Framework for CEN-ISO/TS 82304-2: Delphi Study. JMIR Formative Research. 2023;7. doi:10.2196/43905	(133)	1	1															1		
Hui K, Gorin M, Sisti D. A Call for Greater Regulation of Digital Mental Health Technologies. AJOB Neuroscience. 2022;13(3):193-195. doi:10.1080/21507740.2022.2082595	(134)	1	1							1								1		1

Reference		Quality criteria of technology	Regularisaton	Quality criteria of professionals	Quality criteria of users	Quality criteria of organisations	Examples of governance	Examples of reports	Social work	Mental health care	Strict criteria	Growth path to quality	Meta thinking on quality	Flanders	Belgium	Netherlands	Europe	World, applicable	World, none applicable	Interpretation/ summary
Inspectie Gezondheidszorg en Jeugd. Gehandicaptenzorg worstelt met digitale vormen van zorg. 2023. https://www.igj.nl/actueel/nieuws/2023/06/16/gehandicaptenzorg-worstelt-met-digitale-vormen-van-zorg	(135)						1									1				
Inspectie Gezondheidszorg en Jeugd. Toetsingskader Digitale Zorg – uitgebreide versie. Ministerie van Volksgezondheid, Welzijn en Sport. 2024. https://www.igj.nl/publicaties/toetsingskaders/2024/05/06/toetsingskader-digitale-zorg-uitgebreide-versie	(136)	1	1			1	1				1	1				1				
International Electrotechnical Commission. IEC 62366-1:2015 – Medical devices – Part 1: Application of usability engineering to medical devices. IEC. 2015. https://www.iso.org/standard/63179.html	(137)	1								1								1		
International Electrotechnical Commission. IEC 62304:2006/Amd 1:2015 – Medical device software — Software life cycle processes — Amendment 1. International Electrotechnical Commission. 2015. https://www.iso.org/standard/64686.html	(138)	1	1							1								1		
International Medical Device Regulators Forum. Software as a medical device (SaMD). 2017. https://www.imdrf.org/working-groups/software-medical-device-samd	(139)	1								1								1		
International Medical Device Regulators Forum. Software as a Medical Device Possible Framework for Risk Categorization and Corresponding Considerations. 2014. https://www.imdrf.org/sites/default/files/docs/imdrf/final/technical/imdrf-tech-140918-samd-framework-risk-categorization-141013.pdf	(140)	1								1									1	

Reference		Quality criteria of technology	Regularisaton	Quality criteria of professionals	Quality criteria of users	Quality criteria of organisations	Examples of governance	Examples of reports	Social work	Mental health care	Strict criteria	Growth path to quality	Meta thinking on quality	Flanders	Belgium	Netherlands	Europe	World, applicable	World, none applicable	Interpretation/ summary
International Organization for Standardization & International Electrotechnical Commission. ISO/IEC 27001:2022 – Information security, cybersecurity and privacy protection – Information security management systems – Requirements. ISO/IEC. 2022. https://www.iso.org/standard/27001.html	(141)	1	1															1		
International Organization for Standardization & International Electrotechnical Commission. ISO/IEC 27701:2019 – Privacy information management – Extension to ISO/IEC 27001 and ISO/IEC 27002 for privacy information management systems (1st ed.). ISO/IEC. 2019. https://www.iso.org/standard/71670.html	(142)	1	1			1												1		
International Organization for Standardization. ISO 13485:2016 – Medical devices – Quality management systems – Requirements for regulatory purposes (2nd ed.). ISO. 2016 https://www.iso.org/standard/59752.html	(143)	1	1							1								1		
International Organization for Standardization. ISO 24495 1:2023 – Plain language — Part 1: Governing principles and guidelines (1st ed.). ISO. 2023. https://www.iso.org/standard/78907.html	(144)	1		1		1												1		1
International Organization for Standardization. ISO 27269:2021. Health informatics – International patient summary. 2021. https://www.iso.org/standard/79491.html	(145)	1				1	1										1			
International Organization for Standardization. ISO 9001:2015 – Quality management systems – Requirements (5th ed.). ISO. 2015. https://www.iso.org/standard/62085.html	(146)	1				1												1		
International Organization for Standardization. ISO 9241-210:2019 – Ergonomics of human-system interaction – Part 210: Human-centred design for interactive systems. ISO. 2019. https://www.iso.org/standard/77520.html	(147)	1																1		

Reference		Quality criteria of technology	Regularisaton	Quality criteria of professionals	Quality criteria of users	Quality criteria of organisations	Examples of governance	Examples of reports	Social work	Mental health care	Strict criteria	Growth path to quality	Meta thinking on quality	Flanders	Belgium	Netherlands	Europe	World, applicable	World, none applicable	Interpretation/ summary
International Organization for Standardization. ISO/IEEE 11073 10418:2014 – Health informatics – Personal health device communication – Part 10418: Device specialization – International Normalized Ratio (INR) monitor. ISO and IEEE. 2014. https://www.iso.org/standard/61897.html	(148)	1									1						1			
International Organization for Standardization. ISO/TS 82304-2:2021 – Health software – Part 2: Health and wellness apps – Quality and reliability (1st ed.). ISO. 2021. https://www.iso.org/standard/78182.html	(149)	1								1							1			
Itchhaporia D. The evolution of the Quintuple Aim: health equity, health outcomes, and the economy. J Am Coll Cardiol. 2021;78(22):2262-2264. doi:10.1016/j.jacc.2021.10.018	(150)	1		1		1	1			1								1		1
Jacob C, Lindeque J, Klein A, Ivory C, Heuss S, Peter MK. Assessing the Quality and Impact of eHealth Tools. Systematic Literature Review and Narrative Synthesis JMIR Human Factors. 2023;10. doi:10.2196/45143	(151)	1		1		1				1								1		1
Jacob C, Lindeque J, Müller R, et al. A sociotechnical framework to assess patient-facing eHealth tools: results of a modified Delphi process. Npj Digital Medicine. 2023;6(1). doi:10.1038/s41746-023-00982-w	(152)	1								1	1							1		
James HM, Papoutsi C, Wherton J, Greenhalgh T, Shaw SE. Spread, Scale-up, and Sustainability of Video Consulting in Health Care: Systematic Review and Synthesis Guided by the NASSS Framework. Journal of Medical Internet Research. 2021;23(1). doi:10.2196/23775	(153)	1		1	1	1												1		1

Reference		Quality criteria of technology	Regularisaton	Quality criteria of professionals	Quality criteria of users	Quality criteria of organisations	Examples of governance	Examples of reports	Social work	Mental health care	Strict criteria	Growth path to quality	Meta thinking on quality	Flanders	Belgium	Netherlands	Europe	World, applicable	World, none applicable	Interpretation/ summary
Janssen R, Bodenstaff L, Gyaltsen-Lohuis E. Succesvol ondernemen met e-health: Innovatieroutes in de zorg. Hogeschool Windesheim. 2013. https://www.windesheim.nl/onderzoekspublicaties/succesvol-ondernemen-met-e-health	(154)	1		1	1	1	1									1				
Jones RB, Stallard P, Agha SS, et al. Practitioner review: Co design of digital mental health technologies with children and young people. Journal of Child Psychology and Psychiatry. 2020;61(8):928-940. doi:10.1111/jcpp.13258	(155)	1								1								1		1
Kenniscentrum Data & Maatschappij. Zorgt AI voor kansen of uitdagingen op vlak van digitale inclusie? 2024. https://data-en-maatschappij.ai/thema/digitale-inclusie	(156)	1										1		1						
Kenniscentrum Digisprong. DigCompEdu: Europees referentiekader voor digitale competenties van leraren. Vlaanderen. https://www.vlaanderen.be/kenniscentrum-digisprong/themas/professionalisering/digcompedu-europees-referentiekader-voor-digitale-competenties-van-leraren	(157)			1																
Kenniscentrum Digitale Zorg. Leidraad applicaties en algoritmes in de zorg. Zorgverzekeraars Nederland. https://www.zn.nl/app/uploads/2023/03/KC.DZ_Leidraad_applicaties_en_algoritmes_versie1.6-.xlsx	(158)	1									1					1				
Kennisplein Gehandicaptensector. Waarom NEN 7510 relevant is voor jou! 2024. https://www.kennispleingehandicaptensector.nl/tips-tools/tips/waarom-nen-7510-relevant-is-voor-jou	(159)	1								1						1				1
Kidholm K, Ekeland AG, Jensen LK, et al. A MODEL FOR ASSESSMENT OF TELEMEDICINE APPLICATIONS: MAST. International Journal of Technology Assessment in Health Care. 2012;28(1):44-51. doi:10.1017/s0266462311000638	(160)	1								1							1			

Reference		Quality criteria of technology	Regularisaton	Quality criteria of professionals	Quality criteria of users	Quality criteria of organisations	Examples of governance	Examples of reports	Social work	Mental health care	Strict criteria	Growth path to quality	Meta thinking on quality	Flanders	Belgium	Netherlands	Europe	World, applicable	World, none applicable	Interpretation/ summary
Kip H, Wentzel J, Kelders SM. Shaping Blended Care: Adapting an Instrument to Support Therapists in Using eMental Health. JMIR Mental Health. 2020;7(11). doi:10.2196/24245	(161)				1					1								1		
Kretzschmar K, Tyroll H, Pavarini G, Manzini A, Singh I, Group NYPA. Can Your Phone Be Your Therapist? Young People's Ethical Perspectives on the Use of Fully Automated Conversational Agents (Chatbots) in Mental Health Support. Biomedical Informatics Insights. 2019;11. doi:10.1177/1178222619829083	(162)	1	1							1								1		
Kristensen FB, Lampe K, Wild C, Cerbo M, Goettsch W, Becla L. The HTA Core Model ® —10 Years of Developing an International Framework to Share Multidimensional Value Assessment. Value in Health. 2017;20(2):244-250. doi:10.1016/j.jval.2016.12.010	(163)	1	1							1	1						1			
Kruispuntbank van de Sociale Zekerheid (KSZ). Minimale normen – informatieveiligheidsbeleid. https://www.ksz-bcss.fgov.be/nl/gegevensbescherming/informatieveiligheidsbeleid	(164)	1				1					1				1					
Label2Enable Consortium. Label2Enable: Promoting a quality label for health apps. 2024. https://label2enable.eu/	(165)	1	1				1			1	1		1			1	1			
Lattie EG, Stiles-Shields C, Graham AK. An overview of and recommendations for more accessible digital mental health services. Nature Reviews Psychology. 2022;1(2):87-100. doi:10.1038/s44159-021-00003-1	(166)	1								1								1		
Lewin S, Glenton C, Munthe Kaas H, et al. Using qualitative evidence in decision making for health and social interventions: An approach to assess confidence in findings from qualitative evidence syntheses (GRADE CERQual. PLOS Medicine. 2015;12(10). doi:10.1371/journal.pmed.1001895	(167)	1											1					1		

Reference		Quality criteria of technology	Regularisaton	Quality criteria of professionals	Quality criteria of users	Quality criteria of organisations	Examples of governance	Examples of reports	Social work	Mental health care	Strict criteria	Growth path to quality	Meta thinking on quality	Flanders	Belgium	Netherlands	Europe	World, applicable	World, none applicable	Interpretation/ summary
Liverpool S, Mota CP, Sales CMD, et al. Engaging children and young people in digital mental health interventions: Systematic review of modes of delivery, facilitators, and barriers. Journal of Medical Internet Research. 2020;22(6). doi:10.2196/16317	(168)	1								1								1		1
Lopes IM, Guarda T, Oliveira P. How ISO 27001 can help achieve GDPR compliance. In: Rocha A, Pedrosa I, Cota MP, Goncalves R, eds. Proceedings of the 14th Iberian Conference on Information Systems and Technologies (CISTI 2019) (Article 8760937. IEEE Computer Society Press; 2019. doi:10.23919/CISTI.2019.8760937	(169)	1	1															1		1
Lucas C, Verhasselt P. Van ambitie tot adoptie: Digitaal transformeren met impact. Die Keure. 2023. https://www.diekeure.be/professional/nl/product/van-ambitie-tot	(170)			1		1								1						
Maatman S. Op weg naar kwaliteitscriteria voor gemixte participatie: Een verkennend onderzoek naar de kwaliteitswaarborging van gemixte participatietrajecten [Masterthesis. 2022. https://theses.ubn.ru.nl/bitstreams/c33bf566-30d4-4e7f-9a55-aa5c9bafa11c/download	(171)	1			1											1				
Mattar J, Ramos DK, Lucas MR. DigComp-Based Digital competence Assessment Tools: Literature Review and Instrument Analysis. Education and Information Technologies. 2022;27(8):10843-10867. doi:10.1007/s10639-022-11034-3	(172)			1													1			
McDonald A, Eccles JA, Fallahkhair S, Critchley HD. Online psychotherapy: trailblazing digital healthcare. BJPsych Bulletin. 2019;44(2):60-66. doi:10.1192/bjb.2019.66	(173)	1		1														1		

Reference		Quality criteria of technology	Regularisaton	Quality criteria of professionals	Quality criteria of users	Quality criteria of organisations	Examples of governance	Examples of reports	Social work	Mental health care	Strict criteria	Growth path to quality	Meta thinking on quality	Flanders	Belgium	Netherlands	Europe	World, applicable	World, none applicable	Interpretation/ summary
McGrath P, Wozney L, Rathore SS, Notarianni M, Schellenberg M. Toolkit for E-Mental Health Implementation. Mental Health Commission of Canada; 2018. https://www.mentalhealthcommission.ca/professional-resources/ementalhealth/toolkit-for-e-mental-health-implementation/	(174)			1		1				1								1		
Mediawijs. Aan de slag als digihelper. 2022. https://assets.mediawijs.be/2022-06/mediawijs_digitaleinclusie_nl.pdf.	(175)			1					1			1		1						
Mediawijs. Beleidstool digitale inclusie – welzijn. 2020. https://www.mediawijs.be/nl/tools/beleidstool-digitale-inclusie-welzijn	(176)	1					1		1			1		1						
Mediawijs. Digitale inclusie. 2023. https://www.mediawijs.be/nl/artikels/wat-digitale-inclusie	(177)			1					1	1		1		1						
Mediawijs. Hoe digitaal inclusief is jouw product of dienst? 2020. https://www.mediawijs.be/nl/tools/hoe-digitaal-inclusief-jouw-product-dienst	(178)	1				1	1	1	1			1	1	1						
Mediawijs. Inclusion by design. https://www.mediawijs.be/nl/dossiers/inclusion-design	(179)	1				1					1			1						
Mediawijs. Wanneer ben je mediawijs? 2024. https://www.mediawijs.be/nl/mediawijsheid	(180)				1				1	1		1		1						
Medical Device Coordination Group. Guidance on qualification and classification of software in Regulation (EU) 2017/745 – MDR and Regulation (EU) 2017/746 – IVDR (MDCG 2019 11). European Commission. 2019. https://health.ec.europa.eu/system/files/2020-09/md_mdcg_2019_11_guidance_qualification_classification_software_en_0.pdf	(181)	1	1							1							1			

Reference		Quality criteria of technology	Regularisaton	Quality criteria of professionals	Quality criteria of users	Quality criteria of organisations	Examples of governance	Examples of reports	Social work	Mental health care	Strict criteria	Growth path to quality	Meta thinking on quality	Flanders	Belgium	Netherlands	Europe	World, applicable	World, none applicable	Interpretation/ summary
MedTech Europe & COCIR. Interoperability standards in digital health: A white paper from the medical technology industry. 2021. https://www.medtecheurope.org/wp-content/uploads/2021/10/mte_interoperability_digital_health_white-paper_06oct21.pdf	(182)	1									1						1			
Mental Health Commission of Canada. Toolkit for e Mental Health Implementation. 2018. https://mentalhealthcommission.ca/resource/toolkit-for-e-mental-health-implementation/	(183)	1								1									1	1
Mesiäislehto M, Kivipelto M, Hiilamo H. Social workers	(184)	1		1	1	1			1								1			
Mikolajczak J, Keijsers J, Henkemans OB. eHealth Analyse en SturingsInstrument (eASI. Tijdschrift Voor Gezondheidswetenschappen. 2011;89(2):78-82. doi:10.1007/s12508-011-0029-x	(185)	1								1						1				
Ministerie van Binnenlandse Zaken en Koninkrijksrelaties. Implementatiekader 'Verantwoorde inzet van algoritmen'. 2023. https://www.rijksoverheid.nl/documenten/rapporten/2023/06/30/implementatiekader-verantwoorde-inzet-van-algoritmen	(186)	1	1			1	1									1				
Ministerie van Volksgezondheid, Welzijn en Sport. Beslisboom betrouwbaarheidsniveaus en erkende inlogmiddelen. 2022. https://www.datavoorgezondheid.nl/documenten/2022/08/17/beslisboom-betrouwbaarheidsniveaus	(187)	1				1					1					1				
Mishna F, Milne B, Sanders J, Greenblatt A. Social Work Practice During COVID-19: Client Needs and Boundary Challenges. Global Social Welfare. 2021;9(2):113-120. doi:10.1007/s40609-021-00219-2	(188)			1	1	1			1			1	1					1		
Mozilla Foundation. Are mental health apps better or worse at privacy in 2023? 2023. https://foundation.mozilla.org/en/privacynotincluded/articles/are-mental-health-apps-better-or-worse-at-privacy-in-2023/	(189)	1	1															1		

Reference		Quality criteria of technology	Regularisaton	Quality criteria of professionals	Quality criteria of users	Quality criteria of organisations	Examples of governance	Examples of reports	Social work	Mental health care	Strict criteria	Growth path to quality	Meta thinking on quality	Flanders	Belgium	Netherlands	Europe	World, applicable	World, none applicable	Interpretation/ summary
Mstiaen P, Devriese S, Pouppez C, Roberfroid D, Savoye I. Videoconsultaties in de opvolging van patiënten met een chronische somatische aandoening. Health Services Research (HSR. Brussel Federaal Kenniscentrum voor de Gezondheidszorg (KCE. Published online 2020. doi:10.57598/R328AS	(190)	1		1	1		1				1			1						
Mulder, P. ISO normen: de betekenis en overzicht. Toolshero. 2025. https://www.toolshero.nl/kwaliteitsmanagement/iso-normen/	(191)	1																1		1
N.H.S. Digital Technology Assessment Criteria (DTAC). UK. 2021. https://transform.england.nhs.uk/key-tools-and-info/digital-technology-assessment-criteria-dtac/	(192)	1					1				1								1	
N.Q.A. ISO 13485:2016 – Medical devices management system implementation guide. 2016. https://www.nqa.com/medialibraries/NQA/NQA-Media-Library/PDFs/NQA-ISO-13485-Implementation-Guide.pdf	(193)	1	1							1								1		1
National Association of Social Workers (nasw).Standard for Technology in Social Work Practice. https://www.socialworkers.org/Practice/NASW-Practice-Standards-Guidelines/Standards-for-Technology-in-Social-Work-Practice	(194)	1							1									1		
National Institute for Health and Care Excellence. Evidence standards framework (ESF) for digital health technologies. https://www.nice.org.uk/corporate/ecd7	(195)	1								1	1							1		
National Institute of Standards and Technology (US). The NIST Cybersecurity framework (CSF) 2.0. 2024. https://doi.org/10.6028/NIST.CSWP.29.ipd	(196)	1				1													1	

Reference		Quality criteria of technology	Regularisaton	Quality criteria of professionals	Quality criteria of users	Quality criteria of organisations	Examples of governance	Examples of reports	Social work	Mental health care	Strict criteria	Growth path to quality	Meta thinking on quality	Flanders	Belgium	Netherlands	Europe	World, applicable	World, none applicable	Interpretation/ summary
Neal D, Engelsma T, Tan J, et al. Limitations of the new ISO standard for health and wellness apps. The Lancet Digital Health. 2022;4(2):80-82. doi:10.1016/s2589-7500(21)00273-9	(197)	1				1												1		1
Nederlands Centrum Jeugdgezondheid. Beeldbellen in de jeugdgezondheidszorg. 2023. https://www.jgzrichtlijnen.nl/praktijkmodule/praktijkmodule-beeldbellen-in-de-jeugdgezondheidszorg-2023/	(198)			1		1										1				
Nederlandse Zorgautoriteit. Wegwijzer bekostiging digitale zorg. 2025. https://puc.overheid.nl/nza/doc/PUC_11953_22/	(199)	1	1				1			1	1					1				
NELL. There's a new sheriff in town: Richtlijnen en kwaliteitsnormen voor AI-ontwikkeling. 2022. https://nell.eu/nieuws/blog-i-theres-a-new-sheriff-in-town-richtlijnen-en-kwaliteitsnormen-voor-ai-ontwikkeling	(200)	1	1	1			1				1		1	1						
Nieuwboer C. Oefenen met methodische online hulpverlening. Lectoraat Jeugd, Gezin en Samenleving. https://magazines.avans.nl/methodische-online-hulpverlening/oefenen-met-methodische-online-hulpverlening	(201)			1				1								1				
Nieuwboer C. Professionele online communicatie in zorg en welzijn. Lectoraat Jeugd, Gezin en Samenleving. https://magazines.avans.nl/online-communicatie-hoe-maak-je-de-klik/cover	(202)			1																
Nijs D, Drooghmans N. Mediawijsheidsnood: Nodendetectie en aanbevelingen betreffende het ondersteunen van mediawijsheid door professionals in de niet rechtstreeks toegankelijke hulpverlening. https://www.ucll.be/sites/default/files/documents/expertisecellen/eSocialWork/20190510_project_nodendetectiemediawijsheid.pdf	(203)	1		1	1	1			1					1						

Reference		Quality criteria of technology	Regularisaton	Quality criteria of professionals	Quality criteria of users	Quality criteria of organisations	Examples of governance	Examples of reports	Social work	Mental health care	Strict criteria	Growth path to quality	Meta thinking on quality	Flanders	Belgium	Netherlands	Europe	World, applicable	World, none applicable	Interpretation/ summary
Nordesjö K, Scaramuzzino G, Ulmestig R. The social worker-client relationship in the digital era: a configurative literature review. European Journal of Social Work. 2021;25(2):303-315. doi:10.1080/13691457.2021.1964445	(204)			1	1	1			1	1			1				1			
Nordesjö K, Scaramuzzino G. Digitalization, stress, and social worker–client relationships during the COVID-19 pandemic. Journal of Social Work. 2023;23(6):1080-1098. doi:10.1177/14680173231180309	(205)	1		1	1	1			1								1			
Open Web Application Security Project OWASP. OWASP Mobile Application Security. https://mas.owasp.org/#our-mission	(206)	1																1		
Opgroeien. Zeven B's als analysekader om drempels in kaart te brengen en weg te werken. Vlaanderen. https://www.opgroeien.be/kennis/themas/toegankelijkheid/zeven-bs-als-analysekader-om-drempels-kaart-te-brengen-en-weg-te-werken	(207)	1																		
Opgroeien. Kinderen, jongeren en gezinnen blended ondersteunen. Vlaanderen. 2022. https://www.opgroeien.be/kennis/toolbox/document-kinderen-jongeren-en-gezinnen-blended-ondersteunen-een-advies-over-visie-en-positie	(208)							1	1					1						
Opgroeien. Professioneel beeldbellen in welzijnswerk, sociaal werk en geestelijke gezondheidszorg. Vlaanderen. 2020. https://www.opgroeien.be/kennis/toolbox/draaiboek-professioneel-beeldbellen	(209)			1					1	1		1		1						
Opgroeien. Project Parkour. Vlaanderen. 2023. https://www.opgroeien.be/over-opgroeien/projecten/parkour	(210)	1		1	1									1						
Opgroeien. Quickscan voor onlinehulp in zorg en welzijn. Vlaanderen. 2018. https://www.opgroeien.be/kennis/toolbox/	(211)	1		1		1								1						

Reference		Quality criteria of technology	Regularisaton	Quality criteria of professionals	Quality criteria of users	Quality criteria of organisations	Examples of governance	Examples of reports	Social work	Mental health care	Strict criteria	Growth path to quality	Meta thinking on quality	Flanders	Belgium	Netherlands	Europe	World, applicable	World, none applicable	Interpretation/ summary
Orcha. The organisation for the review of care and health apps (ORCHA). 2023. https://orchahealth.com/	(212)	1				1				1	1							1		
Organisation for Economic Co-operation and Develoment (OECD). Technology and Innovation Outlook 2023: Enabling Transitions in Times of Disruption. OECD Publishing; 2023. doi:10.1787/0b55736e-en.	(213)	1	1				1					1					1	1	1	
Organisation for Economic Co-operation and Development (OECD). Health. 2023. https://www.oecd.org/en/topics/health.html/	(214)	1								1							1			
Osborne S. How does ISO 13485 compare to ISO 9001?. Horizon. 2022. https://www.extrahorizon.com/insights/blog-posts/how-does-iso-13485-compare-to-iso-9001	(215)	1				1												1		1
OWASP SAMM. Software Assurance Maturity Model. 2019. https://www.opensamm.org/	(216)	1				1													1	
OWASP. OWASP Top Ten. 2021. https://owasp.org/www-project-top-ten/	(217)	1									1	1	1				1			
Park SY, Nicksic Sigmon C, Boeldt D. A Framework for the Implementation of Digital Mental Health Interventions: The Importance of Feasibility and Acceptability Research. 2022. doi:10.7759/cureus.29329	(218)	1		1						1								1		
Patel O. EU AI Act Cheat Sheet [Infographic. International Association of Privacy Professionals; 2023. https://iapp.org/resources/article/eu-ai-act-cheat-sheet/	(219)	1	1								1						1			1
Patiëntenfederatie Nederland, Ieder(in), MIND Landelijk Platform Psychische Gezondheid, PGOsupport & IKONE. Samenwerken met patiënten: Digitale zorg die werkt – 7 aanbevelingen. Participatiekompas. 2018. https://participatiekompas.nl/media/20190930_handreiking-patie__ntenparticipatie-digitale-zorg_def.pdf	(220)	1	1	1	1	1			1							1				

Reference		Quality criteria of technology	Regularisaton	Quality criteria of professionals	Quality criteria of users	Quality criteria of organisations	Examples of governance	Examples of reports	Social work	Mental health care	Strict criteria	Growth path to quality	Meta thinking on quality	Flanders	Belgium	Netherlands	Europe	World, applicable	World, none applicable	Interpretation/ summary
Pattyn E, Bocklandt P. Cliëntreizen rond onlinehulp: achtergrond en aanpak. 2020. https://cdn.nimbu.io/s/0hkvgjb/channelentries/un0fsc3/files/Cli_ntreizen%20-%20achtergrond%20en%20aanpak%20-%20versie%2010%20februari%202020.pdf.	(221)	1							1	1		1		1						
Pharos. Quickscan digitale vaardigheden. https://www.pharos.nl/kennisbank/quickscan-digitale-vaardigheden-van-uw-patienten/	(222)			1	1						1					1				
Pharos. Toegankelijk en laagdrempelig beeldbellen in de zorg. https://www.pharos.nl/toegankelijk-laagdrempelig-beeldbellen-in-de-zorg/	(223)			1												1				
Pijpers R. Alles wat u moet weten over 21e eeuwse vaardigheden. Kennisnet. 2017. https://www.kennisnet.nl/artikel/alles-wat-je-moet-weten-over-21e-eeuwse-vaardigheden/	(224)			1												1				
Pote H, Moulton-Perkins A, Holloway-Biddle C. Competence framework for digital clinical practice: Psychological practitioners. British Psychological Society, Division of Clinical Psychology, Digital Healthcare Committee. Published online 2020. https://digitalhealthskills.com/digitalcompetencies	(225)			1			1		1	1	1								1	
Pote H, Rees A, Holloway-Biddle C, Griffith E. Workforce challenges in digital health implementation: How are clinical psychology training programmes developing digital competences? In: DIGITAL HEALTH. 2021. doi:10.1177/2055207620985396	(226)			1		1				1										
Reixach E, Andrés E, Sallent Ribes J, et al. Measuring the digital skills of Catalan health care professionals as a key step toward a strategic training plan: Digital competence test validation study. Journal of Medical Internet Research. 2022;24(11). doi:10.2196/38347	(227)			1			1										1			

Reference		Quality criteria of technology	Regularisaton	Quality criteria of professionals	Quality criteria of users	Quality criteria of organisations	Examples of governance	Examples of reports	Social work	Mental health care	Strict criteria	Growth path to quality	Meta thinking on quality	Flanders	Belgium	Netherlands	Europe	World, applicable	World, none applicable	Interpretation/ summary
Richardson MX, Landerdahl Stridsberg S, Wamala Andersson S. Evidence-related requirements in Swedish public sector procurement of health and welfare technologies – a systematic review. BMC Health Services Research. 2022;22(1). doi:10.1186/s12913-022-07723-x	(228)	1																		
Rijksinstituut voor ziekte- en invaliditeitsverzekering (RIZIV). Zorg op afstand: telemonitoring en therapiebegeleiding bij chronisch hartfalen. RIZIV; 2024. https://www.riziv.fgov.be/nl/professionals/verzorgingsinstellingen-en-diensten/ziekenhuizen/verzorging-in-ziekenhuizen/zorg-op-afstand-telemonitoring-en-therapiebegeleiding-bij-chronisch-hartfalen	(229)						1								1					
RIZIV. eGezondheid. 2022. Belgium. https://www.riziv.fgov.be/nl/thema-s/egezondheid	(230)	1	1			1	1			1	1	1			1					
Roberts CA, Smith KC, Sherman AK. Comparison of Online and Face-to-Face Parent Education for Children with Autism and Sleep Problems. Journal of Autism and Developmental Disorders. 2018;49(4):1410-1422. doi:10.1007/s10803-018-3832-2	(231)	1		1	1	1				1								1		
S.E.R.V. Artificiële intelligentie: Internationale verkenning van de sociaal-economische impact. 2021. https://www.serv.be/sites/default/files/documenten/SERV_20210208_Informatierapport_AI_RAP.pdf	(232)						1							1						
Saelens S, Vandecasteele A, Vanhove T, Debruyne H, Bocklandt P. Optimail: Een nieuwe methodiek voor e-mailhulpverlening. Arteveldehogeschool. 2012. https://b9e8513d6a.clvaw-cdnwnd.com/22701d8eac43dd786f280edfdcf61659/200000155-97ea798e76/Leidraad%20e-maildienstverlening%20in%20opvoedingsondersteuning.pdf	(233)			1		1			1					1						

Reference		Quality criteria of technology	Regularisaton	Quality criteria of professionals	Quality criteria of users	Quality criteria of organisations	Examples of governance	Examples of reports	Social work	Mental health care	Strict criteria	Growth path to quality	Meta thinking on quality	Flanders	Belgium	Netherlands	Europe	World, applicable	World, none applicable	Interpretation/ summary
Safe On Web at Work. Cyberfundamentals important. 2023. https://atwork.safeonweb.be/tools-resources/cyberfundamentals-framework	(234)					1					1				1					
Safe On Web at Work. Cyberfundamentals basic. 2023. https://atwork.safeonweb.be/tools-resources/cyberfundamentals-framework	(235)					1					1				1					
Safe On Web at Work. Cyberfundamentals essentials. 2023. https://atwork.safeonweb.be/tools-resources/cyberfundamentals-framework	(236)					1					1				1					
Safe On Web at Work. Cyberfundamentals small. 2023. https://atwork.safeonweb.be/tools-resources/cyberfundamentals-framework	(237)					1					1				1					
Safe On Web at Work. Cybersecurity self-assessment. 2023. https://atwork.safeonweb.be/nl/tools-resources/self-assessment	(238)					1					1				1					
San Miguel L, Obyn C, Vinck I, Meester C, Jespers V, Pouppe C. Hoe digitale medische toepassingen evalueren met het oog op terugbetaling. 2023. https://kce.fgov.be/nl/hoe-digitale-medische-toepassingen-evalueren-met-het-oog-op-terugbetaling	(239)	1					1			1	1				1					
Schalken F, Obyn C, Vinck I, Meester C, Jespers V, Pouppe C. Handboek online hulpverlening. Bohn Stafleu van Loghum. 2013. https://www.bohnstafleuvanloghum.nl/product/handboek-online-hulpverlening	(240)	1	1	1	1	1	1		1	1	1	1				1				
Schotte C, Broeck N. De competenties van de Belgische klinisch psycholoog: integratie van het profiel in Advies. van de Hoge Gezondheidsraad en het CanMEDS-model VVKP. 2018;(9194). https://vvkp.be/sites/default/files/TKP%202018-01_04_Competenties.pdf	(241)			1						1				1						

Reference		Quality criteria of technology	Regularisaton	Quality criteria of professionals	Quality criteria of users	Quality criteria of organisations	Examples of governance	Examples of reports	Social work	Mental health care	Strict criteria	Growth path to quality	Meta thinking on quality	Flanders	Belgium	Netherlands	Europe	World, applicable	World, none applicable	Interpretation/ summary
Schueller SM, Torous J. Scaling evidence-based treatments through digital mental health. American Psychologist. 2020;75(8):1093-1104. doi:10.1037/amp0000654	(242)	1	1	1	1	1				1								1		
Silberman J, Wicks P, Patel S, et al. Rigorous and rapid evidence assessment in digital health with the evidence DEFINED framework. npj Digital Medicine. 2023;6(1). doi:10.1038/s41746-023-00836-5	(243)	1		1		1				1	1							1		
Smart Interface Design Patterns. (2023). Color contrast cheat sheet. https://smart-interface-design-patterns.com/articles/color-contrast-cheat-sheet/#wcag-21-level-aa-minimum-contrast-cheat-sheet	(244)	1									1							1		
SNOMED International. 2023. https://www.snomed.org/	(245)	1								1								1		
Sociaal Werk Nederland. Aan de slag met kwaliteit: Kwaliteitslabel Sterk Sociaal Werk. 2022. https://sociaalwerknederland.nl/wp-content/uploads/wpallimport/files/426c49eed5fc1361477b30522bb9bd6fe25dacf4.pdf	(246)					1			1							1				
Social Care Institute for Excellence. Digital capabilities for social workers. https://www.scie.org.uk/social-work/digital-capabilities/stakeholders	(247)			1																
Software and Systems Engineering Standards Committee of the IEEE Computer Society. IEEE Standard for Data Privacy Process (IEEE Std 7002-2022). IEEE. 2022. https://standards.ieee.org/ieee/7002/6898/	(248)	1																1		
Software and Systems Engineering Standards Committee of the IEEE Computer Society. IEEE Standard Model Process for Addressing Ethical Concerns during System Design (IEEE Std 7000-2021). IEEE. 2021. https://doi.org/10.1109/IEEESTD.2021.9536679	(249)	1																1		

Reference		Quality criteria of technology	Regularisaton	Quality criteria of professionals	Quality criteria of users	Quality criteria of organisations	Examples of governance	Examples of reports	Social work	Mental health care	Strict criteria	Growth path to quality	Meta thinking on quality	Flanders	Belgium	Netherlands	Europe	World, applicable	World, none applicable	Interpretation/ summary
Sponselee A, Til J den O, M. G, S., Metz S. V-model: technologiekwalificaties van mbo- en hbo-professionals in zorg en welzijn. Onderwijs en Gezondheidszorg. 2021. doi:10.24078/oeng.2021.4.127178	(250)			1		1			1	1	1	1	1			1				
Stad Gent. Jaarverslag e-inclusie Gent 2024. https://stad.gent/nl/samenleven-welzijn-gezondheid/nieuws-evenementen/jaarverslag-digitale-inclusie-gent-2024	(251)	1					1	1						1						
Steunpunt Welzijn, Volksgezondheid en Gezin. Technologische gezondheidsinnovaties Ontwikkeling van een ethisch evaluatiekader. Flanders Care. 2021. https://www.flanderscare.be/evenementen/uitnodiging-seminarie-ethische-aspecten-van-zorginnovatie	(252)	1	1			1	1			1				1						
Stichting Koninklijk Nederlands Normalisatie-instituut (NEN). NEN 7510:2017 – Informatiebeveiliging in de zorg – Eisen voor een managementsysteem (3e ed.). NEN. 2017. https://www.nen.nl/nen-7510-1-2017-nl-232492	(253)	1	1				1				1					1				
Stichting Kruispuntbank van de Sociale Zekerheid (KSZ BCSS). Informatieveiligheidsbeleid. https://www.ksz-bcss.fgov.be/nl/gegevensbescherming/informatieveiligheidsbeleid	(254)	1	1			1	1								1					
Stoyanov SR, Hides L, Kavanagh DJ, Zelenko O, Tjondronegoro D, Mani M. Mobile App Rating Scale: A New Tool for Assessing the Quality of Health Mobile Apps. JMIR MHealth and UHealth. 2015;3(1). doi:10.2196/mhealth.3422	(255)	1								1	1	1	1				1			

Reference		Quality criteria of technology	Regularisaton	Quality criteria of professionals	Quality criteria of users	Quality criteria of organisations	Examples of governance	Examples of reports	Social work	Mental health care	Strict criteria	Growth path to quality	Meta thinking on quality	Flanders	Belgium	Netherlands	Europe	World, applicable	World, none applicable	Interpretation/ summary
Suominen J, Veikkolainen P, Kaksonen R, Voutilainen M, Haverinen J, FinCCHTA. Comparison Report of Digi-HTA and CEN/ISO TS 82304-2:2021. Finnish Coordinating Center for Health Technology Assessment (FinCCHTA; 2023. https://oys.fi/fincchta/wp-content/uploads/sites/21/2023/02/comparison-report-of-digi-hta-and-cen-iso-ts-82304-2-2021.pdf	(256)	1	1														1			1
Thieme A, Belgrave D, Doherty G. Machine Learning in Mental Health. ACM Transactions on Computer-Human Interaction. 2020;27(5):1-53. doi:10.1145/3398069	(257)	1								1								1		1
Tirions M, Raeymaeckers P, Boxstaens J, Cornille A, Gibens S, Postma Y. #sociaalwerk. Leuven: Acco; 2019.	(258)			1					1					1						
Tongeren P, Bal C. Blijven vragen wat kwaliteit is. Ethische Perspectieven. 1998;8(4):311-315. doi:10.2143/epn.8.4.516892	(259)			1									1				1			
Tools RRI. RRI tools. 2022. https://rri-tools.eu/	(260)	1				1	1					1					1			
Trimbos-instituut. Vragenlijst Onlinehulpstempel. 2016. https://www.onlinehulpstempel.nl/files/vragenlijst.pdf	(261)	1				1	1			1	1					1				
U.S. Department of Health and Human Services. Health Insurance Portability and Accountability Act of 1996 (HIPAA). 2021. https://www.hhs.gov/hipaa/for-professionals/index.html	(262)	1	1																1	
U.S. Food and Drug Administration. Artificial Intelligence/Machine Learning (AI/ML)-based Software as a Medical Device (SaMD) Action Plan. 2021. https://www.fda.gov/news-events/press-announcements/fda-releases-artificial-intelligencemachine-learning-action-plan	(263)	1	1							1									1	

Reference		Quality criteria of technology	Regularisaton	Quality criteria of professionals	Quality criteria of users	Quality criteria of organisations	Examples of governance	Examples of reports	Social work	Mental health care	Strict criteria	Growth path to quality	Meta thinking on quality	Flanders	Belgium	Netherlands	Europe	World, applicable	World, none applicable	Interpretation/ summary
U.S. Food and Drug Administration. Clinical Decision Support Software Guidance for Industry and Food and Drug Administration Staff. 2022. https://www.fda.gov/regulatory-information/search-fda-guidance-documents/clinical-decision-support-software	(264)	1	1							1									1	1
U.S. Food and Drug Administration. Clinical decision support software: Guidance for industry and Food and Drug Administration staff. 2022. https://www.fda.gov/regulatory-information/search-fda-guidance-documents/clinical-decision-support-software	(265)	1								1									1	1
U.S. Food and Drug Administration. Global approach to Software as a Medical Device. 2022. https://www.fda.gov/medical-devices/software-medical-device-samd/global-approach-software-medical-device	(266)	1								1								1		1
U.S. Food and Drug Administration. Medical device safety and the 510(k) clearance process. 2023. https://www.fda.gov/medical-devices/510k-clearances/medical-device-safety-and-510k-clearance-process	(267)	1	1							1									1	1
U.S. Food and Drug Administration. Medical devices. 2023.https://www.fda.gov/medical-devices	(268)	1	1																1	
U.S. Food and Drug Administration. Software as a Medical Device (SaMD). 2018. https://www.fda.gov/medical-devices/digital-health-center-excellence/software-medical-device-samd	(269)	1	1							1									1	1
Valentine L, D'Alfonso S, Lederman R. Recommender systems for mental health apps: advantages and ethical challenges. AI & SOCIETY. 2022;38(4):1627-1638. doi:10.1007/s00146-021-01322-w	(270)	1								1								1		

Reference		Quality criteria of technology	Regularisaton	Quality criteria of professionals	Quality criteria of users	Quality criteria of organisations	Examples of governance	Examples of reports	Social work	Mental health care	Strict criteria	Growth path to quality	Meta thinking on quality	Flanders	Belgium	Netherlands	Europe	World, applicable	World, none applicable	Interpretation/ summary
Van Bogaert M. 101 schrijftips die je moest missen op school. Lannoo Campus. 2022. https://www.lannoo.be/nl/101-schrijftips-die-je-moest-missen-op-school	(271)			1		1														
Van Gucht K. Guidelines for ehealth applications. imec; 2021.	(272)	1								1	1			1						
Van Smeden M, Moons C, Hooft L, Kant I, Os H, Cha	(273)	1	1	1	1	1										1				1
Vandemeulebroucke T, Mertens E, Dernier Y, Gastmans C. Technologische gezondheidsinnovaties: Ontwikkeling van een ethisch evaluatiekader. In: Steunpunt Welzijn, Volksgezondheid en Gezin. 2021. https://cdn.nimbu.io/s/5s8z9pq/channelentries/e9e9sdv/files/2021_14_Rapport_62_SWVG_EF47_2__Ethiek.pdf?be18f3m	(274)	1					1				1	1		1						
Vandooren T. Maar wij hebben toch geen klanten: Klantgericht organiseren in social profit. Owl Press. 2023. https://www.borgerhoff-lamberigts.be/owl-press/shop/boeken/maar-wij-hebben-toch-geen-klanten	(275)					1								1						
Verplancke J, Bocklandt P. Ready to blend?! Is jouw cliënt klaar voor een blended traject? Checklist voor sociale professionals en cliënten om samen blended te werken. Arteveldehogeschool – Expertiselijn Mens, Samenleving & Digitalisering. 2023. https://b9e8513d6a.clvaw-cdnwnd.com/22701d8eac43dd786f280edfdcf61659/200001061-c6040c6042/Ready%20to%20blend%20-%2018%20oktober%202023.pdf	(276)				1	1			1					1						
Vitalink. 2023. https://www.vitalink.be/	(277)	1	1							1	1			1			1			

Reference		Quality criteria of technology	Regularisaton	Quality criteria of professionals	Quality criteria of users	Quality criteria of organisations	Examples of governance	Examples of reports	Social work	Mental health care	Strict criteria	Growth path to quality	Meta thinking on quality	Flanders	Belgium	Netherlands	Europe	World, applicable	World, none applicable	Interpretation/ summary
Vlaams Agentschap Innoveren & Ondernemen (VLAIO). digitalisering cybersecurity.2023 https://www.vlaio.be/nl/begeleiding-advies/digitalisering/cybersecurity?utm_campaign=%5BPCMN%5D%20CyberStart-NL&utm_medium=email&_hsmi=80113415&_hsenc=p2ANqtz-_5b_e0QR1qjbeSZFHxQvnkXR8NY0XkFZ-nVWrUib5H6MrUrWrRQ5QFqZ6I_edGrsc9S-84EJY87aRJNMYfmpQt-WIN4MJ6JA913CEHMv65VQ5m0WA&utm_content=80113415&utm_source=hs_email	(278)	1				1								1						1
Vlaams expertisecentrum toegankelijkheid. Bestuursdecreet: omzetting Europese richtlijn voor de toegankelijkheid van de websites en mobiele applicaties van overheidsinstanties. Vlaanderen. 2018. https://www.vlaanderen.be/inter/toolbox-toegankelijke-steden-en-gemeenten/algemeen-bestuur-dienstverlening-en-communicatie/digitale-toegankelijkheid/bestuursdecreet-omzetting-europese-richtlijn-voor-de-toegankelijkheid-van-de-websites-en-mobiele-applicaties-van-overheidsinstanties	(279)	1	1				1				1			1	1		1			
Vlaams expertisecentrum toegankelijkheid. De Richtlijnen voor Toegankelijkheid van Webcontent (WCAG) in begrijpelijke taal. Vlaanderen. https://www.vlaanderen.be/inter/toolbox-toegankelijke-steden-en-gemeenten/algemeen-bestuur-dienstverlening-en-communicatie/digitale-toegankelijkheid/de-richtlijnen-voor-toegankelijkheid-van-webcontent-wcag-in-begrijpelijke-taal	(280)	1												1						1

Reference		Quality criteria of technology	Regularisaton	Quality criteria of professionals	Quality criteria of users	Quality criteria of organisations	Examples of governance	Examples of reports	Social work	Mental health care	Strict criteria	Growth path to quality	Meta thinking on quality	Flanders	Belgium	Netherlands	Europe	World, applicable	World, none applicable	Interpretation/ summary
Vlaams expertisecentrum toegankelijkheid. Toegankelijkheidsverklaring. Vlaanderen. https://www.vlaanderen.be/inter/toolbox-toegankelijke-steden-en-gemeenten/algemeen-bestuur-dienstverlening-en-communicatie/digitale-toegankelijkheid/toegankelijkheidsverklaring	(281)	1	1				1				1			1						1
Vlaams expertisecentrum toegankelijkheid. Waarderingsplatform: deel uw ervaring met zorg en ondersteuning. Departement Zorg en Gezondheid. https://www.departementzorg.be/nl/waarderingsplatform-deel-uw-ervaring-met-zorg-en-ondersteuning	(282)					1	1							1						
Vlaams Netwerk tegen Armoede. 24 speerpunten voor een structureel armoedebeleid in. 2023. doi:https://www.netwerktegenarmoede.be/nl/nieuws/2023/memorandum-van-het-vlaams-netwerk-tegen-armoede-verkiezingen-2024	(283)						1							1						
Vlaamse Kwalificatiestructuur. Beroepskwalificatiedossier klinisch psycholoog. https://app.akov.be/pls/pakov/f?p=VLAAMSE_KWALIFICATIESTRUCTUUR:2::::2:P2_BK_DOSSIER_ID,P2_BKD_PROCES_ID,P2_DOCUMENT_TYPE_ID,P2_PROCES_STAP_ID,P2_FORMAT:6321,7941,82,6,PDF	(284)			1						1				1						
Voka. Digitale Quickscan. https://www.voka.be/digitale-quickscan	(285)					1								1						
Vuorikari R, Kluzer S, Punie Y. DigComp 2.2: The Digital Competence Framework for Citizens. Publications Office of the European Union; 2022. https://publications.jrc.ec.europa.eu/repository/handle/JRC128415	(286)			1	1							1					1			
Watson L. WCAG primer. 2020. https://tetralogical.com/blog/2020/04/10/wcag-primer/	(287)	1									1							1		1

Reference		Quality criteria of technology	Regularisaton	Quality criteria of professionals	Quality criteria of users	Quality criteria of organisations	Examples of governance	Examples of reports	Social work	Mental health care	Strict criteria	Growth path to quality	Meta thinking on quality	Flanders	Belgium	Netherlands	Europe	World, applicable	World, none applicable	Interpretation/ summary
Wentzel J, Vaart R, Bohlmeijer ET, Gemert-Pijnen JEWC. Mixing Online and Face-to-Face Therapy: How to Benefit From Blended Care in Mental Health Care. JMIR Mental Health. 2016;3(1). doi:10.2196/mental.4534	(288)			1	1													1		
Witte N, Daele T. UTAUT-vragenlijsten. Onderzoeksgroep Zorg en Welzijn. 2017. https://thomasmore.be/nl/zorg-en-welzijn-mens-en-welzijn/vragenlijst-utaut	(289)			1	1	1								1						
World Health Organization. Global Initiative on Digital Health. https://www.who.int/initiatives/global-initiative-on-digital-health	(290)	1	1			1	1											1		
World Health Organization. National eHealth strategy toolkit. 2012. https://www.who.int/publications/i/item/national-ehealth-strategy-toolkit	(291)	1					1			1	1	1						1		
World Health Organization. Recommendations on digital interventions for health systems strengthening. 2019. https://www.who.int/publications/i/item/9789241550505	(292)	1					1											1		
World Health Organization. WHO calls for safe and ethical AI for health. 2023. https://www.who.int/news/item/16-05-2023-who-calls-for-safe-and-ethical-ai-for-health#:~:text=The%206%20core%20principles%20identified%20by%20WHO%20are%3A,%286%29%20promote%20AI%20that%20is%20responsive%20and%20sustainable	(293)	1	1				1			1								1		
World Health Organization. WHO outlines considerations for regulation of artificial intelligence for health. 2023. https://www.who.int/news/item/19-10-2023-who-outlines-considerations-for-regulation-of-artificial-intelligence-for-health	(294)	1	1			1				1								1		

Reference		Quality criteria of technology	Regularisaton	Quality criteria of professionals	Quality criteria of users	Quality criteria of organisations	Examples of governance	Examples of reports	Social work	Mental health care	Strict criteria	Growth path to quality	Meta thinking on quality	Flanders	Belgium	Netherlands	Europe	World, applicable	World, none applicable	Interpretation/ summary
World Health Organization. WHO releases AI ethics and governance guidance for large multi-modal models. 2024. https://www.who.int/news/item/18-01-2024-who-releases-ai-ethics-and-governance-guidance-for-large-multi-modal-models	(295)	1	1				1			1								1		
World Wide Web Consortium (W3C). W3C Accessibility Standards Overview. Web Accessibility Initiative (WAI). 2024. https://www.w3.org/WAI/standards-guidelines/	(296)	1	1								1	1	1					1		
World Wide Web Consortium (W3C). Web Content Accessibility Guidelines (WCAG) 2.2. 2023. https://www.w3.org/TR/WCAG22/	(297)	1									1							1		
Zantvoord J. Eindrapport onderzoek Digitale Vaardigheden. 2020. https://utrechtzorg.net/media/pages/nieuws/programma-digitaal-vaardig-levert-waardevolle-aanbevelingen-voor-zorg-en-welzijn/15fafdb5fa-1655902278/digitale-vaardigheden-onderzoeksrapport-regio-utrecht_-juli-2020.pdf	(298)			1		1								1						
Zemaitaityte I, Bardauskiene R, Pivoriene J, Katkonienė A. Digital competences of future social workers: the art of education in uncertain times. Social Work Education. 2023;43(4):1078-1091. doi:10.1080/02615479.2022.2164269	(299)			1													1			

Reference		Quality criteria of technology	Regularisaton	Quality criteria of professionals	Quality criteria of users	Quality criteria of organisations	Examples of governance	Examples of reports	Social work	Mental health care	Strict criteria	Growth path to quality	Meta thinking on quality	Flanders	Belgium	Netherlands	Europe	World, applicable	World, none applicable	Interpretation/ summary
Zhu H, Andersen ST. Digital competence in social work practice and education: experiences from Norway. Nordic Social Work Research. 2021;12(5):823-838. doi:10.1080/2156857x.2021.1899967	(300)			1																
Ziegler M, Iliescu D. Measurement does not take place in a legal vacuum: Ideas regarding Regulation (EU) 2017/745 of the European Parliament and of the Council on Medical Devices. European Journal of Psychological Assessment. 2023;39(2):79-84. doi:10.1027/1015-5759/a000764	(301)	1	1							1	1						1			1
Zorginstituut Nederland. Evaluatie van eHealth technologie in de context van beleid. Zorg. 2017. https://www.zorgvoorinnoveren.nl/.uc/fb134d21601020dd1ae00fd5ed102b797be3b9b6c082500/Evaluatie-van-eHealth-technologie-web.pdf	(302)	1				1	1									1				
Zorgverzekeraars Nederland. Leidraad applicaties en algoritmes in de zorg. https://www.zn.nl/dossier/digitalisering/	(303)	1					1									1				

References

1. 8 Caring Technology Principles. Eight guiding principles for caring technology. King Baudouin Foundation; 2021. https://www.fondsdanieldeconinck.be/en/initiative/caring-technology/
2. Aaltonen ES. Client-oriented quality assessment within municipal social services. Int J Soc Welf. 1999;8(2):131-142. doi:10.1111/1468-2397.00074
3. Abrishami P, Boer A, Horstman K. How can we assess the value of complex medical innovations in practice? Expert Review of Pharmacoeconomics & Outcomes Research. 2015;15(3):369-371. doi:10.1586/14737167.2015.1037834
4. Agarwal S, LeFevre AE, Lee J, et al. Guidelines for reporting of health interventions using mobile phones: mobile health (mHealth) evidence reporting and assessment (mERA) checklist. BMJ. 2016. doi:10.1136/bmj.i1174
5. Agentschap Digitaal Vlaanderen. Digitale dienst zelfevaluatiedocument. 2022. https://www.vlaanderen.be/digitaal-vlaanderen/vlaamse-digitale-strategie/digitale-dienstverleningsstrategie
6. Agentschap Digitaal Vlaanderen. Scores rekenblad digitale dienst. 2022. https://www.vlaanderen.be/digitaal-vlaanderen/vlaamse-digitale-strategie/digitale-dienstverleningsstrategie
7. Agoria en beMedTech. Validatiepiramide m-health Belgium. https://mhealthbelgium.be/nl/validatiepiramide
8. Agoria en cyberstart. Cybersecurity woordenboek. https://www.agoria.be/cyberstart/cybersecurity-woordenboek?utm_campaign=%5BPCMN%5D%20CyberStart-NL&utm_medium=email&_hsmi=80113415&_hsenc=p2ANqtz-8UoQ0HzPMVlKRdo0VEaNvAJPi_89orDK9P8XuznGSkKtyn9dhKQbibRCwCOuw0VVmIr-69uPTfw1i2759GLjEkBRHG2TtcJLbiSG7OZLnrPw70yzY&utm_content=80113415&utm_source=hs_email
9. Agoria. Software supply chain risicobeheersing. 2023. https://www.agoria.be/cyberstart/buyers-guide-software-supply-chain-risicobeheersing?utm_campaign=%5BPCMN%5D%20CyberStart-NL&utm_medium=email&_hsmi=80113415&_hsenc=p2ANqtz-9PbjmiY7Z279uSc5HdVt9d7nFTEHp6q7hLGlosNLArTVICi6EvWTlLuZX6XqP3w4UB_nYm0Jz8C38R_ginnjvviHPYpI4XfeB6PyylVuCheRDD6a4&utm_content=80113415&utm_source=hs_email
10. Ahmed S, Trimmer C, Khan W, et al. A mixed methods analysis of existing assessment and evaluation tools (AETs) for mental health applications. Frontiers in Public Health. 2024;12. doi:10.3389/fpubh.2024.1196491
11. AI4Europe. Ethic Assessment Tools. 2019. https://www.ai4europe.eu/Ethics-Assessment-Tools-ALTAI
12. Almathami HKY, Win KT, Vlahu-Gjorgievska E. Barriers and facilitators of online consultations at patients' homes. JMIR Publications. 2020;22(2). doi:10.2196/16407
13. AMA. Validating digital health innovations. 2023. https://www.ama-assn.org/practice-management/digital/validating-digital-health-innovations
14. APA. Health Information Technology and Telepsychology. 2016. https://www.apaservices.org/practice/legal/technology
15. APA. The app evaluation model. https://www.psychiatry.org/psychiatrists/practice/mental-health-apps/the-app-evaluation-model

16. Arteveldehogeschool. Swipe: Sterk Sociaal Werk Inspirerende Praktijken Digitalisering. 2022. https://www.arteveldehogeschool.be/nl/onderzoek/projecten/swipe-sterk-sociaal-werk-inspirerende-praktijken-digitalisering
17. Artsenfederatie KNMG. Alles wat u moet weten over videoconsulten met patiënten. 2021. https://www.knmg.nl/actualiteit-opinie/nieuws/nieuwsbericht/alles-wat-u-moet-weten-over-videoconsulten-met-patienten
18. ASQ. ISO 9001. 2024. https://asq.org/quality-resources/iso-9001
19. Australian Commission on Safety and Quality in Health Care. National Safety and Quality Digital Mental Health Standards. 2020. https://www.safetyandquality.gov.au/standards/national-safety-and-quality-digital-mental-health-standards
20. Australian Government Department of Health and Aged Care. Australia: Therapeutic Goods Administration (TGA) . https://www.tga.gov.au/
21. Australian Medical Association. National mHealth applications assessment framework. 2021. https://www.ama.com.au/sites/default/files/2021-11/AMA%20Submission%20to%20mHealth%20applications%20assessment%20framework_Final.pdf
22. Balcombe L, Leo D. Human-Computer Interaction in Digital Mental Health. Informatics. 2022;9(1). doi:10.3390/informatics9010014
23. België. Wet van 26 april 2024 houdende vaststelling van een kader voor de cyberbeveiliging van netwerk- en informatiesystemen van algemeen belang voor de openbare veiligheid (NIS2-wet). Belgisch Staatsblad. 2024. http://www.ejustice.just.fgov.be/eli/wet/2024/04/26/2024202344/justel
24. BelRAI. Wat is BelRAI - MyBelRAI? BelRAI. https://www.belrai.org/nl/wat-belrai-mybelrai-0
25. Blindenzorg Licht en Liefde vzw. Toegankelijkheidsmonitor 2020. 2020. https://toegankelijkheidsmonitor.be/2020.html
26. Blumenthal D. Part 1: Quality of care–what is it? The New England journal of medicine. 1996;335(12):891-894. doi:10.1056/NEJM199609193351213
27. Bocklandt P, Adriaensens E, Zitter M, Sep H, Vandecasteele A. Bouwstenen Vlaams actieplan onlinehulp. Arteveldehogeschool. 2014. https://www.onlinehulp-arteveldehogeschool.be/onlinehulponderzoek/vlaams-actieplan-onlinehulp/
28. Bocklandt P, Beelen S, Claeys H, Custers S, Mindermann O, Daele T. Onlinehulp-Vlaanderen. Screeningscriteria voor apps en websites in onlinehulp-apps.be. 2022. https://www.onlinehulp-apps.be/screeningskader
29. Bocklandt P, Verplancke J, Faelens L, et al. Een kader voor digitale competenties van sociaal-agogische professionals. 2025. https://cdn.nimbu.io/s/0hkvgjb/assets/1737548451710/20250122_p_een-kader-voor-digitale-competenties-van-sociale-professionals.pdf
30. Bocklandt P. Medewerkersvertrouwen in onlinehulp. 2020. https://cdn.nimbu.io/s/0hkvgjb/channelentries/ywob7v1/files/medewerkersvertrouwen%20in%20onlinehulp%20-%208%20januari%202020.pdf?moerema=&dl=1
31. Bocklandt P. Professioneel beeldbellen in welzijnswerk, sociaal werk & geestelijke gezondheidszorg: Handvatten voor systematisch en doelgericht videochatten. Arteveldehogeschool. 2021. https://cdn.nimbu.io/s/0hkvgjb/channelentries/ulaif2b/files/methodiek%20beeldbellen%20in%20welzijnswerk%20sociaal%20werk%20en%20geestelijke%20gezondheidszorg%20-%20versie%204%20januari%202021.pdf
32. Boutens L. Online toepassingen rond de alcohol-, drug- en gokproblematiek - inspiratie voor intermediairs. Vlaams Expertisecentrum Alcohol en Andere Drugs.

2016. https://vad.be/catalogus/online-toepassingen-rond-de-alcohol-drug-en-gokproblematiek/
33. Boxstaens J, Reynders B. We creëren een kwaliteitskader waarin iedereen naar waarde wordt geschat, op elk niveau". 2024. https://www.zorgneticuro.be/artikel/we-creeren-een-kwaliteitskader-waarin-iedereen-naar-waarde-wordt-geschat-op-elk-niveau
34. Braeckel L. Blended hulp reikt online en offline de hand. Welzijns- en Gezondheidsmagazine. 2019;144. doi:https://publicaties.vlaanderen.be/view-file/1472
35. Broekhuis M, Velsen L, Hermens H. Assessing usability of eHealth technology. International Journal of Medical Informatics. 2019;128:24-31. doi:10.1016/j.ijmedinf.2019.05.001
36. Brown P, Prest B, Miles P, Rossi V. The development of National Safety and Quality Digital Mental Health Standards. Australasian Psychiatry. 2021;30(2):154-157. doi:10.1177/10398562211042361
37. Business, Economy, Euro. CE markering. European Union. https://europa.eu/youreurope/business/product-requirements/labels-markings/ce-marking/index_nl.htm#shortcut-0
38. Byrne-Haber S. Giving a Damn about Accessibility: A Candid and Practical Handbook for Designers. UX Collective; 2021. https://www.accessibility.uxdesign.cc/
39. Cambell A, Adams C, Montgomery RB, Cooper M, Kirkpatrick A. Web Content Accessibility Guidelines (WCAG) 2.2. W3C Recommendation. 2024. https://www.w3.org/TR/WCAG22/
40. CCLAB. ETSI EN 303 645 Infographics for Consumer IoT devices. 2021. https://www.cclab.com/downloads/etsi-infographics
41. Center for Internet Security. Creating Confidence in the Connected World. 2023. https://www.cisecurity.org/
42. Centre for Cybersecurity Belgium. Centre for Cybersecurity Belgium. https://ccb.belgium.be/
43. Centre for Cybersecurity Belgium. Cyber Emergency Response Team. 2023. https://ccb.belgium.be/nl/cert
44. Centre for Cybersecurity Belgium. De NIS2-richtlijn : wat betekent dit voor mijn organisatie? 2023. https://ccb.belgium.be/nl/de-nis2-richtlijn-wat-betekent-dit-voor-mijn-organisatie#_Toc127800317
45. Centre for Cybersecurity Belgium. Service Definition Document—Federal Cyber Emergency Response Team CERT.be. Version. 2024;4(00). https://ccb.belgium.be/cert/service-definition-document
46. Connolly SL, Kuhn E, Possemato K, Torous J. Digital Clinics and Mobile Technology Implementation for Mental Health Care. Current Psychiatry Reports. 2021;23(7). doi:10.1007/s11920-021-01254-8
47. Consortium WWW. Web Content Accessibility Guidelines (WCAG) 2.1. 2018. https://www.w3.org/TR/WCAG21/
48. Cook L L ,Zschomler D. Virtual home visits during the COVID-19 pandemic: Social workers' perspectives. Perspectives. 2020;32(5). doi:10.1080/09503153.2020.1836142
49. Corrêa NK, Galvão C, Santos JW, et al. Worldwide AI ethics: A review of 200 guidelines and recommendations for AI governance. Patterns. 2023;4(10). doi:10.1016/j.patter.2023.100857
50. Cox D. They thought they were doing good but it made people worse': why mental health apps are under scrutiny. The Guardian. 2024. doi:https://www.theguardian.com/society/2024/feb/04/they-thought-they-were-doing-good-but-it-made-people-worse-why-mental-health-apps-are-under-scrutiny

51. Custers S, Drooghmans N, Nijs D. Digitale inclusie in tijden van corona. Noden uit het welzijnswerk. UCLL Research & Expertise. 2020. https://www.ucll.be/sites/default/files/documents/ucll_-_onderzoeksrapport_nodenbevraging_digitale_inclusie_in_tijden_van_.pdf
52. Cwikel J, Friedmann E. E-therapy and social work practice: Benefits, barriers, and training. International Social Work. 2019;63(6):730-745. doi:10.1177/0020872819847747
53. Cybersecurity Bites. Cybersecurity Bites. 2023. https://cybersecurity-bites.be/
54. D'Alfonso S. AI in mental health. Current Opinion in Psychology. 2020;36:112-117. doi:10.1016/j.copsyc.2020.04.005
55. Daele T, Karekla M, Kassianos AP, et al. Recommendations for policy and practice of telepsychotherapy and e-mental health in Europe and beyond. Journal of Psychotherapy Integration. 2020;30(2):160-173. doi:10.1037/int0000218
56. Damschroder LJ, Aron DC, Keith RE, Kirsh SR, Alexander JA, Lowery JC. Fostering implementation of health services research findings into practice: A consolidated framework for advancing implementation science. Implementation Science. 2009;4. doi:10.1186/1748-5908-4-50
57. Damschroder LJ, Reardon CM, Widerquist MAO, Lowery J. The updated Consolidated Framework for Implementation Research based on user feedback. Implementation Science. 2022;17(1). doi:10.1186/s13012-022-01245-0
58. de Hond AAH, Leeuwenberg AM, Hooft L, et al. Guidelines and quality criteria for artificial intelligence-based prediction models in healthcare: a scoping review. Npj Digital Medicine. 2022;5(1). doi:10.1038/s41746-021-00549-7
59. De Jaegere E. Ethische richtlijnen voor 'technology-based' suïcidepreventie programma's. https://www.flanderscare.be/sites/default/files/media/ethiek_FCCOH.pdf
60. De Marez L, Georges A, Sevenhant R, Devos E. Imec.digimeter.2024. In: Digitale trends in Vlaanderen. Imec. 2025. doi:https://www.imec.be/nl/kennisuitwisseling/techmeters/digimeter/imecdigimeter-2024
61. Departement Werk, Economie, Wetenschap, Innovatie en Sociale Economie. Bletchley-verklaring: eerste internationale verklaring over veilige ontwikkeling van artificiële intelligentie (AI). Vlaanderen. 2023. https://www.ewi-vlaanderen.be/nieuws/bletchley-verklaring-eerste-internationale-verklaring-over-veilige-ontwikkeling-van?utm_medium=email&utm_campaign=Nieuwsbrief%20EWI%20%2014%20november%202023&utm_content=Nieuwsbrief%20EWI%20%2014%20november%202023+CID_95d794573cb316353d3f5852e1df6451&utm_source=Email%20marketing%20software&utm_term=Bletchley-verklaring%20eerste%20internationale%20verklaring%20over%20veilige%20ontwikkeling%20van%20artificile%20intelligentie%20AI
62. Departement Werk, Economie, Wetenschap, Innovatie en Sociale Economie. The Flemish AI Plan. https://www.ewi-vlaanderen.be/en/flemish-ai-plan
63. Departement Werk, Economie, Wetenschap, Innovatie en Sociale Economie. Transities mogelijk maken in tijden van disruptie. Vlaanderen. 2023. https://www.ewi-vlaanderen.be/nieuws/transities-mogelijk-maken-tijden-van-disruptie
64. Departement Zorg. Alivia: uw digitaal zorg- en ondersteuningsplan. Vlaanderen. https://www.zorg-en-gezondheid.be/alivia-uw-digitaal-zorg-en-ondersteuningsplan
65. Departement Zorg. BelRAI in Vlaanderen. Vlaanderen. https://www.departementzorg.be/nl/belrai-vlaanderen

66. Departement Zorg. Vlaams Agentschap voor de Samenwerking rond Gegevensdeling tussen de Actoren in de Zorg (VASGAZ): wie of wat is het agentschap VASGAZ? 2017. https://www.departementwvg.be/wie-wat-het-agentschap
67. Department of Health, Disability and Ageing. Understanding regulation of software-based medical devices. Australian Government. 2022. https://www.tga.gov.au/how-we-regulate/manufacturing/medical-devices/manufacturer-guidance-specific-types-medical-devices/regulation-software-based-medical-devices
68. Developers Android. Core app quality. Android. https://developer.android.com/docs/quality-guidelines/core-app-quality#listing
69. Devlieghere J, Roose R, Evans T. Managing the electronic turn. European Journal of Social Work. 2019;23(5):767-778. doi:10.1080/13691457.2019.1582009
70. Digitaal Vlaanderen. Digitale dienstverleningsstrategie voor Vlaamse overheden. Vlaanderen. 2022. https://www.vlaanderen.be/digitaal-vlaanderen/vlaamse-digitale-strategie/digitale-dienstverleningsstrategie
71. Digitaal Vlaanderen. Meetinstrument digitale vaardigheden. Vlaanderen https://www.vlaanderen.be/samenleven/toegankelijkheid-e-inclusie/e-inclusie/meetinstrument-digitale-vaardigheden
72. Digitaal Vlaanderen. Vademecum bij gebruik aftoetsingsinstrument: Handleiding voor het aftoetsingsinstrument voor digitale dienstverlening. Vlaamse Overheid. 2023. https://www.vlaanderen.be/digitaal-vlaanderen/vlaamse-digitale-strategie/digitale-dienstverleningsstrategie
73. Digitaal Vlaanderen. Vlaamse strategie informatieveiligheid. https://www.vlaanderen.be/digitaal-vlaanderen/vlaamse-digitale-strategie/vlaamse-strategie-informatieveiligheid
74. Digitale Zorg Gids. Hulp bij digitale zorg. https://www.digitalezorggids.nl/hulp-bij-digitale-zorg/
75. Digitall. Wat is de Digital Inclusion bij Design Index? https://digitalinclusionindex.digitall.be/nl#:~:text=De%20Digital%20Inclusion%20by%20Design%20Index%20toont%20u%20hoe%20toegankelijk,zoals%20marketeers%2C%20productontwerpers%20en%20webdesigners
76. Digivaardig in de zorg. Zelfscan digitale vaardigheden welzijn. https://www.digivaardigindezorg.nl/welzijn-sociaal-werk/home/zelftest/#:~:text=Met%20de%20zelfscan%20digitale%20vaardigheden,geef%20zo%20zorgvuldig%20mogelijk%20antwoord.
77. Digivaardig in de zorg. Zo meet je de stand van digitale vaardigheden in jouw organisatie. 2019. https://www.digivaardigindezorg.nl/zo-meet-je-de-stand-van-de-digitale-vaardigheden-in-jouw-organisatie/
78. Drude KP, Maheu MM. The Need for Interprofessional Consensus about Telebehavioral Health Competencies and Education. J Technol Behav Sci. 2024;9(1):7-13. doi:10.1007/s41347-023-00369-4
79. EFQM. Model EFQM. 2019. https://efqm.org/the-efqm-model/
80. EHealth Tools Assessment. Assessment Instrument. https://ehealth-criteria-toolbox.net/assessment-instrument/
81. eHealth. eHealth Welcome Pack. Belgium. 2023. https://www.ehealth.fgov.be/ehealthplatform/nl
82. eHealth-platform. CoBRHA – Common Base Registry for HealthCare Actor. 2022. https://www.ehealth.fgov.be/ehealthplatform/nl/service-cobrha-common-base-registry-for-healthcare-actor
83. EIT Health. European Taskforce for Harmonised Evaluations of Digital Medical Devices (DMDs). https://eithealth.eu/external-collaborations/european-taskforce-for-harmonised-evaluations-of-digital-medical-devices-dmds/

84. EIT Health. Towards a European evaluation framework for digital medical devices (DMDs) in the European Union – Launch of a European taskforce. 2022. https://eithealth.eu/news-article/press-release-digital-medical-devices-launch-of-a-european-taskforce/
85. ETSI. Cyber security for consumer internet of things (ETSI EN 303 645 V2.1.1). 2020. https://www.etsi.org/deliver/etsi_en/303600_303699/303645/02.01.01_60/en_303645v020101p.pdf
86. EU Cloud COC. https://eucoc.cloud/en/home
87. European Commission, Directorate-General for Internal Market, Industry, Entrepreneurship and SMEs. Manufacturers – CE marking for goods in the EU internal market. https://single-market-economy.ec.europa.eu/single-market/goods/ce-marking/manufacturers_en
88. European Commission. Commission Welcomes Political Agreement on Cyber Resilience Act. European Union; 2023. https://ec.europa.eu/commission/presscorner/detail/en/ip_23_6168
89. European Commission. EU4Health programme 2021-2027 – a vision for a healthier European Union. 2021. https://health.ec.europa.eu/funding/eu4health-programme-2021-2027-vision-healthier-european-union_en#work-programmes
90. European Commission. The EU Cybersecurity Act. European Union. 2025. https://digital-strategy.ec.europa.eu/en/policies/cybersecurity-act
91. European Commission. The EU cybersecurity certification framework. European Union; 2025. https://digital-strategy.ec.europa.eu/en/policies/cybersecurity-certification-framework
92. European Data Protection Board. Guidelines on deceptive design patterns in social media platform interfaces. European Union. 2022. https://www.edpb.europa.eu/our-work-tools/our-documents/guidelines/guidelines-032022-deceptive-design-patterns-social-media_en
93. European Data Protection Board. Richtsnoeren inzake toestemming overeenkomstig. European Union. 2022. https://edpb.europa.eu/our-work-tools/our-documents/guidelines/guidelines-052020-consent-under-regulation-2016679_nl
94. European Parliament. Shaping the digital transformation: EU strategy explained. 2021. https://www.europarl.europa.eu/topics/en/article/20210414STO02010/shaping-the-digital-transformation-eu-strategy-explained
95. European Union. Directive (EU) 2016/1148 of the European Parliament and of the Council of 6 July 2016 concerning measures for a high common level of security of network and information systems across the Union. Off J Eur Union. 2016;L194:1-30. http://data.europa.eu/eli/dir/2016/1148/oj
96. European Union. Directive (EU) 2019/882 of the European Parliament and of the Council of 17 April 2019 on the accessibility requirements for products and services (European Accessibility Act). Official Journal of the European Union. 2019;L151:70–115. Available at: http://data.europa.eu/eli/dir/2019/882/oj. Accessed November 25, 2025.
97. European Union. Directive (EU) 2022/2555 of the European Parliament and of the Council of 14 December 2022 on measures for a high common level of cybersecurity across the Union, amending Regulation (EU) No 910/2014 and Directive (EU) 2018/1972, and repealing Directive (EU) 2016/1148 (NIS 2 Directive) [Directive]. Official Journal of the European Union. 2022; L 333, 80–152. http://data.europa.eu/eli/dir/2022/2555/oj
98. European Union. Regulation (EU) 2016/679 of the European Parliament and of the Council of 27 April 2016 on the protection of natural persons with regard to the processing of personal data and on the free movement of such data, and repealing

Directive 95/46/EC (General Data Protection Regulation) [Regulation]. Official Journal of the European Union. 2016; L 119, 1–88. https://eur-lex.europa.eu/eli/reg/2016/679/oj
99. European Union. Regulation (EU) 2017/745 of the European Parliament and of the Council of 5 April 2017 on medical devices, amending Directive 2001/83/EC, Regulation (EC) No 178/2002 and Regulation (EC) No 1223/2009, and repealing Council Directives 90/385/EEC and 93/42/EEC [Regulation]. Official Journal of the European Union. 2017; L 117, 1–175. https://eur-lex.europa.eu/legal-content/EN/TXT/?uri=CELEX%3A32017R0745
100. European Union. Regulation (EU) 2024/1689 of the European Parliament and of the Council of 13 June 2024 laying down harmonised rules on artificial intelligence. Official Journal of the European Union.2024; 1689, 1–144. http://data.europa.eu/eli/reg/2024/1689/oj
101. European Union. Regulation (EU) 2024/2847 of the European Parliament and of the Council of 23 October 2024 on horizontal cybersecurity requirements for products with digital elements and amending Regulations (EU) No 168/2013 and (EU) No 2019/1020 and Directive (EU) 2020/1828 (Cyber Resilience Act) [Regulation]. Official Journal of the European Union. 2024; L 2847, 1–66. http://data.europa.eu/eli/reg/2024/2847/oj
102. European Union. Regulation (EU) 2025/327 of the European Parliament and of the Council of 11 February 2025 on the European Health Data Space and amending Directive 2011/24/EU and Regulation (EU) 2024/2847. Off J Eur Union. 2025;L5:1-96. https://eur-lex.europa.eu/eli/reg/2025/327/oj
103. Extra Horizon. All you need to know about IEC 62304 compliant software development. 2023. https://www.extrahorizon.com/iec-62304-software-development-medical-devices
104. Eysenbach G, CONSORT-EHEALTH Group. Improving and Standardizing Evaluation Reports of Web-based and Mobile Health Interventions. Journal of Medical Internet Research. 2011;13(4). doi:10.2196/jmir.1923
105. Faure L, Brotcorne P, Vendramin P, Mariën I. Barometer Digitale Inclusie 2022. Koning Boudewijnstichting. 2022. doi:https://kbs-frb.be/nl/barometer-digitale-inclusie-2022
106. Federaal Agentschap voor Geneesmiddelen en Gezondheidsproducten (FAGG). Medische hulpmiddelen en hun hulpstukken. FAGG. 2025. https://www.fagg.be/nl/MENSELIJK_gebruik/gezondheidsproducten/medische_hulpmiddelen_hulpstukken
107. Federaal Kenniscentrum voor de Gezondheidszorg KCE. Moeten medische apps worden vergoed? En zo ja, welke? 2023. https://kce.fgov.be/en/kce-press-release-kce-reports-362-evaluation-of-digital-medical-technologies
108. Federal Institute for Drugs and Medical Devices. Digital Health Applications (DiGA). 2020. https://www.bfarm.de/EN/Medical-devices/Tasks/DiGA-and-DiPA/Digital-Health-Applications/_node.html
109. Federale Overheid. Wet van 7 april 2019 tot vaststelling van een kader voor de beveiliging van netwerk- en informatiesystemen van algemeen belang voor de openbare veiligheid. Belgisch Staatsblad. Published online 2019. https://economie.fgov.be/nl/legislation/wet-van-7-april-2019-tot
110. Federale Overheidsdienst Volksgezondheid. Actieplan e-Gezondheid 2022-2024. 2022. https://www.health.belgium.be/sites/default/files/uploads/fields/fpshealth_theme_file/actieplan_egezondheid_2022-2024_protocolakkoord.pdf

111. Fransen J. Methodiek online hulpverlening voor schoolmaatschappelijk werk. Hogeschool Inholland; 2011.
112. Frederix P, Drooghmans N, Vandries T. Handelingskader "Omgaan met online risico's vanuit het denkkader emotionele ontwikkeling.". https://research-expertise.ucll.be/sites/default/files/2021-12/Handelingskader%20Final%20Layout%20V3%281%29.pdf
113. Friedman V. How to make a strong case for accessibility. Smart Interface Design Patterns. 2023. https://smart-interface-design-patterns.com/articles/accessibility-strong-case/
114. Friedman V. Mobile accessibility target sizes. Smart Interface Design Patterns. 2023. https://smart-interface-design-patterns.com/articles/accessible-tap-target-sizes/
115. Gaglio B, Shoup JA, Glasgow RE. The RE-AIM framework: a systematic review of use over time. Am J Public Health. 2013;103(6):e38–e46. PMID:23597377 https://doi.org/10.2105/ajph.2013.301299
116. Gegevensbeschermingsautoriteit. De GBA publiceert een checklist voor het correcte gebruik van cookies. 2023. https://www.gegevensbeschermingsautoriteit.be/burger/nieuws/2023/10/20/de-gba-publiceert-een-checklist-voor-het-correcte-gebruik-van-cookies
117. GGD AppStore. De testmethode: GGD AppStore methodiek. 2016. https://www.ggdappstore.nl/Appstore/Testmethode
118. GGZ Standaarden. Digitale zorg. 2021. https://www.ggzstandaarden.nl/zorgstandaarden/ehealth/introductie
119. Goubin E. Communicatie als bindkracht: Tien communicatie-uitdagingen voor een sterke democratie en een warme samenleving. Brussel: Politeia; 2023. https://www.politeia.be/nl/publicaties/357512-communicatie+als+bindkracht
120. Government UK. The Bletchley Declaration by countries attending the AI Safety Summit. 2023. https://www.gov.uk/government/publications/ai-safety-summit-2023-the-bletchley-declaration/the-bletchley-declaration-by-countries-attending-the-ai-safety-summit-1-2-november-2023
121. Groves K. CAPTCHA-less security. 2012. https://karlgroves.com/captcha-less-security/
122. Guyatt GH, Oxman AD, Kunz R, et al. Going from evidence to recommendations. BMJ. 2008;336(7652):1049-1051. doi:10.1136/bmj.39493.646875.ae
123. Guyatt GH, Oxman AD, Vist GE, et al. GRADE: an emerging consensus on rating quality of evidence and strength of recommendations. BMJ. 2008;336(7650):924-926. doi:10.1136/bmj.39489.470347.ad
124. Han Y, Itälä T, Hämäläinen M. Citizen Centric Architecture approach - taking e-health forward by integrating citizens and service providers. Studies in health technology and informatics. 2010;160(2):907-911. doi:10.3233/978-1-60750-588-4-907
125. Harris S. Australian social workers' understandings of technology in practice. Australian Social Work. 2022;75(4):420-432. doi:10.1080/0312407X.2021.1949025
126. Hartman-van der Laan, M. Sociaal werk in de digitale samenleving. Coutinho. 2019. https://www.coutinho.nl/nl/sociaal-werk-in-de-digitale-samenleving-9789046906590
127. Harvard Business School Online. 5 steps in the change management process. HBS Online Business Insights Blog. 2020. https://online.hbs.edu/blog/post/change-management-process
128. Health Canada. Guidance Document: Software as a Medical Device (SaMD) – Definition and Classification (Catalogue No. H164-291/2-2019E-PDF). Ottawa, ON: Health Canada. 2019. https://www.canada.ca/en/health-canada/services/drugs-health-products/medical-devices/application-information/guidance-documents/software-medical-device-guidance.html

129. Helfrich CD, Damschroder LJ, Hagedorn HJ, et al. A critical synthesis of literature on the promoting action on research implementation in health services (PARIHS) framework. Implementation Science. 2010;5(1). doi:10.1186/1748-5908-5-82
130. Henson P, David G, Albright K, Torous J. Deriving a practical framework for the evaluation of health apps. The Lancet Digital Health. 2019;1(2):52-54. doi:10.1016/s2589-7500(19)30013-5
131. HL7 FHIR Foundation. Welcome to the HL7 FHIR Foundation. 2024. https://www.fhir.org/
132. HL7 International. FHIR Release 5 (version 5.0.0). 2024. https://hl7.org/fhir/
133. Hoogendoorn P, Versluis A, Kampen S, et al. What Makes a Quality Health App—Developing a Global Research-Based Health App Quality Assessment Framework for CEN-ISO/TS 82304-2: Delphi Study. JMIR Formative Research. 2023;7. doi:10.2196/43905
134. Hui K, Gorin M, Sisti D. A Call for Greater Regulation of Digital Mental Health Technologies. AJOB Neuroscience. 2022;13(3):193-195. doi:10.1080/21507740.2022.2082595
135. Inspectie Gezondheidszorg en Jeugd. Gehandicaptenzorg worstelt met digitale vormen van zorg. 2023. https://www.igj.nl/actueel/nieuws/2023/06/16/gehandicaptenzorg-worstelt-met-digitale-vormen-van-zorg
136. Inspectie Gezondheidszorg en Jeugd. Toetsingskader Digitale Zorg – uitgebreide versie. Ministerie van Volksgezondheid, Welzijn en Sport. 2024. https://www.igj.nl/publicaties/toetsingskaders/2024/05/06/toetsingskader-digitale-zorg-uitgebreide-versie
137. International Electrotechnical Commission. IEC 62366-1:2015 – Medical devices – Part 1: Application of usability engineering to medical devices. IEC. 2015. https://www.iso.org/standard/63179.html
138. International Electrotechnical Commission. IEC 62304:2006/Amd 1:2015 – Medical device software — Software life cycle processes — Amendment 1. International Electrotechnical Commission. 2015. https://www.iso.org/standard/64686.html
139. International Medical Device Regulators Forum. Software as a medical device (SaMD). 2017. https://www.imdrf.org/working-groups/software-medical-device-samd
140. International Medical Device Regulators Forum. Software as a Medical Device Possible Framework for Risk Categorization and Corresponding Considerations. 2014. https://www.imdrf.org/sites/default/files/docs/imdrf/final/technical/imdrf-tech-140918-samd-framework-risk-categorization-141013.pdf
141. International Organization for Standardization & International Electrotechnical Commission. ISO/IEC 27001:2022 – Information security, cybersecurity and privacy protection – Information security management systems – Requirements. ISO/IEC. 2022. https://www.iso.org/standard/27001.html
142. International Organization for Standardization & International Electrotechnical Commission. ISO/IEC 27701:2019 – Privacy information management – Extension to ISO/IEC 27001 and ISO/IEC 27002 for privacy information management systems (1st ed.). ISO/IEC. 2019. https://www.iso.org/standard/71670.html
143. International Organization for Standardization. ISO 13485:2016 – Medical devices – Quality management systems – Requirements for regulatory purposes (2nd ed.). ISO. 2016 https://www.iso.org/standard/59752.html
144. International Organization for Standardization. ISO 24495-1:2023 – Plain language — Part 1: Governing principles and guidelines (1st ed.). ISO. 2023. https://www.iso.org/standard/78907.html

145. International Organization for Standardization. ISO 27269:2021. Health informatics – International patient summary. 2021. https://www.iso.org/standard/79491.html
146. International Organization for Standardization. ISO 9001:2015 – Quality management systems – Requirements (5th ed.). ISO. 2015. https://www.iso.org/standard/62085.html
147. International Organization for Standardization. ISO 9241-210:2019 – Ergonomics of human-system interaction – Part 210: Human-centred design for interactive systems. ISO. 2019. https://www.iso.org/standard/77520.html
148. International Organization for Standardization. ISO/IEEE 11073-10418:2014 – Health informatics – Personal health device communication – Part 10418: Device specialization – International Normalized Ratio (INR) monitor. ISO and IEEE. 2014. https://www.iso.org/standard/61897.html
149. International Organization for Standardization. ISO/TS 82304-2:2021 – Health software – Part 2: Health and wellness apps – Quality and reliability (1st ed.). ISO. 2021. https://www.iso.org/standard/78182.html
150. Itchhaporia D. The evolution of the Quintuple Aim: health equity, health outcomes, and the economy. J Am Coll Cardiol. 2021;78(22):2262-2264. doi:10.1016/j.jacc.2021.10.018
151. Jacob C, Lindeque J, Klein A, Ivory C, Heuss S, Peter MK. Assessing the Quality and Impact of eHealth Tools. Systematic Literature Review and Narrative Synthesis JMIR Human Factors. 2023;10. doi:10.2196/45143
152. Jacob C, Lindeque J, Müller R, et al. A sociotechnical framework to assess patient-facing eHealth tools: results of a modified Delphi process. Npj Digital Medicine. 2023;6(1). doi:10.1038/s41746-023-00982-w
153. James HM, Papoutsi C, Wherton J, Greenhalgh T, Shaw SE. Spread, Scale-up, and Sustainability of Video Consulting in Health Care: Systematic Review and Synthesis Guided by the NASSS Framework. Journal of Medical Internet Research. 2021;23(1). doi:10.2196/23775
154. Janssen R, Bodenstaff L, Gyaltsen-Lohuis E. Succesvol ondernemen met e-health: Innovatieroutes in de zorg. Hogeschool Windesheim. 2013. https://www.windesheim.nl/onderzoekspublicaties/succesvol-ondernemen-met-e-health
155. Jones RB, Stallard P, Agha SS, et al. Practitioner review: Co-design of digital mental health technologies with children and young people. Journal of Child Psychology and Psychiatry. 2020;61(8):928-940. doi:10.1111/jcpp.13258
156. Kenniscentrum Data & Maatschappij. Zorgt AI voor kansen of uitdagingen op vlak van digitale inclusie? 2024. https://data-en-maatschappij.ai/thema/digitale-inclusie
157. Kenniscentrum Digisprong. DigCompEdu: Europees referentiekader voor digitale competenties van leraren. Vlaanderen. https://www.vlaanderen.be/kenniscentrum-digisprong/themas/professionalisering/digcompedu-europees-referentiekader-voor-digitale-competenties-van-leraren
158. Kenniscentrum Digitale Zorg. Leidraad applicaties en algoritmes in de zorg. Zorgverzekeraars Nederland. https://www.zn.nl/app/uploads/2023/03/KC.DZ_Leidraad_applicaties_en_algoritmes_versie1.6-.xlsx
159. Kennisplein Gehandicaptensector. Waarom NEN 7510 relevant is voor jou! 2024. https://www.kennispleingehandicaptensector.nl/tips-tools/tips/waarom-nen-7510-relevant-is-voor-jou
160. Kidholm K, Ekeland AG, Jensen LK, et al. A MODEL FOR ASSESSMENT OF TELEMEDICINE APPLICATIONS: MAST. International Journal of Technology Assessment in Health Care. 2012;28(1):44-51. doi:10.1017/s0266462311000638

161. Kip H, Wentzel J, Kelders SM. Shaping Blended Care: Adapting an Instrument to Support Therapists in Using eMental Health. JMIR Mental Health. 2020;7(11). doi:10.2196/24245
162. Kretzschmar K, Tyroll H, Pavarini G, Manzini A, Singh I, Group NYPA. Can Your Phone Be Your Therapist? Young People's Ethical Perspectives on the Use of Fully Automated Conversational Agents (Chatbots) in Mental Health Support. Biomedical Informatics Insights. 2019;11. doi:10.1177/1178222619829083
163. Kristensen FB, Lampe K, Wild C, Cerbo M, Goettsch W, Becla L. The HTA Core Model ® —10 Years of Developing an International Framework to Share Multidimensional Value Assessment. Value in Health. 2017;20(2):244-250. doi:10.1016/j.jval.2016.12.010
164. Kruispuntbank van de Sociale Zekerheid (KSZ). Minimale normen – informatieveiligheidsbeleid. https://www.ksz-bcss.fgov.be/nl/gegevensbescherming/informatieveiligheidsbeleid
165. Label2Enable Consortium. Label2Enable: Promoting a quality label for health apps. 2024. https://label2enable.eu/
166. Lattie EG, Stiles-Shields C, Graham AK. An overview of and recommendations for more accessible digital mental health services. Nature Reviews Psychology. 2022;1(2):87-100. doi:10.1038/s44159-021-00003-1
167. Lewin S, Glenton C, Munthe-Kaas H, et al. Using qualitative evidence in decision making for health and social interventions: An approach to assess confidence in findings from qualitative evidence syntheses (GRADE-CERQual. PLOS Medicine. 2015;12(10). doi:10.1371/journal.pmed.1001895
168. Liverpool S, Mota CP, Sales CMD, et al. Engaging children and young people in digital mental health interventions: Systematic review of modes of delivery, facilitators, and barriers. Journal of Medical Internet Research. 2020;22(6). doi:10.2196/16317
169. Lopes IM, Guarda T, Oliveira P. How ISO 27001 can help achieve GDPR compliance. In: Rocha A, Pedrosa I, Cota MP, Goncalves R, eds. Proceedings of the 14th Iberian Conference on Information Systems and Technologies (CISTI 2019) (Article 8760937. IEEE Computer Society Press; 2019. doi:10.23919/CISTI.2019.8760937
170. Lucas C, Verhasselt P. Van ambitie tot adoptie: Digitaal transformeren met impact. Die Keure. 2023. https://www.diekeure.be/professional/nl/product/van-ambitie-tot
171. Maatman S. Op weg naar kwaliteitscriteria voor gemixte participatie: Een verkennend onderzoek naar de kwaliteitswaarborging van gemixte participatietrajecten [Masterthesis. 2022. https://theses.ubn.ru.nl/bitstreams/c33bf566-30d4-4e7f-9a55-aa5c9bafa11c/download
172. Mattar J, Ramos DK, Lucas MR. DigComp-Based Digital competence Assessment Tools: Literature Review and Instrument Analysis. Education and Information Technologies. 2022;27(8):10843-10867. doi:10.1007/s10639-022-11034-3
173. McDonald A, Eccles JA, Fallahkhair S, Critchley HD. Online psychotherapy: trailblazing digital healthcare. BJPsych Bulletin. 2019;44(2):60-66. doi:10.1192/bjb.2019.66
174. McGrath P, Wozney L, Rathore SS, Notarianni M, Schellenberg M. Toolkit for E-Mental Health Implementation. Mental Health Commission of Canada; 2018. https://www.mentalhealthcommission.ca/professional-resources/ementalhealth/toolkit-for-e-mental-health-implementation/
175. Mediawijs. Aan de slag als digihelper. 2022. https://assets.mediawijs.be/2022-06/mediawijs_digitaleinclusie_nl.pdf.
176. Mediawijs. Beleidstool digitale inclusie – welzijn. 2020. https://www.mediawijs.be/nl/tools/beleidstool-digitale-inclusie-welzijn

177. Mediawijs. Digitale inclusie. 2023. https://www.mediawijs.be/nl/artikels/wat-digitale-inclusie
178. Mediawijs. Hoe digitaal inclusief is jouw product of dienst? 2020. https://www.mediawijs.be/nl/tools/hoe-digitaal-inclusief-jouw-product-dienst
179. Mediawijs. Inclusion by design. https://www.mediawijs.be/nl/dossiers/inclusion-design
180. Mediawijs. Wanneer ben je mediawijs? 2024. https://www.mediawijs.be/nl/mediawijsheid
181. Medical Device Coordination Group. Guidance on qualification and classification of software in Regulation (EU) 2017/745 – MDR and Regulation (EU) 2017/746 – IVDR (MDCG 2019-11). European Commission. 2019. https://health.ec.europa.eu/system/files/2020-09/md_mdcg_2019_11_guidance_qualification_classification_software_en_0.pdf
182. MedTech Europe & COCIR. Interoperability standards in digital health: A white paper from the medical technology industry. 2021. https://www.medtecheurope.org/wp-content/uploads/2021/10/mte_interoperability_digital_health_white-paper_06oct21.pdf
183. Mental Health Commission of Canada. Toolkit for e-Mental Health Implementation. 2018. https://mentalhealthcommission.ca/resource/toolkit-for-e-mental-health-implementation/
184. Mesiäislehto M, Kivipelto M, Hiilamo H. Social workers' perceptions on clients' service needs and social work practices during the COVID-19 pandemic. Journal of Comparative Social Work. 2021;16(2):9-35. doi:10.31265/jcsw.v16i2.396
185. Mikolajczak J, Keijsers J, Henkemans OB. eHealth Analyse en SturingsInstrument (eASI. Tijdschrift Voor Gezondheidswetenschappen. 2011;89(2):78-82. doi:10.1007/s12508-011-0029-x
186. Ministerie van Binnenlandse Zaken en Koninkrijksrelaties. Implementatiekader 'Verantwoorde inzet van algoritmen'. 2023. https://www.rijksoverheid.nl/documenten/rapporten/2023/06/30/implementatiekader-verantwoorde-inzet-van-algoritmen
187. Ministerie van Volksgezondheid, Welzijn en Sport. Beslisboom betrouwbaarheidsniveaus en erkende inlogmiddelen. 2022. https://www.datavoorgezondheid.nl/documenten/2022/08/17/beslisboom-betrouwbaarheidsniveaus
188. Mishna F, Milne B, Sanders J, Greenblatt A. Social Work Practice During COVID-19: Client Needs and Boundary Challenges. Global Social Welfare. 2021;9(2):113-120. doi:10.1007/s40609-021-00219-2
189. Mozilla Foundation. Are mental health apps better or worse at privacy in 2023? 2023. https://foundation.mozilla.org/en/privacynotincluded/articles/are-mental-health-apps-better-or-worse-at-privacy-in-2023/
190. Mstiaen P, Devriese S, Pouppez C, Roberfroid D, Savoye I. Videoconsultaties in de opvolging van patiënten met een chronische somatische aandoening. Health Services Research (HSR. Brussel Federaal Kenniscentrum voor de Gezondheidszorg (KCE. Published online 2020. doi:10.57598/R328AS
191. Mulder, P. ISO normen: de betekenis en overzicht. Toolshero. 2025. https://www.toolshero.nl/kwaliteitsmanagement/iso-normen/
192. N.H.S. Digital Technology Assessment Criteria (DTAC). UK. 2021. https://transform.england.nhs.uk/key-tools-and-info/digital-technology-assessment-criteria-dtac/
193. N.Q.A. ISO 13485:2016 – Medical devices management system implementation guide. 2016. https://www.nqa.com/medialibraries/NQA/NQA-Media-Library/PDFs/NQA-ISO-13485-Implementation-Guide.pdf

194. National Association of Social Workers (nasw).Standard for Technology in Social Work Practice. https://www.socialworkers.org/Practice/NASW-Practice-Standards-Guidelines/Standards-for-Technology-in-Social-Work-Practice
195. National Institute for Health and Care Excellence. Evidence standards framework (ESF) for digital health technologies. https://www.nice.org.uk/corporate/ecd7
196. National Institute of Standards and Technology (US). The NIST Cybersecurity framework (CSF) 2.0. 2024. https://doi.org/10.6028/NIST.CSWP.29.ipd
197. Neal D, Engelsma T, Tan J, et al. Limitations of the new ISO standard for health and wellness apps. The Lancet Digital Health. 2022;4(2):80-82. doi:10.1016/s2589-7500(21)00273-9
198. Nederlands Centrum Jeugdgezondheid. Beeldbellen in de jeugdgezondheidszorg. 2023. https://www.jgzrichtlijnen.nl/praktijkmodule/praktijkmodule-beeldbellen-in-de-jeugdgezondheidszorg-2023/
199. Nederlandse Zorgautoriteit. Wegwijzer bekostiging digitale zorg. 2025. https://puc.overheid.nl/nza/doc/PUC_11953_22/
200. NELL. There’s a new sheriff in town: Richtlijnen en kwaliteitsnormen voor AI-ontwikkeling. 2022. https://nell.eu/nieuws/blog-i-theres-a-new-sheriff-in-town-richtlijnen-en-kwaliteitsnormen-voor-ai-ontwikkeling
201. Nieuwboer C. Oefenen met methodische online hulpverlening. Lectoraat Jeugd, Gezin en Samenleving. https://magazines.avans.nl/methodische-online-hulpverlening/oefenen-met-methodische-online-hulpverlening
202. Nieuwboer C. Professionele online communicatie in zorg en welzijn. Lectoraat Jeugd, Gezin en Samenleving. https://magazines.avans.nl/online-communicatie-hoe-maak-je-de-klik/cover
203. Nijs D, Drooghmans N. Mediawijsheidsnood: Nodendetectie en aanbevelingen betreffende het ondersteunen van mediawijsheid door professionals in de niet rechtstreeks toegankelijke hulpverlening. https://www.ucll.be/sites/default/files/documents/expertisecellen/eSocialWork/20190510_project_nodendetectiemediawijsheid.pdf
204. Nordesjö K, Scaramuzzino G, Ulmestig R. The social worker-client relationship in the digital era: a configurative literature review. European Journal of Social Work. 2021;25(2):303-315. doi:10.1080/13691457.2021.1964445
205. Nordesjö K, Scaramuzzino G. Digitalization, stress, and social worker–client relationships during the COVID-19 pandemic. Journal of Social Work. 2023;23(6):1080-1098. doi:10.1177/14680173231180309
206. Open Web Application Security Project OWASP. OWASP Mobile Application Security. https://mas.owasp.org/#our-mission
207. Opgroeien. Zeven B’s als analysekader om drempels in kaart te brengen en weg te werken. Vlaanderen. https://www.opgroeien.be/kennis/themas/toegankelijkheid/zeven-bs-als-analysekader-om-drempels-kaart-te-brengen-en-weg-te-werken
208. Opgroeien. Kinderen, jongeren en gezinnen blended ondersteunen. Vlaanderen. 2022. https://www.opgroeien.be/kennis/toolbox/document-kinderen-jongeren-en-gezinnen-blended-ondersteunen-een-advies-over-visie-en-positie
209. Opgroeien. Professioneel beeldbellen in welzijnswerk, sociaal werk en geestelijke gezondheidszorg. Vlaanderen. 2020. https://www.opgroeien.be/kennis/toolbox/draaiboek-professioneel-beeldbellen
210. Opgroeien. Project Parkour. Vlaanderen. 2023. https://www.opgroeien.be/over-opgroeien/projecten/parkour
211. Opgroeien. Quickscan voor onlinehulp in zorg en welzijn. Vlaanderen. 2018. https://www.opgroeien.be/kennis/toolbox/

212. Orcha. The organisation for the review of care and health apps (ORCHA). 2023. https://orchahealth.com/
213. Organisation for Economic Co-operation and Develoment (OECD). Technology and Innovation Outlook 2023: Enabling Transitions in Times of Disruption. OECD Publishing; 2023. doi:10.1787/0b55736e-en.
214. Organisation for Economic Co-operation and Development (OECD). Health. 2023. https://www.oecd.org/en/topics/health.html/
215. Osborne S. How does ISO 13485 compare to ISO 9001?. Horizon. 2022. https://www.extrahorizon.com/insights/blog-posts/how-does-iso-13485-compare-to-iso-9001
216. OWASP SAMM. Software Assurance Maturity Model. 2019. https://www.opensamm.org/
217. OWASP. OWASP Top Ten. 2021. https://owasp.org/www-project-top-ten/
218. Park SY, Nicksic Sigmon C, Boeldt D. A Framework for the Implementation of Digital Mental Health Interventions: The Importance of Feasibility and Acceptability Research. 2022. doi:10.7759/cureus.29329
219. Patel O. EU AI Act Cheat Sheet [Infographic. International Association of Privacy Professionals; 2023. https://iapp.org/resources/article/eu-ai-act-cheat-sheet/
220. Patiëntenfederatie Nederland, Ieder(in), MIND Landelijk Platform Psychische Gezondheid, PGOsupport & IKONE. Samenwerken met patiënten: Digitale zorg die werkt – 7 aanbevelingen. Participatiekompas. 2018. https://participatiekompas.nl/media/20190930_handreiking-patie__ntenparticipatie-digitale-zorg_def.pdf
221. Pattyn E, Bocklandt P. Cliëntreizen rond onlinehulp: achtergrond en aanpak. 2020. https://cdn.nimbu.io/s/0hkvgjb/channelentries/un0fsc3/files/Cli_ntreizen%20-%20achtergrond%20en%20aanpak%20-%20versie%2010%20februari%202020.pdf.
222. Pharos. Quickscan digitale vaardigheden. https://www.pharos.nl/kennisbank/quickscan-digitale-vaardigheden-van-uw-patienten/
223. Pharos. Toegankelijk en laagdrempelig beeldbellen in de zorg. https://www.pharos.nl/toegankelijk-laagdrempelig-beeldbellen-in-de-zorg/
224. Pijpers R. Alles wat u moet weten over 21e eeuwse vaardigheden. Kennisnet. 2017. https://www.kennisnet.nl/artikel/alles-wat-je-moet-weten-over-21e-eeuwse-vaardigheden/
225. Pote H, Moulton-Perkins A, Holloway-Biddle C. Competence framework for digital clinical practice: Psychological practitioners. British Psychological Society, Division of Clinical Psychology, Digital Healthcare Committee. Published online 2020. https://digitalhealthskills.com/digitalcompetencies
226. Pote H, Rees A, Holloway-Biddle C, Griffith E. Workforce challenges in digital health implementation: How are clinical psychology training programmes developing digital competences? In: DIGITAL HEALTH. 2021. doi:10.1177/2055207620985396
227. Reixach E, Andrés E, Sallent Ribes J, et al. Measuring the digital skills of Catalan health care professionals as a key step toward a strategic training plan: Digital competence test validation study. Journal of Medical Internet Research. 2022;24(11). doi:10.2196/38347
228. Richardson MX, Landerdahl Stridsberg S, Wamala Andersson S. Evidence-related requirements in Swedish public sector procurement of health and welfare technologies – a systematic review. BMC Health Services Research. 2022;22(1). doi:10.1186/s12913-022-07723-x
229. Rijksinstituut voor ziekte- en invaliditeitsverzekering (RIZIV). Zorg op afstand: telemonitoring en therapiebegeleiding bij chronisch hartfalen. RIZIV; 2024. https://www.riziv.fgov.be/nl/professionals/verzorgingsinstellingen-en-

diensten/ziekenhuizen/verzorging-in-ziekenhuizen/zorg-op-afstand-telemonitoring-en-therapiebegeleiding-bij-chronisch-hartfalen
230. RIZIV. eGezondheid. 2022. Belgium. https://www.riziv.fgov.be/nl/thema-s/egezondheid
231. Roberts CA, Smith KC, Sherman AK. Comparison of Online and Face-to-Face Parent Education for Children with Autism and Sleep Problems. Journal of Autism and Developmental Disorders. 2018;49(4):1410-1422. doi:10.1007/s10803-018-3832-2
232. S.E.R.V. Artificiële intelligentie: Internationale verkenning van de sociaal-economische impact. 2021. https://www.serv.be/sites/default/files/documenten/SERV_20210208_Informatierapport_AI_RAP.pdf
233. Saelens S, Vandecasteele A, Vanhove T, Debruyne H, Bocklandt P. Optimail: Een nieuwe methodiek voor e-mailhulpverlening. Arteveldehogeschool. 2012. https://b9e8513d6a.clvaw-cdnwnd.com/22701d8eac43dd786f280edfdcf61659/200000155-97ea798e76/Leidraad%20e-maildienstverlening%20in%20opvoedingsondersteuning.pdf
234. Safe On Web at Work. Cyberfundamentals important. 2023. https://atwork.safeonweb.be/tools-resources/cyberfundamentals-framework
235. Safe On Web at Work. Cyberfundamentals basic. 2023. https://atwork.safeonweb.be/tools-resources/cyberfundamentals-framework
236. Safe On Web at Work. Cyberfundamentals essentials. 2023. https://atwork.safeonweb.be/tools-resources/cyberfundamentals-framework
237. Safe On Web at Work. Cyberfundamentals small. 2023. https://atwork.safeonweb.be/tools-resources/cyberfundamentals-framework
238. Safe On Web at Work. Cybersecurity self-assessment. 2023. https://atwork.safeonweb.be/nl/tools-resources/self-assessment
239. San Miguel L, Obyn C, Vinck I, Meester C, Jespers V, Pouppe C. Hoe digitale medische toepassingen evalueren met het oog op terugbetaling. 2023. https://kce.fgov.be/nl/hoe-digitale-medische-toepassingen-evalueren-met-het-oog-op-terugbetaling
240. Schalken F, Obyn C, Vinck I, Meester C, Jespers V, Pouppe C. Handboek online hulpverlening. Bohn Stafleu van Loghum. 2013. https://www.bohnstafleuvanloghum.nl/product/handboek-online-hulpverlening
241. Schotte C, Broeck N. De competenties van de Belgische klinisch psycholoog: integratie van het profiel in Advies. van de Hoge Gezondheidsraad en het CanMEDS-model VVKP. 2018;(9194). https://vvkp.be/sites/default/files/TKP%202018-01_04_Competenties.pdf
242. Schueller SM, Torous J. Scaling evidence-based treatments through digital mental health. American Psychologist. 2020;75(8):1093-1104. doi:10.1037/amp0000654
243. Silberman J, Wicks P, Patel S, et al. Rigorous and rapid evidence assessment in digital health with the evidence DEFINED framework. npj Digital Medicine. 2023;6(1). doi:10.1038/s41746-023-00836-5
244. Smart Interface Design Patterns. (2023). Color contrast cheat sheet. https://smart-interface-design-patterns.com/articles/color-contrast-cheat-sheet/#wcag-21-level-aa-minimum-contrast-cheat-sheet
245. SNOMED International. 2023. https://www.snomed.org/
246. Sociaal Werk Nederland. Aan de slag met kwaliteit: Kwaliteitslabel Sterk Sociaal Werk. 2022. https://sociaalwerknederland.nl/wp-content/uploads/wpallimport/files/426c49eed5fc1361477b30522bb9bd6fe25dacf4.pdf

247. Social Care Institute for Excellence. Digital capabilities for social workers. https://www.scie.org.uk/social-work/digital-capabilities/stakeholders
248. Software and Systems Engineering Standards Committee of the IEEE Computer Society. IEEE Standard for Data Privacy Process (IEEE Std 7002-2022). IEEE. 2022. https://standards.ieee.org/ieee/7002/6898/
249. Software and Systems Engineering Standards Committee of the IEEE Computer Society. IEEE Standard Model Process for Addressing Ethical Concerns during System Design (IEEE Std 7000-2021). IEEE. 2021. https://doi.org/10.1109/IEEESTD.2021.9536679
250. Sponselee A, Til J den O, M. G, S., Metz S. V-model: technologiekwalificaties van mbo- en hbo-professionals in zorg en welzijn. Onderwijs en Gezondheidszorg. 2021. doi:10.24078/oeng.2021.4.127178
251. Stad Gent. Jaarverslag e-inclusie Gent 2024. https://stad.gent/nl/samenleven-welzijn-gezondheid/nieuws-evenementen/jaarverslag-digitale-inclusie-gent-2024
252. Steunpunt Welzijn, Volksgezondheid en Gezin. Technologische gezondheidsinnovaties Ontwikkeling van een ethisch evaluatiekader. Flanders Care. 2021. https://www.flanderscare.be/evenementen/uitnodiging-seminarie-ethische-aspecten-van-zorginnovatie
253. Stichting Koninklijk Nederlands Normalisatie-instituut (NEN). NEN 7510:2017 – Informatiebeveiliging in de zorg – Eisen voor een managementsysteem (3e ed.). NEN. 2017. https://www.nen.nl/nen-7510-1-2017-nl-232492
254. Stichting Kruispuntbank van de Sociale Zekerheid (KSZ-BCSS). Informatieveiligheidsbeleid. https://www.ksz-bcss.fgov.be/nl/gegevensbescherming/informatieveiligheidsbeleid
255. Stoyanov SR, Hides L, Kavanagh DJ, Zelenko O, Tjondronegoro D, Mani M. Mobile App Rating Scale: A New Tool for Assessing the Quality of Health Mobile Apps. JMIR MHealth and UHealth. 2015;3(1). doi:10.2196/mhealth.3422
256. Suominen J, Veikkolainen P, Kaksonen R, Voutilainen M, Haverinen J, FinCCHTA. Comparison Report of Digi-HTA and CEN/ISO TS 82304-2:2021. Finnish Coordinating Center for Health Technology Assessment (FinCCHTA; 2023. https://oys.fi/fincchta/wp-content/uploads/sites/21/2023/02/comparison-report-of-digi-hta-and-cen-iso-ts-82304-2-2021.pdf
257. Thieme A, Belgrave D, Doherty G. Machine Learning in Mental Health. ACM Transactions on Computer-Human Interaction. 2020;27(5):1-53. doi:10.1145/3398069
258. Tirions M, Raeymaeckers P, Boxstaens J, Cornille A, Gibens S, Postma Y. #sociaalwerk. Leuven: Acco; 2019.
259. Tongeren P, Bal C. Blijven vragen wat kwaliteit is. Ethische Perspectieven. 1998;8(4):311-315. doi:10.2143/epn.8.4.516892
260. Tools RRI. RRI tools. 2022. https://rri-tools.eu/
261. Trimbos-instituut. Vragenlijst Onlinehulpstempel. 2016. https://www.onlinehulpstempel.nl/files/vragenlijst.pdf
262. U.S. Department of Health and Human Services. Health Insurance Portability and Accountability Act of 1996 (HIPAA). 2021. https://www.hhs.gov/hipaa/for-professionals/index.html
263. U.S. Food and Drug Administration. Artificial Intelligence/Machine Learning (AI/ML)-based Software as a Medical Device (SaMD) Action Plan. 2021. https://www.fda.gov/news-events/press-announcements/fda-releases-artificial-intelligencemachine-learning-action-plan
264. U.S. Food and Drug Administration. Clinical Decision Support Software Guidance for Industry and Food and Drug Administration Staff. 2022.

https://www.fda.gov/regulatory-information/search-fda-guidance-documents/clinical-decision-support-software
265. U.S. Food and Drug Administration. Clinical decision support software: Guidance for industry and Food and Drug Administration staff. 2022. https://www.fda.gov/regulatory-information/search-fda-guidance-documents/clinical-decision-support-software
266. U.S. Food and Drug Administration. Global approach to Software as a Medical Device. 2022. https://www.fda.gov/medical-devices/software-medical-device-samd/global-approach-software-medical-device
267. U.S. Food and Drug Administration. Medical device safety and the 510(k) clearance process. 2023. https://www.fda.gov/medical-devices/510k-clearances/medical-device-safety-and-510k-clearance-process
268. U.S. Food and Drug Administration. Medical devices. 2023.https://www.fda.gov/medical-devices
269. U.S. Food and Drug Administration. Software as a Medical Device (SaMD). 2018. https://www.fda.gov/medical-devices/digital-health-center-excellence/software-medical-device-samd
270. Valentine L, D'Alfonso S, Lederman R. Recommender systems for mental health apps: advantages and ethical challenges. AI & SOCIETY. 2022;38(4):1627-1638. doi:10.1007/s00146-021-01322-w
271. Van Bogaert M. 101 schrijftips die je moest missen op school. Lannoo Campus. 2022. https://www.lannoo.be/nl/101-schrijftips-die-je-moest-missen-op-school
272. Van Gucht K. Guidelines for ehealth applications. imec; 2021.
273. Van Smeden M, Moons C, Hooft L, Kant I, Os H, Chavannes N. Leidraad voor kwalitatieve diagnostische en prognostische toepassingen van AI in de zorg (Versie 1.0. Ministerie van Volksgezondheid, Welzijn en Sport. 2021. doi:https://www.datavoorgezondheid.nl/wegwijzer-ai-in-de-zorg/documenten/publicaties/2021/12/17/leidraad-kwaliteit-ai-in-de-zorg-nieuw
274. Vandemeulebroucke T, Mertens E, Dernier Y, Gastmans C. Technologische gezondheidsinnovaties: Ontwikkeling van een ethisch evaluatiekader. In: Steunpunt Welzijn, Volksgezondheid en Gezin. 2021. https://cdn.nimbu.io/s/5s8z9pq/channelentries/e9e9sdv/files/2021_14_Rapport_62_SWVG_EF47_2__Ethiek.pdf?be18f3m
275. Vandooren T. Maar wij hebben toch geen klanten: Klantgericht organiseren in social profit. Owl Press. 2023. https://www.borgerhoff-lamberigts.be/owl-press/shop/boeken/maar-wij-hebben-toch-geen-klanten
276. Verplancke J, Bocklandt P. Ready to blend?! Is jouw cliënt klaar voor een blended traject? Checklist voor sociale professionals en cliënten om samen blended te werken. Arteveldehogeschool – Expertiselijn Mens, Samenleving & Digitalisering. 2023. https://b9e8513d6a.clvaw-cdnwnd.com/22701d8eac43dd786f280edfdcf61659/200001061-c6040c6042/Ready%20to%20blend%20-%2018%20oktober%202023.pdf
277. Vitalink. 2023. https://www.vitalink.be/
278. Vlaams Agentschap Innoveren & Ondernemen (VLAIO). digitalisering cybersecurity.2023 https://www.vlaio.be/nl/begeleiding-advies/digitalisering/cybersecurity?utm_campaign=%5BPCMN%5D%20CyberStart-NL&utm_medium=email&_hsmi=80113415&_hsenc=p2ANqtz-_5b_e0QR1qjbeSZFHxQvnkXR8NY0XkFZ-nVWrUib5H6MrUrWrRQ5QFqZ6I_edGrsc9S-84EJY87aRJNMYfmpQt-

WIN4MJ6JA913CEHMv65VQ5m0WA&utm_content=80113415&utm_source=hs_email
279. Vlaams expertisecentrum toegankelijkheid. Bestuursdecreet: omzetting Europese richtlijn voor de toegankelijkheid van de websites en mobiele applicaties van overheidsinstanties. Vlaanderen. 2018. https://www.vlaanderen.be/inter/toolbox-toegankelijke-steden-en-gemeenten/algemeen-bestuur-dienstverlening-en-communicatie/digitale-toegankelijkheid/bestuursdecreet-omzetting-europese-richtlijn-voor-de-toegankelijkheid-van-de-websites-en-mobiele-applicaties-van-overheidsinstanties
280. Vlaams expertisecentrum toegankelijkheid. De Richtlijnen voor Toegankelijkheid van Webcontent (WCAG) in begrijpelijke taal. Vlaanderen. https://www.vlaanderen.be/inter/toolbox-toegankelijke-steden-en-gemeenten/algemeen-bestuur-dienstverlening-en-communicatie/digitale-toegankelijkheid/de-richtlijnen-voor-toegankelijkheid-van-webcontent-wcag-in-begrijpelijke-taal
281. Vlaams expertisecentrum toegankelijkheid. Toegankelijkheidsverklaring. Vlaanderen. https://www.vlaanderen.be/inter/toolbox-toegankelijke-steden-en-gemeenten/algemeen-bestuur-dienstverlening-en-communicatie/digitale-toegankelijkheid/toegankelijkheidsverklaring
282. Vlaams expertisecentrum toegankelijkheid. Waarderingsplatform: deel uw ervaring met zorg en ondersteuning. Departement Zorg en Gezondheid. https://www.departementzorg.be/nl/waarderingsplatform-deel-uw-ervaring-met-zorg-en-ondersteuning
283. Vlaams Netwerk tegen Armoede. 24 speerpunten voor een structureel armoedebeleid in. 2023. doi:https://www.netwerktegenarmoede.be/nl/nieuws/2023/memorandum-van-het-vlaams-netwerk-tegen-armoede-verkiezingen-2024
284. Vlaamse Kwalificatiestructuur. Beroepskwalificatiedossier klinisch psycholoog. https://app.akov.be/pls/pakov/f?p=VLAAMSE_KWALIFICATIESTRUCTUUR:2::::2:P2_BK_DOSSIER_ID,P2_BKD_PROCES_ID,P2_DOCUMENT_TYPE_ID,P2_PROCES_STAP_ID,P2_FORMAT:6321,7941,82,6,PDF
285. Voka. Digitale Quickscan. https://www.voka.be/digitale-quickscan
286. Vuorikari R, Kluzer S, Punie Y. DigComp 2.2: The Digital Competence Framework for Citizens. Publications Office of the European Union; 2022. https://publications.jrc.ec.europa.eu/repository/handle/JRC128415
287. Watson L. WCAG primer. 2020. https://tetralogical.com/blog/2020/04/10/wcag-primer/
288. Wentzel J, Vaart R, Bohlmeijer ET, Gemert-Pijnen JEWC. Mixing Online and Face-to-Face Therapy: How to Benefit From Blended Care in Mental Health Care. JMIR Mental Health. 2016;3(1). doi:10.2196/mental.4534
289. Witte N, Daele T. UTAUT-vragenlijsten. Onderzoeksgroep Zorg en Welzijn. 2017. https://thomasmore.be/nl/zorg-en-welzijn-mens-en-welzijn/vragenlijst-utaut
290. World Health Organization. Global Initiative on Digital Health. https://www.who.int/initiatives/global-initiative-on-digital-health
291. World Health Organization. National eHealth strategy toolkit. 2012. https://www.who.int/publications/i/item/national-ehealth-strategy-toolkit
292. World Health Organization. Recommendations on digital interventions for health systems strengthening. 2019. https://www.who.int/publications/i/item/9789241550505
293. World Health Organization. WHO calls for safe and ethical AI for health. 2023. https://www.who.int/news/item/16-05-2023-who-calls-for-safe-and-ethical-ai-for-health#:~:text=The%206%20core%20principles%20identified%20by%20WHO%20are%3A,%286%29%20promote%20AI%20that%20is%20responsive%20and%20sustainable

294. World Health Organization. WHO outlines considerations for regulation of artificial intelligence for health. 2023. https://www.who.int/news/item/19-10-2023-who-outlines-considerations-for-regulation-of-artificial-intelligence-for-health
295. World Health Organization. WHO releases AI ethics and governance guidance for large multi-modal models. 2024. https://www.who.int/news/item/18-01-2024-who-releases-ai-ethics-and-governance-guidance-for-large-multi-modal-models
296. World Wide Web Consortium (W3C). W3C Accessibility Standards Overview. Web Accessibility Initiative (WAI). 2024. https://www.w3.org/WAI/standards-guidelines/
297. World Wide Web Consortium (W3C). Web Content Accessibility Guidelines (WCAG) 2.2. 2023. https://www.w3.org/TR/WCAG22/
298. Zantvoord J. Eindrapport onderzoek Digitale Vaardigheden. 2020. https://utrechtzorg.net/media/pages/nieuws/programma-digitaal-vaardig-levert-waardevolle-aanbevelingen-voor-zorg-en-welzijn/15fafdb5fa-1655902278/digitale-vaardigheden-onderzoeksrapport-regio-utrecht_-juli-2020.pdf
299. Zemaitaityte I, Bardauskiene R, Pivoriene J, Katkonienė A. Digital competences of future social workers: the art of education in uncertain times. Social Work Education. 2023;43(4):1078-1091. doi:10.1080/02615479.2022.2164269
300. Zhu H, Andersen ST. Digital competence in social work practice and education: experiences from Norway. Nordic Social Work Research. 2021;12(5):823-838. doi:10.1080/2156857x.2021.1899967
301. Ziegler M, Iliescu D. Measurement does not take place in a legal vacuum: Ideas regarding Regulation (EU) 2017/745 of the European Parliament and of the Council on Medical Devices. European Journal of Psychological Assessment. 2023;39(2):79-84. doi:10.1027/1015-5759/a000764
302. Zorginstituut Nederland. Evaluatie van eHealth technologie in de context van beleid. Zorg. 2017. https://www.zorgvoorinnoveren.nl/.uc/fb134d21601020dd1ae00fd5ed102b797be3b9b6c082500/Evaluatie-van-eHealth-technologie-web.pdf
303. Zorgverzekeraars Nederland. Leidraad applicaties en algoritmes in de zorg. https://www.zn.nl/dossier/digitalisering/
