# Supplementary material for: Development of a Contextualized, Research-Based Flemish Assessment Framework for Digital Care, Assistance, and Support: Delphi Study
Source: JMIR Form Res. 2026 Apr 15;10:e88512. doi: 10.2196/88512 (PMC13129510; doi:10.2196/88512)
Supplement: Multimedia Appendix 3 [file formative_v10i1e88512_app3.pdf]

Appendix 3: Classification criteria

Flag as optional if more ⅔ selected “optional,” remove if more than ⅔ selected “unnecessary,” otherwise retain as minimum.

Pillar 1: Technology

Focus	Cluster	Criteria	Minimum	Optional	Unnecessary
Provider technology		The provider of the technology is transparent and easy to find. They are reliable, and the helpdesk is free of charge and easy to reach.	19	3	
Purpose of the technology	Requirements	The intended users, the purpose of the technology and the intended use are clear.	21		
		The age range or other limitations of the users are clear.	20	2	1
		The technology is in accordance with the medical device regulation, where applicable.	21	2	
		If artificial intelligence is used in the technology, the technology is in accordance with the applicable regulations (including European AI legislation.	22	2	
		Professionals and users have been involved in the development of the technology.	10	10	2
		Appropriate scientific substantiation has been used in the development of the technology.	17	6	
	Added value	A description is available that explains the added value of using the technology, both for users and for professionals.	15	6	2
		Users are explicitly informed about the interventions that are applied.	21	2	
		Users are made aware of all efforts (including financial costs and time investment) they need to make to obtain the added value.	18	4	1
		Evidence is available to support the added value of using the technology.	11	10	1
		The information in the technology (e.g. recommendations for the user) is systematically updated. The sources of this information are disclosed to users.	17	5	1
		All funding sources of the technology are disclosed to users.	9	11	3
		Evidence is available showing that the use of the technology provides benefits. This evidence may also relate to the non-digital intervention on which the technology is based.	12	10	1
		Users get support from a professional when necessary.	18	4	1

	Support and contact	It is clear who, when, and how users can take contact. For example, whether they get an automatic answer, have contact with a professional, or with peers.	18	4	1
		It is clear who the users can contact in case of a crisis, or if they need urgent help.	19	5	
Convenience	Accessibility	The technology follows the Web Content Accessibility Guidelines (WCAG 2.2) accessibility principles (Box 3.3.1.1). This ensures that users can perceive, operate and understand all relevant information and elements of the user interface of the technology.	19	3	1
	Usability	The design of the technology is based on an understanding of users, tasks and context.<br>• All relevant user and stakeholder groups are identified.<br>• Current knowledge about contextual factors – such as user experience, usability, behaviour-change techniques, availability of certain devices and access to Wi-Fi and electricity for these groups – is used to improve sustained use in real-world settings.<br>• Users are involved throughout the design and development process, with specific attention to the most vulnerable users.<br>• The available technology is subsequently refined through user-centred evaluation.	18	4	
		There are measures in place to prevent errors and misuse of the technology.	19	3	1
		Users receive sufficient product information about the technology before purchase or installation (e.g. main functionality, use of personal data, payment, languages, information about the manufacturer, date of last update, etc.).	16	5	2
		The technology is intuitive to use. If not, a user manual is available. In addition, suitable resources are available to help users and professionals who experience problems with the technology.	19	2	1
		The technology is regularly improved and updated based on user experience and usability.	19	4	1
Deontology	Risks	The risks of the technology have been analysed and measures have been taken to manage these risks.	21	1	1
		If necessary, it is clear that the approval of a professional is required to use the technology.	20	1	2
		Users are informed about the risks, contraindications and limitations of using the technology.	20	2	1
		Safety incidents when using the technology are collected and assessed.	17	6	

	Ethics	Ethical questions are mapped out and assessed with the users and professionals.	17	5	1
		It is recommended to have the technology approved by an ethics committee or advisor	7	12	4
		The use of any advertising mechanisms in the technology is disclosed to users, and these advertisements are clearly distinguishable.	16	4	2
	Safety	The technology complies with the General Data Protection Regulation (GDPR) when personal data are processed.	22		1
		The manufacturer of the technology and all organisations that provide related services (other mobile applications, cloud computing, etc.) have implemented ISO/IEC 27001 or a recognised equivalent.	15	4	2
		A risk assessment concerning information security for the technology is available.	17	4	1
		The security of information has been included in the technology from the start of development (secure by design).	18	4	1
		Measures have been taken to ensure that all third-party software libraries and other software components for the technology are reliable and maintained.	17	4	1
		There is a process in place to prevent unauthorized access to and modifications to the source code of the technology e.g. Multi-Factor Authentication.	17	5	
		Organisational measures have been taken to ensure that personally identifiable information (PII) is processed in a way that is compatible with the explicit, legitimate purposes specified in the privacy statement.	19	3	
		User authentication, authorisation and session management are implemented to secure access to the technology.	18	4	1
		The technology transmits and stores all personally identifiable information with adequate encryption.	18	3	
		Security vulnerabilities are reported, identified, assessed, recorded, responded to, disclosed and remedied quickly and effectively.	19	3	
		Security is tested regularly, at least in the event of major changes.	16	6	
		The information security policy is easily accessible to potential customers and users.	15	6	1
Robust construction of the technology	Technical robustness	The technology works as intended.	21	2	
		There is information on all the technical requirements for the use of the technology.	15	6	1
		The technology is developed on the basis of a carefully planned and controlled process, using recognised standards, methods and tools to ensure the quality and safety of the product.	18	4	
		A secure coding standard is followed.	16	4	1
		A configuration management plan has been drawn up for the technology. This plan ensures that the technology continues to work. And that problems during use are solved as quickly as possible.	15	4	2

		The technology can handle a significant increase or spike in demand.	11	9	1
		A validation and verification plan is used for the technology.	16	4	1
		A release and deployment process has been established. This ensures that updates are carried out without any problems.	14	7	
		Technology must continue to function reliably and adapt as needs change. This requires a good maintenance process.	15	6	
		The software system has been tested<br><br>• The navigation to all parts of the technology have been tested.<br>• The technology has been tested on all software platforms on which it can be used.<br>• The technology has been tested on the most common devices of users and professionals.<br>• The cooperation with other software has been tested.<br>• The technology is tested again for each new version.<br>• If there are any problems, the previous version can be used again.<br>• Users and professionals can report technical errors.	18	4	
	Interoperability	The technology must be able to connect to other (existing) services and to other technologies.	13	5	4

Pillar 2: Organisation

Focus	Cluster	Criteria	Minimum	Optional	Unnecessary
Providing digital care, assistance and support	Technology affects the operation of the organization	The organisation describes the influence of technology on the operation and guarantees the continuity of the assistance and services, inside and outside the organisation. There is an overview of the current tasks, functions and work processes, with the technology that is used.	14	5	2
		Organisations and government clearly map all actors involved in digital work processes (within organisations, between organisations and at the level of the entire policy domain of Welfare, Public Health and Family).	9	9	3
		The different steps of the process are well aligned so that continuity can be ensured.	14	7	1
		Attention is paid to the changes that a technology entails. For example, no or fewer face-to-face activities or more support in the home environment.	15	4	2
		The organization ensures that the digital offer complements the online and offline offer already used, and does not replace it.	14	7	1
	User path	The organization makes an overview of the steps that the users take to achieve their goal. Think of waiting times, the duration of an online session, the preparations that the users have to make. This can be done, for example, with client journeys or live testing. The organization pays extra attention to vulnerable users, children and young people.	14	7	1
		The organization supports and monitors the users so that they can use the technology and achieve their predetermined goal.	19	2	1
	&Engage users, professionals, and key others	Technology has an impact on everyone involved in care, assistance and support. Therefore, from the outset, the organization supports and involves users, professionals and key others in the adoption of the technology, for example through a Change Management route.	15	4	1
	Training and supporting professionals	Appropriate education and training are provided when implementing a new technology, allowing the organisation to respond to new professional roles or new tasks for existing professionals.	21	2	
		The organization supports professionals as their work changes due to technology. She also pays attention to satisfaction when professionals use the technology.	16	7	1
	Collaboration and communication of activities	The organisation explores, implements and evaluates new forms of collaboration and communication within and outside the organisation that are required when implementing the technology.	8	13	1

	Guaranteeing and controlling quality with a system	There is a system that monitors quality at various levels. There are standards and indicators that indicate when there is a deviation from that standard.	12	8	2
		How that system is introduced can vary, but there is at least one coordinator and follow-up.	12	7	3
		It is checked how quality assurance and the monitoring system affect management and effectiveness.	5	14	1
		The organisation also follows up on the other (inter)national, regional or (inter)organisational quality standards, monitoring and registration, for example ISO 9001 and NEN-7510.	4	16	1
Structure of the policy domain of Welfare, Public Health and Family	Influence of decentralisation or centralisation requirements on the implementation of technology	Digital care, assistance and support can be offered in a decentralised way by several organisations or centralised within an organisation with specific expertise. The organisation substantiates its choice for (de)centralisation: centralisation can contribute to sustainable and high-quality digital care, assistance and support, but may also reduce accessibility.	11	7	2
	Processes that give users access to new technology	The organisation ensures that users have access to digital care, assistance and support. This can be done at individual level, for target groups and at population level.	17	4	1
		Related aspects are taken into account: social, cultural, economic, organisational, relational or geographical factors, as well as availability, accessibility, adaptability, affordability and acceptability.	16	5	1
Cost of the process	Cost of purchasing, installing, and maintaining the technology	The organisation prepares for all investments in the different stages of the process. The organisation examines how costs can be shared when different organisations are responsible for purchase costs and operating costs.	18	4	
		The organisation takes into account costs after the initial start-up, such as long-term maintenance and support.	19	1	
	Impact of technology on needs	The organization checks whether other needs arise from the user or professional as a result of the technology. For example, is less intensive supply needed? Does the technology impact the workload? The organization supports the professionals, users and relatives, if necessary.	14	7	
	Impact of the technology on the budget	The organization evaluates the impact of the technology on the available finances and budgets of government, organizations and users.	7	13	1
		The organization evaluates these financial consequences at different times.	6	14	1

Management	Management issues and opportunities related to technology	The management of the organisation is responsible for managing resources (e.g. investments), coordinating between different levels and process steps, setting objectives, monitoring and control (including how quality assurance affects management or effectiveness), and evaluation and sanctions.	17	5	
		Relevant data and information management systems linked to each of these points, as well as risk management, ethics and safety issues (for example, the safety of professionals), are implemented.	12	8	
	Decisions about which users are eligible for the technology and on what basis	The organisation identifies the key actors who decide on the use of the technology and the criteria they apply, and takes these into account.<br>This can range from the national level to the level of the individual professional.	12	6	2
Culture	Acceptance of technology	The organization examines the acceptance of the organization, professionals and users of the technology. If necessary, the organization ensures greater support.	15	6	1
	Role of stakeholders during the planning and implementation of technology	The organisation prepares an overview of potential stakeholders (e.g. software developers, hardware providers, national or regional authorities, municipalities, policymakers or decision-makers, staff representatives, other organisations in the sector, professionals or users, and patient organisations).<br>• The organisation collaborates with the desired stakeholders (e.g. by involving them in the evaluation process or as a sounding board from the start).<br>• The organisation has an interaction plan with these stakeholders.	9	9	4

Pillar 3: Professionals

Focus	Cluster	Criteria	Minimum	Optional	Unnecessary
Promoting digital inclusion and digital competences	Promoting digital inclusion	Professionals are aware that digitalisation is a permanent part of society and that users and their networks may need support with this.	18	4	1
		Professionals identify and discuss digital exclusion. They can assess whether users can safely participate digitally and whether they need support in doing so.	15	6	1
		Professionals are aware of the different challenges that digital inclusion entails and consider this important.	13	7	2
		Professionals can assess the level of digital inclusion of the user.	11	10	
	Strengthening their own digital competences	Professionals pay attention to their own media literacy and digital competences.	19	2	2
		Professionals are aware of the possible consequences of digital communication with the user, such as via social media.	19	3	1
		Professionals have good general ICT skills and can use technologies that are relevant to their specific work context.	16	5	1
		Professionals can find, assess and process digital information smoothly.	13	6	3
		Professionals make informed choices about privacy, legislative and ethical issues.	16	5	2
		Professionals continue to learn and improve their own digital competences.	14	5	2
	Being alert to strengthening users' digital competences	Professionals are concerned with strengthening the digital competences of the user and their network.	14	5	2
		Professionals contribute to the digital inclusion of the users themselves or refer them to their network or other organisations.	14	7	1
		Professionals make an overview of the digital possibilities and the Media literacy of users. They adjust their trajectory accordingly	15	8	
Providing digital care, assistance and support	Consciously selecting technology	Professionals consider the purpose of the technology, the network, and the capabilities of the users when choosing a technology.	19	3	
		Professionals involve users in choices about technology.	16	6	
		Professionals are open to the technology that users are already using.	10	9	3

	Providing digital care, assistance and support to the user and their network	Professionals can communicate digitally clearly and inclusively. They can find, select, reformulate or create general and personal digital information about and for the users.	14	6	3
		Professionals can use technology in a targeted and systematic way.	17	2	3
		Professionals use technology that supports the content of the offer, from intake or diagnosis to evaluation and aftercare.	11	8	3
		Professionals can build a professional relationship digitally.	9	10	3
		Professionals master the deontological aspects of digital communication.	19	4	
		Professionals find relevant and good technological applications and can use them in the offer.	10	9	3
		Professionals have a positive critical ambassador attitude about the use of technology and its limits. They focus on the added value of technology for the user.	9	11	2
	Securely manage digital client data	Professionals understand the importance of securely sharing digital data about and with users.	17	2	4
		Professionals can consult, analyse, interpret and enter user data in a digital file.	16	4	2
		Professionals teach the users how to work with their file, if that is part of the job.	16	4	2
		Professionals can handle the sharing of data from the user's file in a deontologically sound way.	21		1
		Professionals reflect about the purpose and use of sharing digital data through technology.	12	6	4
Helping to shape digital care, assistance and support in the organization	A vision on digital care, assistance and support	Professionals help shape the vision on digital care, assistance and support in the organization.	9	10	3
		Professionals help strengthen the confidence of other professionals and users in the digital offe	10	10	2
		Professionals contribute to practically and solution-oriented removing obstacles related to digital care, assistance and support.	10	11	2
	Constructively evaluating critical digital care, assistance and support	Professionals can constructively and critically evaluate digital care, assistance and support with sources and measuring instruments.	9	11	2
		Professionals make it clear what support is needed to provide better digital care, assistance and support.	6	14	2

		Professionals are interested in inspiring examples of digital care, assistance and support in the broader professional sector.	9	10	4
	Contributing to future digital challenges	Professionals realise that technological and digital developments in their profession will continue to evolve.<br>They are willing to stay alert to these developments, to use relevant opportunities and to reflect critically on the value of these evolutions.	12	7	3
		Professionals are willing and able to participate in projects that explore and develop new perspectives on digital action.	4	16	2
		Professionals are curious about new developments in digital practice.	1	18	3
		Professionals can reflect in a constructive and critical way on the impact and possibilities of new digital care, assistance and support, including for socially vulnerable users.	12	9	1
